# A black-winged kite improved fuzzy clustering handling imbalanced uncertain data

**DOI:** 10.1371/journal.pone.0349753

**Published:** 2026-06-08

**Authors:** Hung Tran-Nam, Ha Che-Ngoc

**Affiliations:** 1 Laboratory for Applied and Industrial Mathematics, Institute for Computational Science and Artificial Intelligence, Van Lang University, Ho Chi Minh City, Vietnam; 2 Faculty of Fundamental Sciences, Van Lang University, Ho Chi Minh City, Vietnam; 3 Applied Analysis Research Group, Faculty of Mathematics and Statistics, Ton Duc Thang University, Ho Chi Minh City, Vietnam; King Fahd University of Petroleum & Minerals, SAUDI ARABIA

## Abstract

Clustering uncertain data is a fundamental problem in data mining. Imbalance among uncertain objects significantly degrades clustering performance, as minority clusters are repeatedly overshadowed by dominant ones. Consequently, existing clustering techniques often fail due to initialisation biases and inadequate similarity modelling. This paper proposes a novel algorithm, the **B**lack-winged **K**ite **I**mproved **F**uzzy clustering for probability density **F**unctions (BKIFF), which combines an optimisation-based initialisation strategy with an enhanced fuzzy clustering framework. Specifically, BKIFF incorporates the Hellinger distance into the clustering objective to more reliably capture similarities between probability density functions (pdfs), and introduces improved membership updating and prototype estimation mechanisms tailored for uncertain and imbalanced data formulated as **I**mproved **F**uzzy clustering for probability density **F**unctions (IFF) while theoretical convergence is established. In addition, the algorithm employs Black-winged Kite Optimisation (BKO) to enhance prototype selection, improving clustering stability and convergence. As a result, comprehensive experiments with synthetic Gaussian probability distributions, skewed pdfs, and real-world image datasets demonstrate that BKIFF consistently outperforms baseline methods such as FCF, FCF-${ℒ}_{1}$, *K*MEANS, and Self-Updating. Across all three examples, BKIFF achieves near-perfect *ARI*, improving from near-zero values in highly imbalanced cases {20,50,80,100} by approximately 30–35% in moderate settings, while increasing *NMI* by about 25–95%. Additionally, it reduces computational time by approximately 95–99% compared to baseline methods. In conclusion, BKIFF demonstrates superior performance and opens up new possibilities for applications in medical diagnostics, ecological analysis, and high-dimensional uncertain data mining, particularly in imbalanced environments.

## 1 Introduction

### 1.1 Clustering for uncertain data

Clustering probability density functions (pdfs) has recently emerged as an important direction for analysing uncertain data [1,2]. Nevertheless, its underdevelopment and complexity pose significant challenges as the uncertain data becomes imbalanced, raising big questions around whether clustering techniques could tolerate this type of data well. To understand these challenges, it is vital to first understand the characteristics and sources of uncertainty in modern data.

**Fig 1:**
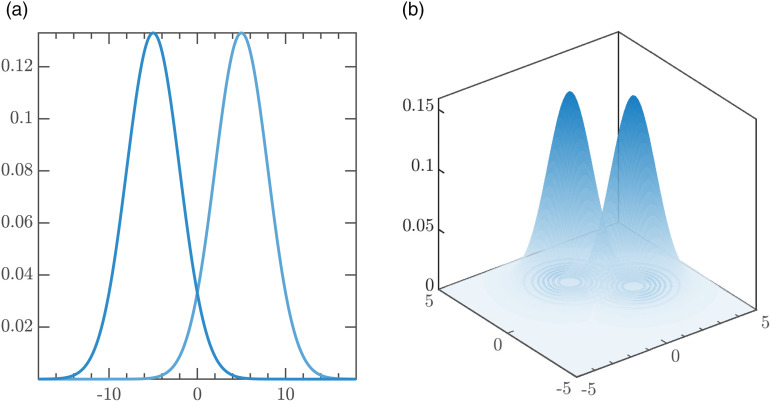
Two objects of uncertainty in (a) 1-dimension and (b) two 2-dimensional Gaussian density functions.

To begin with, data inherently contains uncertainty, a characteristic often overlooked but prevalent in modern data mining applications. Rather than each object being associated with a singular value, it is associated with an assumed level of uncertainty. In general, data uncertainty can be considered at table, tuple, or attribute level, and is usually specified by fuzzy models, evidence-oriented models, or probabilistic models presenting in massive amounts in sensor networks [3], noise in damage diagnostics experiments [4–6], population age distributions [7], and species distribution [8]. Uncertain data objects carry extra information due to repeated measurements and potential overlap with other densities. It can be in forms that sketch the probability of appearing at any position in a multidimensional space [6,9,10] as Fig 1(a) and 1(b) stage uncertainty objects in uni- and bi-dimensions, respectively. By contrast, while discrete vectors have previously yielded valuable insights, they now fail to accurately capture the object’s distribution, misrepresent ambivalent information, and overlook volatility and variation present in the data [2]. However, when uncertainty coincides with severe imbalance, the clustering task becomes inherently unstable. Large clusters dominate distance evaluations, prototypes drift toward majority densities, and minority structures are often absorbed or lost. As a result, clustering pdfs under imbalanced conditions remains an unresolved and critical practical problem.

From a methodological standpoint, clustering is a branch of unsupervised learning that analyses the hidden structure of unlabelled data into non-empty subsets (a.k.a. clusters) that share typical characteristics [11–13]. Clustering works by minimising inter-partition while maximising intra-partition similarity. Scilicet clustering aims to form compact, distinctly separated clusters. Therefore, clustering uncertain objects based on their pdfs is prevalent in numerous scenarios.

### 1.2 Related works

Over the past two decades, sporadic research has focused on developing clustering algorithms for uncertain data. These studies have built upon existing algorithms, incorporating suitable enhancements during this period. Noteworthy contributions include adaptations of partitional methods.

One of the earliest attempts to solve the problem of uncertain data clustering is Uncertain *k*-means by Chau Michael et al. [14–16]. This focuses on incorporating data uncertainty directly into the clustering process by employing the concept of expected distance, computed as the average of all possible distances weighted by an assumed (typically uniform) pdf. Empirical evidence shows that it yields significantly better clusters than ordinary *k*-means, at only a modest extra CPU cost. However, this approach is computationally expensive because it requires pairwise distance calculations across all possible realizations of the uncertain objects. To tackle this limitation, Ngai et al. [17] introduced pruning techniques to eliminate redundant computations and improve efficiency. Building upon this line of research, Gullo et al. [10,18] proposed the Uncertain *k*-medoids algorithm, which defines uncertain distance measures for both univariate and multivariate uncertain objects and achieves high clustering accuracy by uniform and binomial pdfs. UK-medoids has been experimentally shown to outperform other existing methods in terms of accuracy, regardless of the choice of uncertainty pdfs. Nevertheless, this method still suffers from high computational cost, as it requires evaluating uncertain distances between every pair of objects. To better handle cases where uncertain objects are not linearly separable in the input space, Yang et al. [19] developed the Kernel Uncertain *k*-medoids approach, which represents expected distances through kernel functions under uniform and Gaussian pdfs, revealing superior performance on several UCI datasets.

In parallel, the development of the density-based clustering algorithms includes Fuzzy Density-Based Spatial Clustering of Applications with Noise (ℱDBSCAN), Fuzzy Ordering Points to Identify the Clustering Structure (ℱOPTICS) [20]. These approaches employ fuzzy reachability-distance to estimate core-object membership and reachability probabilities. However, Zhang Xian-Chao et al. [21] proved that issues are not addressed well in ℱDBSCAN, including that it does not provide an exact function for calculating, but sampling makes it lose information, the computation is too time-consuming, and it uses a nonadaptive approach. Therefore, the probabilistic density-based uncertain data clustering algorithm (PDBSCAN) introduced definitions of core object probability and direct reachability probability, thus reducing the complexity and avoiding sampling [21]. Moreover, numerous studies employ hierarchical approaches [1,16], such as U-AHC [22], which used the Bhattacharyya coefficient as a distance measure between prototypes for Uniform, Normal, and Gamma pdfs. Additionally, Thao Nguyen-Trang et al. [1] proposed Fuzzy *c*-means for density functions, introducing both hierarchical and non-hierarchical approaches for applications in image analysis and data mining. This method is well-known for treating a continuous Gaussian density function as a clustering object.

Beyond these representative methods, more recent studies have focused on enhancing clustering performance through evolutionary and adaptive mechanisms, as summarised in Table 1. For example, Dinh Pham-Toan [23] and Tai Vo-Van et al. [24] integrated genetic algorithms and differential evolution into fuzzy clustering frameworks intended to improve optimisation capability the compactness of established cluster by Calinski–Harabasz Index. Determining the suitable number of clusters, and giving the fuzzy membership to belong to the clusters are identified. Similarly, Thao Nguyen-Trang et al. [2,25] investigated meta-heuristic optimisation strategies to stabilise clustering performance, enforce balanced cluster sizes, and a globally automatic DE-based method uses Gaussian prototypes for compactness. In addition, the possibilistic model proposed by Hung Tran-Nam et al. [26] and the self-updating fuzzy scheme introduced by Dinh Pham-Toan [27,28] emphasise the importance of adaptive mechanisms, particularly when pdfs exhibit complex and dynamic variability.

**Table 1:** Innovation of clustering probability density functions.

Author	Year	Initialisation	Distance	Methodological Approach	Automatics
Chen Jen-Hao et al. [29]	2018	–	${ℒ}_{1}$-distance	Hierarchical	Self Updating
Dinh Pham-Toan and Tai Vo-Van [27]	2020	Random	CWD	Fuzzy	Genetic Algorithm
Dinh Pham-Toan and Tai Vo-Van [23]	2022	–	${ℒ}_{1}$-distance	Fuzzy	Differential Evolutionary
Thao Nguyen-Trang et al. [2]	2023	Random	${ℒ}_{1}$-distance	Fuzzy	Meta-heuristic
Thao Nguyen-Trang et al. [25]	2023	Random	${ℒ}_{1}$-distance	Fuzzy	Differential evolution
Thao Nguyen-Trang [30]	2024	Random	${ℒ}_{2}$-distance	*k*-means	–
Hung Tran-Nam et al. [26]	2024	Fuzzy	${ℒ}_{1}$-distance	Possibilistics	–
Dinh Pham-Toan [28]	2025	–	Kullback–Leibler divergens	Fuzzy	Self-Updating
Proposed	–	*Optimisation*	*Hellinger*	*Improved Fuzzy*	–

### 1.3 Research gap

Despite research endeavours, some challenges remain. Even though optimisation methods might be sufficient for many practical applications, they rarely address imbalance as a precise case [2]. In addition, non-optimisation methods are susceptible to the choice of initial centres. Moreover, many of these studies do not address the imbalance among clusters, a condition in which the distribution of elements is more extensive or uneven than in other clusters, which occurs naturally in the real world, including uneven species densities in each survey area and low-probability diseases. The density function cluster analysis has to unveil deeper insights to recognise these imbalanced structures. Traditional methods tend to converge on the centres of the clusters with more significant numbers for two reasons: initialising the input centre density function and designing the objective function (OF) of the resulting clusters to be equal. As the Imbalanced Ratio (*IR*) increases, the probability of initialising two density functions belonging to two separate clusters to explore the cluster with smaller numbers becomes lower. This may lead to misclassification of fault, eventually resulting in a breakdown. Prior work, such as [31–33] in certain data mining, has noted a tendency to shift prototypes. Xiong Hui et al. [34] analysed the reasons for this phenomenon using the OF. They showed that *k*-means tends to evenly split the samples, even when the number of samples per cluster is large. Liang Jiye et al. [35] explain the problem of shifting the prototypes in the case of fuzzy *c*-means for clustering imbalanced data. Pu Yue et al. [31] get many clustering improvements with the siphon effect and prove the convergence of their work [2,25] *These works collectively show a clear research effort toward improving robustness; no research has contemplated a clustering technical approach in the presence of imbalanced uncertain data*. Without mechanisms to handle imbalances, clustering results become unreliable and biased toward dominant groups. A dedicated approach is therefore required to recover meaningful cluster structures in such situations.

### 1.4 Contributions

Fortunately, these limitations can be addressed through adaptive initialisation methods [36], and clustering designed explicitly for this situation. Given the aforementioned gaps, this study introduces a novel approach called **B**lack-winged **K**ite optimisation **I**mproved **F**uzzy for probability density **F**unctions (BKIFF). Specifically, the study’s innovations and contributions are listed fourfold.

- *First*, we establish a two-phase cluster analysis algorithm called **B**lack-winged **K**ite optimisation **I**mproved **F**uzzy for probability density **F**unctions (BKIFF). Specifically, all existing works aim to automate the OF entirely. They will provide the resulting label with the optimal OF. However, we target a slightly different problem: automating only the pre-processing step, rather than randomly, and then combining it with an improved fuzzy algorithm that continues to move the initialisation toward the prototype of the cluster containing them. This way, we can customise the OF and its starting condition step. This can be used independently of the core as an initialisation clustering algorithm.
- *Second*, we use the Hellinger metric as the similarity measure to capture distribution differences between objects. The sensitivity of two overlapping density functions to the similarity metric is effective both in theory and in practice.
- *Third*, we provide a robust theoretical foundation for the development of **I**mproved **F**uzzy for probability density **F**unctions (so-called IFF) by utilising Lemmas 1 and 2 in conjunction with the Zangwill theorem. It clarifies the algorithm’s convergence and stimulates the analysis of its effectiveness while addressing associated computational challenges.
- *Fourth*, we will conduct extensive experiments using synthetic and image datasets and compare our results with those of the compared algorithms. Specifically, we will examine the effect of the BKIFF algorithm by analysing the relationship between the imbalance rate and membership values. We will explore the factors contributing to a polarisation phenomenon in the rise of *IR*. Objects in larger classes tend to have significantly higher membership values, which can cause clustering algorithms to converge incorrectly and result in errors.

These contributions are contextualised within the comparative analysis presented in Table 1, thereby demonstrating their timeliness. The reestablishment of theory is a critical and novel step that highlights differences in initialisation, distance metrics, uncertainty handling, and automation across existing methods and our proposal. Last but not least, applications are novel to the literature on uncertain/imbalanced clustering, offering practical pathways in the ecology and remote sensing domains.

### 1.5 Organisation

The rest of this paper is organised as follows. First, Preliminaries section introduces the type of uncertain data and its related concepts on fuzzy clustering. Next, Methodologies and Materials section focuses on the imbalanced clustering method and its convergence. The experiments and analysis were then conducted on three datasets and an image application provided at Numerical Examples section. Next, Main Results section shows some effectiveness of the proposed method. Then, Discussion section presents some pros and cons. Finally, Conclusion section gives a summary.

## 2 Preliminaries

First, we summarise the notations commonly utilised in Table 2.

**Table 2:** Notations and their definitions.

Notations	Description
*n*	The number of all objects
*c*	The number of clusters
ℱ	Set of uncertain density functions
***H***	Hessian matrix
${ℋ}^{2}$	Hellinger distance
*OF*	Objective function of initialisation step
*J*_*IFF*_	Objective function of clustering method
${\mathbb{R}}^{d}$	*d*-dimension vector
*IR*	Imbalanced ratio
IQR	interquartile range
Ω	measurable space

### 2.1 Uncertain objects

**Definition 1 (multivariate uncertain object).** *A multivariate uncertain object* ***o*** *is defined as a pair* (***R***, *f*)*, where* $R\subseteq {\mathbb{R}}^{d}$ *is a d-dimensional region in which* ***o*** *is defined, and* $f:{\mathbb{R}}^{d}\to {\mathbb{R}}_{\geq 0}$ *is the joint pdf representing the uncertainty of* ***o*** *over* ***R*** [22].

**Definition 2 (univariate uncertain object).** *A univariate uncertain object o is defined as a pair (R, f), where* R⊆ℝ *is a one-dimensional region in which o is defined, and* $f:\mathbb{R}\to {\mathbb{R}}_{\geq 0}$ *is the pdf of o* [22].

This paper exploits this continuous pdf to represent uncertain data. Assuming the space of Borel probability measures on a measurable space $\Omega \subseteq {\mathbb{R}}^{d}$, assuming $ℱ=\{f_{1}(x)\;,f_{2}(x)\;,\ldots \;,f_{n}(x)\}$ is a family of *n*-absolutely continuous Lebesgue pdfs. Fig 1 shows the two objects of multi- and univariable uncertainty under the Gaussian density functions.

### 2.2 Similarities of density function

Many papers have investigated the format of measures of density functions as vague and uncertain data. However, the conversion is problematic, as only ${ℒ}_{p}$ [16] and the Hellinger metric affect the convergence task. Although satisfying all three distance properties, ${ℒ}_{p}$ is usually computed by numerical methods, and the complexity can quickly go beyond control with the increased dimension. We propose the Hellinger metric as a distance measure to overcome the unwarranted limitations amid that chaos.

**Definition 3 (Hellinger distance).** *Given two probability distributions F*_*1*_ *and F*_*2*_ *on a measurable space X and let f*_*1*_ *and f*_*2*_ *represent the Radon–Nikodym derivatives of F*_*1*_ *and F*_*2*_*, on the same* σ*-field, respectively. The Hellinger distance is defined as*


$$\begin{array}{c} {ℋ}^{2}(f_{1}\;,f_{2})=2\times \left(1-\int_{\Omega }\sqrt{f_{1}(x)f_{2}(x)}\;dx\right). \end{array}$$ (1)

When two pdfs have high overlap, the Hellinger distance declines and vice versa. ${ℋ}^{2}$-distance has domain in [0,2] [37].

## 3 Methodologies and materials

### 3.1 Methodologies

#### 3.1.1 The fuzzy partition space.

Let the dataset $ℱ=\{f_{1}\;,f_{2}\;,\ldots \;,f_{n}\}$ be a family of *n*-unlabelled pdfs (*n* ≥ 2) with distribution *F* and $\Theta^{\left(t\right)}=\{\theta_{1}^{\left(t\right)}\;,\theta_{2}^{\left(t\right)}\;,\ldots \;,\theta_{c}^{\left(t\right)}\}$ be a sequence of *c*-representative density functions (2 ≤ *c* ≤ *n*) at the *t* iteration. With *c*-cluster given such that each subgroup represents natural substructure in ℱ,


$$\begin{array}{c} 0\leq \mu_{ij}\leq 1,i\in {\mathbb{N}}_{\leq c}\;,j\in {\mathbb{N}}_{\leq n}, \end{array}$$ (C1)

$$\begin{array}{c} \sum_{i=1}^{c}\mu_{ij}=1\;,j\in {\mathbb{N}}_{\leq n}, \end{array}$$ (C2)

$$\begin{array}{c} 0<\sum_{j=1}^{n}\mu_{ij}<n\;,i\in {\mathbb{N}}_{\leq c}. \end{array}$$ (C3)

The (C1) are interpreted as the membership degree of the *j*-th density function to the cluster relative to all other clusters. For the (C2), this is a requirement that no cluster, represented as clusters of ℱ, is empty. In (C3), the partitioning cluster is no less than *c*.

#### 3.1.2 Improved fuzzy clustering for density function.

The OF of the IFF clustering is designed to balance two competing goals, including minimising the Hellinger distance between each density function and its associated cluster prototype, and preventing large clusters from dominating the optimisation process via the cardinal weight factors. The OF of the IFF is defined as follows


$$\begin{array}{c} \text{Minimise}\;:\;J_{IFF}(U\;,\Theta \;;ℱ\;,c\;,m)=\sum_{i=1}^{c}\sum_{j=1}^{n}\frac{1}{\omega_{i}}\mu_{ij}^{m}{ℋ}^{2}(\theta_{i}\;,f_{j}). \end{array}$$ (2)

$$\begin{array}{c} S.t.\;:\;(C1)\;,(C2)\;,(C3), \end{array}$$

where the distance ${ℋ}^{2}$ is the square of the Hellinger distance between the *j*-th datum pdf *f* and the *i*-th prototype θ. The weights $\omega_{i}$ (so-called *cluster size*) are non-negative, computed by fuzzy intra-cluster distance.


$$\begin{array}{c} \omega_{i}=\frac{1-n_{i}/n}{1-\min\{n_{i}/n\}}\;,0\leq \omega_{i}\leq 1, \end{array}$$ (3)

where, *n*_*i*_ is the number of points in cluster *i*-th after defuzzification. Then, this OF is codetermined by the distance to each cluster prototype and the cluster’s size.

Without such weighting, large clusters would disproportionately affect the objective function, suppressing smaller ones. The larger the value of $\omega_{i}$, the smaller the size of the cluster *i*-th. Dividing by $\omega_{i}$ helps ensure that every cluster has a balanced influence in the computation and adjusts each cluster’s contribution to the OF, preventing clusters with more elements from entirely dominating the optimisation process. Fig 11 illustrates the difference between two clusters having different cluster sizes. In ideal balanced cases, equal cluster weights $\omega_{1}=\omega_{2}=1.00$ lead to symmetric membership curves. Meanwhile, as the imbalance becomes more severe, IFF adaptively reduces the influence of the dominant cluster (e.g., $\omega_{1}=0.10$ for *IR* = 10), requiring objects to be much closer to the larger cluster centre to obtain high membership. In other words, only objects extremely close to a large cluster have a chance of being assigned to it.

#### 3.1.3 Initialisation the prototypes.

According to the findings of Celebi et al. [36], random methods are straightforward to understand and implement; however, they tend to be ineffective and unreliable. Additionally, although these methods have low overhead, they do not provide considerable time savings, as they frequently result in slower convergence.

Therefore, this paper used Black-winged Kite (*Elanus caeruleus*) as an optimal solution as its unique biological heuristic features with adaptability, intelligent behaviour, and predatory [38,39] published by Wang Jun et al. [40]. The initialisation step aims to identify density functions that are maximally separated in the Hellinger metric, encouraging the algorithm to start from representatives that capture the data’s global structure rather than relying on random or redundant choices. We proposed the OF to locate the best positions *i* from the data such that the distance to the others is optimal.


$$\begin{array}{c} OF=-\min_{i=1}^{d}\{\min_{j=1}^{i-1}\{{ℋ}^{2}(f_{i}\;,f_{j})\}\}, \end{array}$$ (OF)

where, ${ℋ}^{2}(f_{i}\;,f_{j})$ is the squared Hellinger distance by Eq. (1) between any two density functions with two indices i≠j.

The concept is new but straightforward: We aim to optimise the process of identifying *c* clusters with the maximum pairwise distance. Following the search algorithm, we will identify objects in the data that are ideal candidates for selection as initial prototypes. This approach allows us to forgo calculating the pairwise distance matrix and instead rely on the optimisation algorithm to search. The proposed method is quite similar to *k*-means^++^ [41], however, optimised. Algorithm 1 implements the BKO process.

The application of BKO is still in the initial exploration stage [42]. This study applies BKO to fully utilise its advantages in exploration and exploitation to identify optimal design parameters by effectively exploring the design space and determining the best solution. This innovative application expands BKO’s scope and provides a new optimisation method for the initial pdf clustering.

**Algorithm 1 Black-winged kite Optimisation for Initialising Prototypes**

**Require:** The potential solutions *pop*, maximum number of iterations *T*, and variable dimension *d*.

**Ensure:** the best quasi-optimal prototypes Θ

1:  Initialisation of the position of Black-winged kites and evaluation of the optimal OF

2:  Calculate the fitness value of each Black-winged kite

3:  **while** *t* < *T* **do**

4:   **if** *p* < *r* **then**    ▷ Attacking behaviour

5:    $y_{i,j}^{\left(t+1\right)}=y_{i,j}^{\left(t\right)}+n\left(1+\sin(r)\right)\times y_{i,j}^{\left(t\right)}$

6:   **else**

7:    $y_{i,j}^{\left(t+1\right)}=y_{i,j}^{\left(t\right)}+n(2r-1)\times y_{i,j}^{\left(t\right)}$

8:   **end if**

9:   **if** *F*_*i*_ < *F*_*ri*_ **then**    ▷ Migration behaviour

10:   $y_{i,j}^{\left(t+1\right)}=y_{i,j}^{\left(t\right)}+C(0,1)\times \left(y_{i,j}^{\left(t\right)}-L_{j}^{\left(t\right)}\right)$

11:  **else**

12:   $y_{i,j}^{\left(t+1\right)}=y_{i,j}^{\left(t\right)}-C(0,1)\times \left(m\times y_{i,j}^{\left(t\right)}-L_{j}^{\left(t\right)}\right)$

13:  **end if**

14:  **if** $y_{i,j}^{\left(t+1\right)}<L_{j}^{\left(t\right)}$ **then**    ▷ Select the best individual

15:   $loc_{best}=y_{i,j}^{\left(t+1\right)}\;,F_{best}=f\left(y_{i,j}^{\left(t+1\right)}\right)$

16:  **else**

17:   $loc_{best}=L_{j}^{\left(t\right)}\;,F_{best}=f\left(L_{j}^{\left(t\right)}\right)$

18:  **end if**

19: **end while**

20: **return** $\Theta =\{\theta_{loc_{best}}\}$ and *F*_*best*_

At the end of the algorithm, we obtain the initialised prototypes Θ inferred from the immanent data. The best individuals are selected as the initial prototypes serving as the encoded input for IFF.

**Remark 1 (Initialisation phase).** *In the Initialisation step, the matrix* *BK*_*pop*,*dim*_ *represents the location of every Black-winged kite. Then, we distribute the position of each Black-winged kite as uniform, i.e.,* $loc_{i}=BK_{lb}+𝒰(BK_{ub},BK_{lb})$*.* *BK*_*lb*_ *and* *BK*_*ub*_ *are the lower and upper bounds of i-th Black-winged kites in the j-th dimension.*

**Remark 2 (Attacking behaviour).** *In step 1,* $y_{i,j}^{\left(t\right)}$ *and* $y_{i,j}^{\left(t+1\right)}$ *represent the position of the i-th Black-winged kites in the j-th dimension in the iteration t and (t + 1)-th, respectively; r is a random number that ranges from 0 to 1, and p is a constant value of 0.9 for achieving better results* [40]; *T is the entire number of iterations, and t is the number of iterations that have been completed so far; and* $n=0.01\times e^{-2\times (t/T{)}^{2}}$.

**Remark 3 (Migration behaviour).** *In step 2,* $L_{j}^{\left(t\right)}$ *denotes the leading scorer of the Black-winged kites in the j-th dimension of the t-th iteration;* $F_{i}\;,F_{ri}$ *define the current position and the fitness value of the random position obtained by any Black-winged kite in the t-th; C(0,1) represents the Cauchy mutation* [43]; *and* m=2×sin(r+π/2)*.*

### 3.2 Assumptions

The established assumptions underlying the data input and the proposed algorithm include the following considerations

- The input pdfs do not contain excessive noise and exhibit overlapping properties, ensuring the feasibility of the clustering process.
- The input uncertainty ℱ must be represented as a continuous density function within the common measurable space $\Omega \subseteq {\mathbb{R}}^{d}$; densities are evaluated on finite grids *h* = 0.01 ensuring integrals in Hellinger distance can be controlled. Empirical datasets are transformed into pdfs using kernel density estimation.
- Clusters may be imbalanced, however, the *IR* is finite; there exists no empty or vanishing cluster.
- The number of given clusters *c* still should be determined.

### 3.3 The stage of the proposed algorithm

Algorithm 2 below outlines the process of the proposed two-stage method.

**Algorithm 2 The Black-winged Kite Improved Fuzzy clustering for pdfs algorithm (BKIFF)**

**Require:** The converted density functions data ℱ, *c* number of cluster.

**Ensure:** The membership degree ***U*** and prototypes Θ.

Initialisation of the *c*-prototypes $\Theta_{0}$ using the Alg. (1).     ▷ Initialisation

2: **while** *t* < *MaxIter* and $\|U^{\left(t+1\right)}-U^{\left(t\right)}<\varepsilon \|$ **do**

Update the partition matrix ***U***^(*t*+1)^.     ▷ Fix the membership degree $\mu_{ik}$ For $I_{j}=\{i\in {\mathbb{N}}_{\leq c}\;|\;f_{j}=\theta_{i}\}$

• In case of $I_{j}=\emptyset$, then

$$\begin{array}{c} \mu_{ij}^{\left(t+1\right)}=\frac{\omega_{i}}{\sum_{s=1}^{c}{\left({ℋ}^{2}(\theta_{i}\;,f_{j})/{ℋ}^{2}(\theta_{s}\;,f_{j})\right)}^{\frac{1}{m-1}}}, \end{array}$$ (4)

• In case of $I_{j}\neq \emptyset \;,i\in I_{j}$, then $\mu_{ij}=\frac{1}{|I_{j}|}$,

• In case of $I_{j}\neq \emptyset \;,i\notin I_{j}$, then $\mu_{ij}=0$.

4: Update the set *c*-prototypes $\Theta^{\left(t+1\right)}$     ▷ Fix the prototype $\theta_{i}$

$$\begin{array}{c} \theta_{i}^{\left(t+1\right)}={\left(\frac{\sum_{j=1}^{n}{\left(\mu_{ij}^{\left(t+1\right)}\right)}^{m}\sqrt{f_{j}(x)}}{\sum_{j=1}^{n}{\left(\mu_{ij}^{\left(t+1\right)}\right)}^{m}}\right)}^{2} \end{array}$$ (5)

Update the cluster size $\omega_{i}$     ▷ Fix the cluster size $\omega_{i}$

$$\begin{array}{c} \omega_{i}^{\left(t+1\right)}=\frac{1-n_{i}/n}{1-\min\{n_{i}/n\}} \end{array}$$ (6)

6: **end while**

At the end of the Algorithm 2, we receive the identifier of each uncertain object about the cluster to which it belongs. In **Step 2**, we obtain the fuzzy-partition matrix ***U*** resulting from the last iteration and prototypes $\Theta =\theta_{i}$ of the *i*-th cluster.

**Remark 4.** *An adequate stopping criterion for the algorithm is that the maximum absolute difference between elements of the partition matrix in two consecutive iterations be lower than a given positive threshold* ε*. A standard setting adopted in this paper is* $\varepsilon =10^{-6}$*. The maximum number of iterations, MaxIter, is set to 200. The defuzzification of this BKIFF is a final crisp assignment from membership for label reporting, i.e.,*


$$\begin{array}{c} c(f_{j}(x))=arg\max_{i}\{\mu_{ij}\}. \end{array}$$

**Remark 5.** *The parameter m, the so-called fuzzifier, controls the extent of membership sharing between fuzzy clusters as m approaches 1.0 from above, the partition matrix tends to be crisp (*$\mu_{ij}=0$ *or 1). On the other hand, the larger m is, the fuzzier the resulting partition. Usually, m = 2.0 is chosen by reference of* [1,24,44]. *In this paper, the set of m = {1.1, 1.5, 2.0, 2.5, 3.0, 5.0, 10} will be investigated in all the experiments presented.*

**Fig 2:**
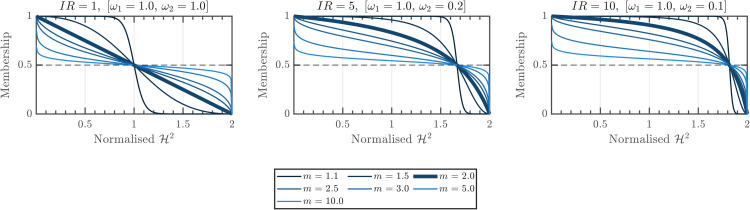
The variation of fuzziers with different simulations of ${ℋ}^{2}$. The lighter the blue, the greater the representation, the larger the value of *m*.

Fig 2 illustrates the influence of the fuzzifier parameter *m* and the cluster *IR* on the membership distribution in IFF using normalised ${ℋ}^{2}$-distance. The three subplots correspond to increasing *IR* values of 1, 5, and 10. Varying *m* from 1.1 to 10 reveals its role in controlling fuzziness, indicating lower values sharpen boundaries, whereas higher values yield softer transitions.

**Fig 3:**
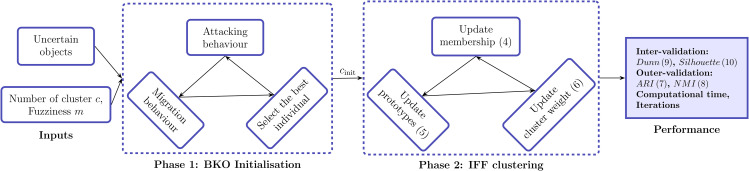
Framework of proposed BKIFF.

In particular, $\omega_{i}$ plays a key role in ensuring the uniform effect. As *IR* increases, $\omega_{i}$ automatically reduces the influence of the large cluster, forcing points that want to be assigned high membership to this cluster to be very close to the cluster centre. This phenomenon causes the membership curves to shift to the right on the distance axis, i.e., the large cluster is restrained and can no longer absorb all the points far away. As a result, points near the small cluster still maintain significant membership. The flowchart of the proposed BKIFF is shown in Fig 3. We set up the ability to reproduce seeds in controlled iterations through each repetition.

### 3.4 Convergence of IFF

For clarity, the convergence analysis is structured into a sequence of lemmas and theorems applied to Zangwill’s theorem. The convergence proof shows that the OF *J*_*IFF*_ decreases monotonically with each iteration, and that the sequence of solutions converges to a stationary point. Specifically, Lemma 1 and Lemma 2 formalize the update operators *T*_1_ for the membership matrix and *T*_2_ for the prototype functions, providing closed-form minimizers for each variable block. Theorem 2 establishes that each complete iteration of $T_{IFF}=T_{2}\circ T_{1}$ results in a strict decrease of the OF. Theorem 3 demonstrates the continuity of the updates, while Theorem 1 ensures the compactness of the iterates. Collectively, these results culminate in Theorem 4, which confirms that the sequence converges to a stationary point of the *J*_*IFF*_.

**Fig 4:**
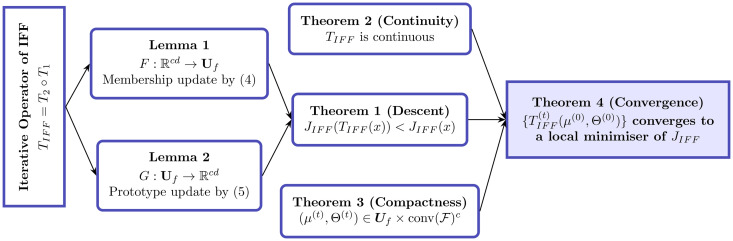
The logical flow of the convergence analysis of the proposed IFF algorithm.

Thus, the IFF method incrementally refines the cluster prototypes and the membership matrix until no further improvement can be achieved. Complete analytic proofs are available in the Supplementary Information A. Fig 4 provides a summary of the dependencies and an overview of the convergence mechanism.

### 3.5 Time complexity analysis of the BKIFF

We first discuss the runtime complexity of the BKO. The maximum number of iterations (*T*_*BKO*_), population size, and dimension (*d*) are strongly connected to the position initialisation, fitness value computation, and position update components that determine the computational complexity of the time component of BKO. Consequently, its temporal computational complexity is as follows. It takes $𝒪(T_{BKO}\times M)$ time to initialise the population, it takes 𝒪(M) to compute the fitness value, and $𝒪(T_{BKO}\times M\times d)$ to update the location. As a result, the BKO’s overall time complexity is $𝒪(M\times (T_{BKO}\times (T_{BKO}\times d+1)))$ based on Wang Jun et al. [40].

Next, IFF complexity is investigated. The update of the fuzzy membership matrix requires computing (*n* × *c*) Hellinger distances, yielding a complexity of 𝒪(n×c). The prototype update step aggregates across *n* objects in each of *c* clusters, each in *d*-dimensional space, resulting in 𝒪(n×c×d). The recalculation of cluster sizes also takes 𝒪(n×c). Therefore, the computational cost per iteration is 𝒪(n×c×(1+d)). Over *T*_*IFF*_ iterations, the overall time complexity of IFF is 𝒪(T×n×c×(1+d)).

In conclusion, the total time complexity of the BKIFF algorithm is the combination of both phases and can be expressed as $𝒪(M(T_{BKO}(1+d)+1)+T_{IFF}\times n\times c\times (1+d))$.

### 3.6 Some validity measure indexes for clustering solution

#### 3.6.1 Adjust Rand Index.

The Adjusted Rand Index (*ARI*), introduced by Hubert Lawrence and Arabie Phipps [45], extends the Rand Index to account for chance. It is widely recommended as the index of choice for assessing the agreement between two partitions in clustering analysis, even when the number of clusters differs. The *ARI* of results *M*-partition and its *C* reality class is computed as 7.


ARI(M,C)=∑ij(nij2)−[∑ij(mi2)∑ij(cj2)]/(n2)12[∑ij(mi2)+∑ij(cj2)]−[∑ij(mi2)∑ij(cj2)]/(n2). (7)

In this context, (nij2) represents the number of pairs of elements that are grouped in the same cluster in both partitions, designated as *M* and *C*. The expression ∑ij(nij2) quantifies the total agreement between the clusters in both partitions. Meanwhile, ∑ij(mi2) indicates the number of pairs within each cluster of partition *M*, regardless of how those elements are grouped in partition *C*. Finally, (n2) represents the total number of possible pairs in the dataset, where *n* is the total number of elements.

#### 3.6.2 Normalized mutual information.

Mutual information reveals the reduction in entropy of class labels when the cluster labels are known [46].


$$\begin{array}{c} NMI(M,C)=\frac{2\sum_{k=1}^{M}\sum_{h=1}^{C}\frac{n_{k}^{h}}{n}\log\left(\frac{n_{k}^{h}N}{\sum_{i=1}^{M}n_{i}^{h}\sum_{i=1}^{C}n_{k}^{i}}\right)}{H_{M}+H_{C}}, \end{array}$$ (8)

where $n_{h}^{k}$ is the number of objects in cluster *k* belonging to class *h*; H(·) represents entropy, i.e., $H(X)=-\sum_{x\in X}p(x)\log p(x).$

#### 3.6.3 Dunn index.

Given a partition $Z=\{Z_{1},\ldots ,Z_{k}\}$ of the dataset, the Dunn Index is defined as [47]


$$\begin{array}{c} Dunn(Z)=\frac{\min_{1\leq i<j\leq k}\{\delta (Z_{i},Z_{j})\}}{\max_{1\leq \ell \leq k}\{\Delta (Z_{\ell })\}}, \end{array}$$ (9)

where, $\delta (Z_{i},Z_{j})$ denotes the inter-cluster distance between clusters *Z*_*i*_ and *Z*_*j*_, and $\Delta (Z_{\ell })$ denotes the intra-cluster diameter of cluster $Z_{\ell }$. Common choices are the single-link distance $\delta_{\min}(Z_{i},Z_{j})=\min_{i\in Z_{i},j\in Z_{j}}\{ℋ(f_{i},f_{j})\}$ for δ and the complete-link diameter $\Delta_{\max}(Z_{\ell })=\max_{i,j\in Z_{\ell }}\{ℋ(f_{i},f_{j})\}$ for Δ(·).

#### 3.6.4 Silhouette coefficient.

The Silhouette Coefficient captures both intra-cluster compactness and inter-cluster separation. For every pdf object *f* assigned to cluster *Z*_*i*_, the silhouette value *s*(*f*) is defined as [48]


$$\begin{array}{c} s(f)=\frac{b(f)-a(f)}{\max\{a(f),b(f)\}}, \end{array}$$

where, $a(f)=\frac{1}{|Z_{i}|-1}\sum_{i\in Z_{i},\;j\neq i}ℋ(f_{i},f_{j})$ is the average distance from *f*_*i*_ to all other objects within its own cluster, and $b(f)=\min_{j\neq i}\{\frac{1}{|Z_{j}|}\sum_{j\in Z_{j}}ℋ(f_{i},f_{j})\}$ is the smallest average distance from *f*_*i*_ to objects in any other cluster. The global Silhouette Coefficient of a partition *Z* is the mean of all individual silhouette values


$$\begin{array}{c} Silhouette(Z)=\frac{1}{n}\sum_{f\in ℱ}s(f)\in [-1,1]. \end{array}$$ (10)

In conclusion, the *ARI* lies in the range [−1,1], while, *NMI* are symmetric measures that lie in the range [0,1], in which value 1.0 is perfect agreement between *M* and *C*, vice versa. The Dunn Index is non-negative and unbounded above; in practice, values are typically in [0, ∞), with larger values indicating better clustering quality. *Silhouette* score lies on [−1, +1] with values close to +1 indicating that points are well clustered, values near 0 suggesting overlapping or ambiguous assignments, and negative values revealing possible misclassifications.

#### 3.6.5 Computational time.

Moreover, to assess the computational complexity, their median execution time (in seconds) and the total IFF iterations (number of updated steps) are reported. We consider the BKIFF algorithm’s start time to be from the initialisation with BKO to the completion of the IFF run, in seconds. The baseline comparison algorithm is computed in parallel after the density function data is fed in. In other words, pdfs input extracted, including image-related pipelines, are not counted in the algorithm runtime.

## 4 Numerical examples

In this work, we tackle the subject from an academic research standpoint. To explore the behaviours of the proposed algorithm in standard problems, synthetic data are first generated (Examples 1 and 2). The BKIFF is then expanded to benchmark image clustering (Example 3). Furthermore, we apply the proposed method to segment a real-world Landsat image, with its grey pixels extracted to pdfs.

**Example 1.** We simulate a Gaussian pdf data in real numbers with two a priori clusters. The general role of this artificial data is to investigate the stability of the proposed algorithm under highly imbalanced constant scenarios, with *IR*s ranging from 10 to 100. The larger the *IR*, the higher the number of clusters 1 and 2. Specifically, the generated data are means and standard deviations of the general formula in each cluster, respectively.


$$\begin{array}{c} \begin{array}{c} \text{Cluster }1:f(x\;|\;\mu \;,\sigma^{2})\sim 𝒩(\mu_{1}\;,\sigma_{1}^{2})\;,\text{where,}\\ \;\mu_{1}\sim 𝒩(-5.0\;,0.5^{2})\;,\sigma_{1}^{2}=3.0^{2},\\ \text{Cluster }2:f(x\;|\;\mu \;,\sigma^{2})\sim 𝒩(\mu_{2}\;,\sigma_{2}^{2})\;,\text{where,}\\ \;\mu_{2}\sim 𝒩(5.0\;,0.5^{2})\;,\sigma_{2}^{2}=3.0^{2}. \end{array} \end{array}$$

**Fig 5:**
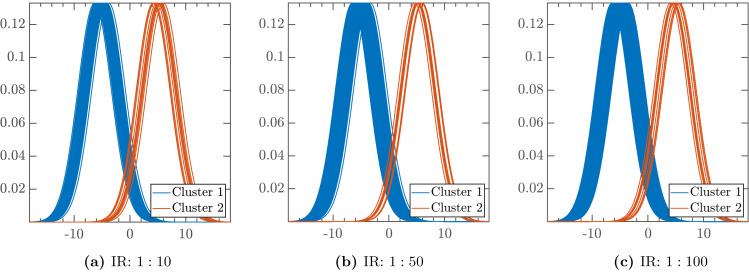
The two clusters of density functions in Example 1.

Fig 5 clearly illustrates the impact of the *IR* on the data distribution. As the *IR*s increase, such as 1:10, 1:50, and 1:100, the blue cluster becomes denser, while the red cluster remains the same size.

**Fig 6:**
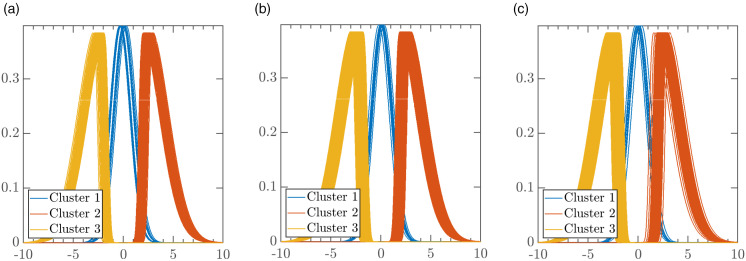
The three clusters of density functions in Example 2.

**Example 2.** In this example, we extend **Example 1** to three clusters, incorporating a more complex skew-normal distribution from http://azzalini.stat.unipd.it/SN/, introducing greater disparity among cluster distributions. The pdf of a skew-normal distribution is defined as


$$\begin{array}{c} f(x\;|\;\xi \;,\sigma \;,\lambda )=\frac{2}{\lambda }\phi \left(\frac{x-\xi }{\sigma }\right)\Phi \left(\lambda \frac{x-\xi }{\sigma }\right), \end{array}$$

where ϕ(·) and Φ(·) represent the standard normal pdf and distribution function, respectively. The ξ is the location parameter (mean), σ is the scale parameter (standard deviation), and λ controls the skewness of the distribution. The pdfs of these clusters are illustrated in Fig 6, where different *IR*s are examined: (a) 1:10:10, (b) 1:50:50, and (c) 1:100:100.

In our framework, the location parameters $\xi_{i}$ are sampled from normal distributions 𝒩(0,0.2), 𝒩(2,0.2) and 𝒩(−2,0.2) with cluster 1, 2 and 3, respectively. The scale parameters are set as $\sigma_{i}=[0,10,-10]$ while the skewness parameters are defined as $\lambda_{i}=[1,2,2]$ introducing asymmetric distributions across clusters.

**Example 3.** We simulate an imbalanced image dataset and a priori define two clusters as the colour differences of two texture images. Two original images (200 × 200 pixels, 256 grey levels) have different background intensities, including *D*83 and *D*102. These images were sourced from the Brodatz Texture database [49] at https://multibandtexture.recherche.usherbrooke.ca/original_brodatz.html and were randomly cropped and uniformly distributed into 64 × 64 images. The images are then assembled into an imbalanced dataset with *IR*s ranging from 1 to 100.

The image is extracted using a non-parametric density estimation method based on the grey distribution, as demonstrated in [25,26]. Specifically, the mapping ℑ from an image $Img\in {\mathbb{R}}^{m\times n}$ shifts into density function are shown by


$$\begin{array}{c} ℑ\;:\;{\mathbb{R}}^{m\times n}\to \hat{f} \end{array}$$

$$\begin{array}{c} Img\mapsto {\hat{f}}_{I}(x)=\frac{1}{N\cdot h}\sum_{i=1}^{N}K\left(\frac{x-I_{i}}{h}\right), \end{array}$$

where $I=\mathrm{vec}(Img)\in {\mathbb{R}}^{N}$ is the flattened vector of pixel intensities, N=m×n. In this paper, we use a Gaussian kernel function $K(t)=\frac{1}{\sqrt{2\pi }}\exp\left(-\frac{t^{2}}{2}\right)$ implemented via ksdensity(), and the bandwidth is selected according to Scott’s rule [50], $h=1.06\cdot \sigma (I)\cdot N^{-1/5}$, with σ(I) denoting the standard deviation of *I*.

**Fig 7:**
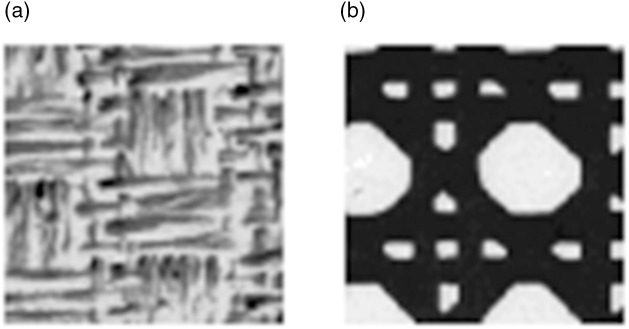
The cropped images from the Brodatz dataset.

**Fig 8:**
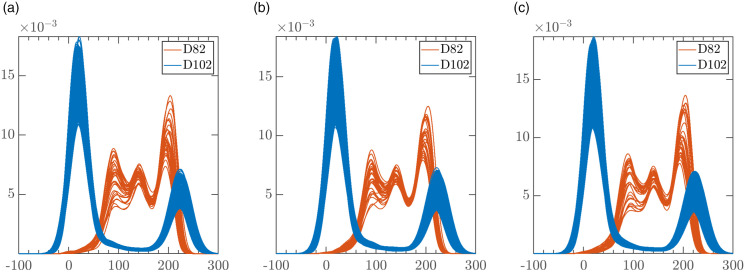
The two clusters of density functions in Example 3.

Fig 7 illustrates a cropped image and its extracted density functions. Upon reviewing Fig 8, we notice a lack of discernible differences and a significant risk of imbalance within the dataset. We perceive and divide the image dataset’s contents into two clusters based on their a priori nature (*D*83 and *D*102). The current challenge is to categorise images into their respective clusters swiftly.

The three examples provided are low-dimensional and focus on the behaviour of BKIFF with imbalanced data; thus, the number of BKIFF clusters is set to match the number of simulated clusters.

**Fig 9:**
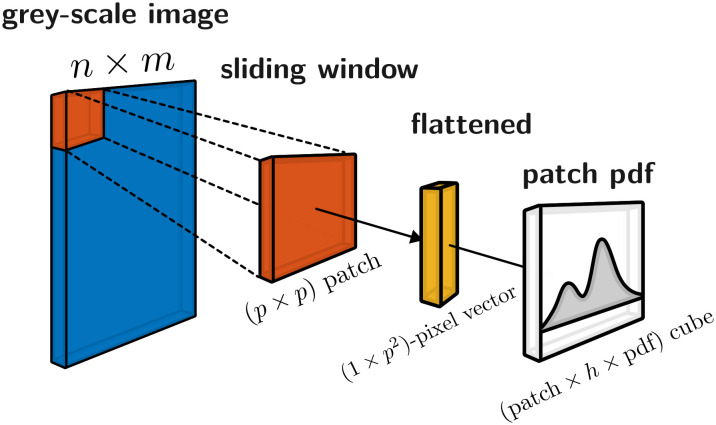
Extract patch sliding for image into pdf.

**Fig 10:**
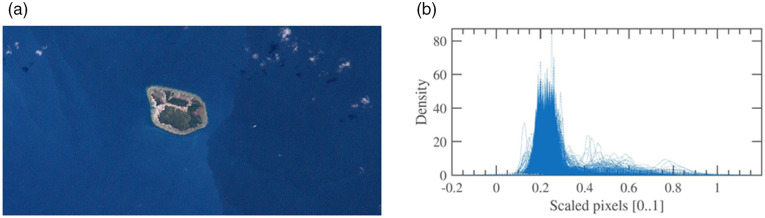
The real application image and its patches of pdfs.

**Application.** The Landsat-8 scene (512 × 256 pixels) covering Yam Island presents an extreme marine-class imbalance source from https://oceancolor.gsfc.nasa.gov/. Shallow reef flats, sandy cays, and island vegetation patches are heavily outnumbered by deep-water pixels. This severe skew demands a clustering algorithm that is robust to such imbalanced regimes. The segmentation method is also unique. We first convert the grey-scale image into a raster-scan of non-overlapping (*p* × *p*) patches, scanning from left to right and top to bottom. Each patch is now flattened into a (1 × *p*^2^)-pixel vector, its intensity density function is estimated via ksdensity(), and the resulting (patch×h×pdf) cube on 3-D is stored for patch clustering. This patch-wise strategy retains locally homogeneous spectral signatures and prevents global moderation from blurring colour boundaries. Here, we set the patch size equal to 4. Besides, the BKIFF use *m* = 2.0, c=2÷10, and *h* = 0.01 (uniform divided of dx) for this application. Fig 9 describes the process of creating a patch pdf and Fig 10 illustrates the original image and its extracted pdf data.

In conclusion, the Table 3 presents the input configurations for the three numerical examples.

**Table 3:** Configuration of numerical examples.

Configuration	Example 1	Example 2	Example 3	Application
Data Source	Synthetic	Synthetic	Brodatz	Yam Island
Dataset Type	Functional data	Functional data	Image	Image
Distribution Type	Gaussian dist.	Gaussian dist.	Kernel dist.	Kernel dist.
*IR*	1÷100	1÷100	1÷100	–
Minority Numbers	10 functions	10 functions	30 functions	–
Majority Numbers	10 × *IR*	10 × *IR*	30 × *IR*	–
Cluster number	2	3	2	2÷10
Description	Fig 5	Fig 6	Fig 8	Fig 10

### 4.1 Experiment setup

For a fair comparison between the proposed algorithms, we introduce the baseline algorithms including *k*-means (*K*MEAN [30]), fuzzy clustering (FCM CWD [51], FCM-${ℒ}_{1}$ [1]), self-updating process (SUP [29]), and Dinh Pham-Toan’s method (2025) [28] specifically for analysing density functions. These baseline algorithms are contingent on the specific datasets used, highly novel, and have been proven effective by stakeholders. Table 4 describes the proposed method’s parameters and baselines.

**Table 4:** Configuration of clustering algorithms for comparative analysis.

Method	Description	Input Parameters
FCF-${ℒ}_{1}$ [1]	based on the traditional fuzzy *c*-means with the ${ℒ}_{1}$-distance between two density functions	$maxIter=500\;,m=2\;,\varepsilon =1\times 10^{-6}$
FCF-*CWD* [51]	based on the traditional fuzzy *c*-means with the cluster-width distance between two density functions	$maxIter=500\;,m=2\;,\varepsilon =1\times 10^{-6}$
*K*MEANS [30]	based on the traditional *k*-means with the ${ℒ}_{1}$-distance between two density functions	*maxIter* = 500
SUP (*Self-Updating Process*) [29]	instruct an automatic clustering founded on the pair ${ℒ}_{1}$-distance matrix of density functions	$maxIter=500\;,\epsilon =1\times 10^{-6}$
Dinh Pham-Toan’s method [28]	instruct an automatic clustering founded on the pair Kullback–Leibler divergence of density functions	$maxIter=500\;,\epsilon =1\times 10^{-6}$
BKIFF (Proposed)	–	$maxIter=500\;,m=2\;,\varepsilon =1\times 10^{-6}$

Our analysis examines the efficiency of the BKO initialisation, the role of the Hellinger distance, the performance of the BKIFF algorithm, and the behaviour of its fuzziness component. When evaluating the BKO initialisation specifically, average performance is compared using the nonparametric Wilcoxon signed-rank test. For all other comparative analyses involving multiple methods, the Friedman test is applied. These tests have a significance level α-value of 0.1%.

We report the experiments by multiple internal and external criteria as described, including *ARI*, *NMI*, *Silhouette* score, and *Dunn* Index (See more at Some validity measure indexes for clustering solution subsection). Moreover, the computational time consumption is also presented.

### 4.2 Simulation strategy

It is important to note that all algorithms are executed 10 times independently using the Monte Carlo method to ensure stable seed reproducibility from 1 to 10, and the median and interquartile range (IQR) are calculated. Furthermore, all computations are carried out using Octave (or MATLAB) on an Intel${\;}^{®}$ Core^TM^ i5-11400H @ 2.70GHz with 16.0 GB main memory. Ultimately, the performance results will be presented, along with the main conclusions drawn from the study. Finally, several key conclusions are drawn from the results obtained. Algorithms, including the proposed baseline, are compatible and can be run in programming software using Octave code, which is publicly available at https://doi.org/10.6084/m9.figshare.30600539.v4.

### 4.3 Sensitivity analysis setup

The author conducts a sensitivity analysis to evaluate the stability and reliability of the BKIFF algorithm across various parameter settings for the two-phase BKO and IFF. On the on hand, we conduct a sensitivity analysis for BKO-based initialisation across three parameter groups including *IR* = {20,50,80,100}, population sizes *pop* = {20,30,50,80,100}, and iteration counts $T_{\max}=\{50\;,100\;,200\}$. Sample indices bounded the search space, and the objective function maximised the pairwise separation among selected initial prototypes, computed via ${ℋ}^{2}$-distance. We recorded both the *F*_*best*_ value and the execution time (in seconds). On the other hand, we test the four groups of factors of IFF clustering algorithm, including configurations *IR*={20,50,80,100}, fuzziness coefficient *m*={1.1,1.5,2.0,2.5,3.0,5.0,10}, the number of clusters *c*={2,3,4,5}, and the type of distance metric $\left\{{ℋ}^{2}\;,{ℒ}_{1}\;,{ℒ}_{2}\;,CWD\right\}$. Evaluation metrics included *ARI* and *NMI* for external clustering fit, as well as *Silhouette* and *Dunn* indices for internal clustering quality. Moreover, we record the number of iterations required for convergence and the execution time. Subsequently, the results were analysed using a multi-factor ANOVA and the Morris [52] screening methods.

## 5 Main results

We present the results by four factors, including intrinsic efficiency of the differential initiation method, distances, fuzziers, and the extrinsic efficiency of the proposed method. The sensitivity analyses are supported by Supplementary Information B.

### 5.1 Effectiveness of initialisation method

This section evaluates the binary results when comparing BKO-based (BKIFF) and random initialisation (IFF). Overall, the choice of pre-processing method significantly impacts computational efficiency and convergence speed. The experimental results demonstrate that integrating the BKO mechanism into the initialisation stage does not affect clustering accuracy; however, it yields substantial and statistically significant gains in computational efficiency and robustness, particularly under severe class imbalance.

As reported in Tables 5, 6, and 7, Example 1 exhibits complete invariance in clustering quality across all *IR* ranging from 20 to 100. For both IFF and BKIFF, the median values of *ARI* and *NMI* remain at 1.0 (IQR, 0), while the *Dunn* index decreases identically from 5.1 at *IR* = 20 to 3.9 at *IR* = 100. Similarly, *Silhouette* values remain stable at approximately 0.99 for both methods. Consistently, no statistically significant differences were observed between IFF and BKIFF for any clustering metric (all *p* = 1.0).

**Table 5:** Comparison of with/without BKO in varying *IR* (Example 1).

Metric	IR	IFF	BKIFF	Wilcoxon	*p*-value
ARI	20	1.000 (0.000)	1.000 (0.000)	0	1.000
	50	1.000 (0.000)	1.000 (0.000)	0	1.000
	80	1.000 (0.000)	1.000 (0.000)	0	1.000
	100	1.000 (0.000)	1.000 (0.000)	0	1.000
NMI	20	1.000 (0.000)	1.000 (0.000)	0	1.000
	50	1.000 (0.000)	1.000 (0.000)	0	1.000
	80	1.000 (0.000)	1.000 (0.000)	0	1.000
	100	1.000 (0.000)	1.000 (0.000)	0	1.000
Dunn	20	5.093 (0.000)	5.093 (0.000)	0	1.000
	50	4.571 (0.000)	4.571 (0.000)	0	1.000
	80	3.691 (0.000)	3.691 (0.000)	0	1.000
	100	3.876 (0.000)	3.876 (0.000)	0	1.000
Silhouette	20	0.990 (0.000)	0.990 (0.000)	0	1.000
	50	0.991 (0.000)	0.991 (0.000)	0	1.000
	80	0.991 (0.000)	0.991 (0.000)	0	1.000
	100	0.990 (0.000)	0.990 (0.000)	0	1.000
Comp. Time	20	**0.007 (0.002)**	0.005 (0.001)	12	<0.001
	50	**0.022 (0.004)**	0.012 (0.003)	0	<0.001
	80	**0.059 (0.016)**	0.022 (0.005)	0	<0.001
	100	**0.129 (0.059)**	0.031 (0.006)	0	<0.001
Iterations	20	**11 (2.0)**	7.0 (1.0)	5	<0.001
	50	**15 (4.0)**	9.0 (2.0)	0	<0.001
	80	**26 (6.0)**	10 (2.0)	0	<0.001
	100	**48 (19.0)**	11 (2.0)	0	<0.001

**Table 6:** Comparison of with/without BKO in varying *IR* (Example 2).

Metric	IR	IFF	BKIFF	Wilcoxon	*p*-value
ARI	20	1.000 (0.000)	1.000 (0.000)	0	1.000
	50	1.000 (0.000)	1.000 (0.000)	0	1.000
	80	0.793 (0.012)	**1.000 (0.000)**	325	<0.001
	100	0.777 (0.010)	**1.000 (0.000)**	406	< 0.001
NMI	20	1.000 (0.000)	1.000 (0.000)	0	1.000
	50	1.000 (0.000)	1.000 (0.000)	0	1.000
	80	0.812 (0.006)	**1.000 (0.000)**	325	< 0.001
	100	0.805 (0.005)	**1.000 (0.000)**	406	< 0.001
Dunn	20	2.968 (0.000)	2.968 (0.000)	0	1.000
	50	2.596 (0.000)	2.596 (0.000)	0	1.000
	80	0.000 (0.000)	**2.464 (0.000)**	325	< 0.001
	100	0.000 (0.001)	**2.470 (0.000)**	406	< 0.001
Silhouette	20	0.976 (0.000)	0.976 (0.000)	0	1.000
	50	0.978 (0.000)	0.978 (0.000)	0	1.000
	80	0.759 (0.016)	**0.978 (0.000)**	325	< 0.001
	100	0.753 (0.017)	**0.979 (0.000)**	406	< 0.001
Time	20	**0.028 (0.012)**	0.014 (0.002)	0	< 0.001
	50	**0.108 (0.023)**	0.040 (0.003)	1	< 0.001
	80	**0.290 (0.094)**	0.084 (0.006)	1	< 0.001
	100	**0.294 (0.051)**	0.120 (0.010)	1	< 0.001
iter	20	**17 (9.0)**	9.0 (0.0)	0	< 0.001
	50	**36 (9.0)**	12.5 (1.0)	1	< 0.001
	80	**62 (19.0)**	17.0 (1.0)	1	< 0.001
	100	**50 (10.0)**	19.0 (1.0)	1	< 0.001

**Table 7:** Comparison of with/without BKO in varying *IR* (Example 3).

Metric	IR	IFF	BKIFF	Wilcoxon	*p*-value
ARI	20	1.000 (0.000)	1.000 (0.000)	0	1.000
	50	0.010 (0.000)	**1.000 (0.000)**	406	< 0.001
	80	0.005 (0.000)	**1.000 (0.000)**	435	< 0.001
	100	0.004 (0.000)	**1.000 (0.000)**	465	< 0.001
NMI	20	1.000 (0.000)	1.000 (0.000)	0	1.000
	50	0.041 (0.000)	**1.000 (0.000)**	406	< 0.001
	80	0.026 (0.000)	**1.000 (0.000)**	435	< 0.001
	100	0.021 (0.000)	**1.000 (0.000)**	465	< 0.001
Dunn	20	6.308 (0.000)	6.308 (0.000)	0	1.000
	50	0.000 (0.000)	**7.663 (0.000)**	406	< 0.001
	80	0.000 (0.000)	**6.684 (0.000)**	435	< 0.001
	100	0.000 (0.000)	**5.800 (0.000)**	465	< 0.001
Silhouette	20	0.987 (0.000)	0.987 (0.000)	0	1.000
	50	0.202 (0.000)	**0.987 (0.000)**	406	< 0.001
	80	0.236 (0.000)	**0.987 (0.000)**	435	< 0.001
	100	0.264 (0.000)	**0.987 (0.000)**	465	< 0.001
Comp. Time	20	**0.022 (0.005)**	0.010 (0.002)	4	< 0.001
	50	**0.106 (0.015)**	0.031 (0.004)	0	< 0.001
	80	**0.152 (0.027)**	0.062 (0.006)	1	< 0.001
	100	**2.044 (3.805)**	0.090 (0.010)	0	< 0.001
Iterations	20	**13 (3.0)**	5.0 (1.0)	2	< 0.001
	50	**27 (4.0)**	7.5 (1.0)	0	< 0.001
	80	**24 (4.0)**	9.0 (1.0)	0	< 0.001
	100	**263 (477)**	11 (1.0)	0	< 0.001

In contrast, empirical studies in clustering research by [30,31] have demonstrated that poor initialisation can lead to fuzzy clustering algorithms becoming trapped in local optima, thereby increasing iteration counts. Pronounced differences, therefore, emerge when computational behaviour is considered. In Example 1, the median runtime of IFF increases from 0.01 s at *IR*  = 20 to 0.13 s at *IR* = 100, accompanied by a widening *IQR* that reaches 0.06 s at the highest imbalance level. BKIFF, by comparison, exhibits a markedly slower growth in runtime, increasing only from 0.01 s to 0.03 s over the same range, corresponding to a reduction of approximately 50%−70%. A similar trend is observed in iteration counts. While IFF requires a median of 11 iterations at *IR* = 20, rising sharply to 48 iterations at *IR* = 100 (IQR, 19), BKIFF converges consistently within 7÷11 iterations (IQR, 1÷2). These efficiency gains are statistically significant for all IR (*p* < 0.001). The stabilising effect of BKO becomes more evident in Examples 2 and 3. In Example 2, both methods achieve perfect clustering at *IR* = 20 and *IR* = 50; however, IFF performance deteriorates substantially at higher *IR*, with median *ARI* values decreasing to 0.8 at *IR* = 80 and *IR* = 100. In contrast, BKIFF preserves an *ARI* of 1.0 (IQR, 0) across all replications. This pattern is consistently reflected in *NMI*, *Dunn*, and *Silhouette* indices, and the corresponding Wilcoxon statistics yield *p* < 0.001. An even more pronounced divergence is observed in Example 3. At *IR* = 100, IFF records a median *ARI* of 0.004 and requires 263 iterations (IQR, 477), indicating volatile convergence behaviour. Conversely, BKIFF achieves perfect clustering (*ARI* = 1.0) and converges within a median of 11 iterations (IQR, 1). In terms of runtime, IFF reaches a median of 2.04 s, whereas BKIFF completes the optimisation in approximately 0.09 s. All observed differences in computational metrics are statistically significant (*p* < 0.001).

### 5.2 Effectiveness of Hellinger distance

The comparative analysis across distance measures with the ${ℋ}^{2}$-distance consistently outperforms the remaining metrics, followed by the ${ℒ}_{2}$-distance, across all three experimental settings. As reported in Tables 8, 9, and 10, the ${ℋ}^{2}$-based BKIFF achieves perfect clustering quality in all examples, maintaining *ARI* = *NMI* = 1.0 (IQR, 0). In addition, it yields high cluster separation, as reflected by *Dunn* indices exceeding 2.4 in Examples 1 and 2 and remaining above 5.8 in Example 3, along with *Silhouette* values consistently close to 1.0.

**Table 8:** Comparison of different distances with varying *IR* (Example 1).

Metric	IR	${ℒ}_{1}$-distance	CWD	${ℒ}_{2}$-distance	${ℋ}^{2}$-distance	Friedman *p*-value
*ARI*	20	0.249 (0.000)	0.249 (0.000)	1.000 (0.000)	1.000 (0.000)	< 0.001
	50	0.008 (0.000)	0.008 (0.000)	1.000 (0.000)	1.000 (0.000)	< 0.001
	80	0.002 (0.000)	0.002 (0.000)	1.000 (0.000)	1.000 (0.000)	< 0.001
	100	0.001 (0.000)	0.001 (0.000)	1.000 (0.000)	1.000 (0.000)	< 0.001
*NMI*	20	0.225 (0.000)	0.225 (0.000)	1.000 (0.000)	1.000 (0.000)	< 0.001
	50	0.039 (0.000)	0.039 (0.000)	1.000 (0.000)	1.000 (0.000)	< 0.001
	80	0.023 (0.000)	0.023 (0.000)	1.000 (0.000)	1.000 (0.000)	< 0.001
	100	0.019 (0.000)	0.019 (0.000)	1.000 (0.000)	1.000 (0.000)	< 0.001
*Dunn Index*	20	0.000 (0.000)	0.000 (0.000)	5.093 (0.000)	5.093 (0.000)	< 0.001
	50	0.000 (0.000)	0.000 (0.000)	4.571 (0.000)	4.571 (0.000)	< 0.001
	80	0.000 (0.000)	0.000 (0.000)	3.691 (0.000)	3.691 (0.000)	< 0.001
	100	0.000 (0.000)	0.000 (0.000)	3.876 (0.000)	3.876 (0.000)	< 0.001
*Silhouette*	20	0.638 (0.000)	0.638 (0.000)	0.990 (0.000)	0.990 (0.000)	< 0.001
	50	0.213 (0.000)	0.213 (0.000)	0.991 (0.000)	0.991 (0.000)	< 0.001
	80	0.250 (0.000)	0.250 (0.000)	0.991 (0.000)	0.991 (0.000)	< 0.001
	100	0.277 (0.000)	0.278 (0.000)	0.990 (0.000)	0.990 (0.000)	< 0.001
Comp. Time	20	0.034 (0.001)	0.040 (0.001)	0.002 (0.000)	0.005 (0.000)	< 0.001
	50	0.020 (0.002)	0.025 (0.004)	0.006 (0.002)	0.013 (0.004)	< 0.001
	80	0.024 (0.003)	0.029 (0.005)	0.011 (0.004)	0.020 (0.004)	< 0.001
	100	0.523 (0.045)	0.649 (0.052)	0.015 (0.002)	0.029 (0.006)	< 0.001
Iterations	20	167 (8.0)	166 (8.0)	6.0 (2.0)	7.0 (1.0)	< 0.001
	50	32 (4.0)	32 (4.0)	10 (2.0)	9.0 (2.0)	< 0.001
	80	27 (3.0)	27 (3.0)	11 (3.0)	10 (2.0)	< 0.001
	100	500 (0.0)	500 (0.0)	14 (3.0)	11 (2.0)	< 0.001

**Table 9:** Comparison of different distances with varying *IR* (Example 2).

Metric	IR	${ℒ}_{1}$-distance	CWD	${ℒ}_{2}$-distance	${ℋ}^{2}$-distance	Friedman *p*-value
*ARI*	20	0.765 (0.000)	0.762 (0.002)	1.000 (0.000)	1.000 (0.000)	< 0.001
	50	0.762 (0.000)	0.762 (0.000)	1.000 (0.000)	1.000 (0.000)	< 0.001
	80	0.755 (0.000)	0.754 (0.000)	0.746 (0.000)	1.000 (0.000)	< 0.001
	100	0.755 (0.000)	0.755 (0.000)	0.748 (0.000)	1.000 (0.000)	< 0.001
*NMI*	20	0.787 (0.000)	0.786 (0.001)	1.000 (0.000)	1.000 (0.000)	< 0.001
	50	0.793 (0.000)	0.793 (0.000)	1.000 (0.000)	1.000 (0.000)	< 0.001
	80	0.793 (0.000)	0.793 (0.000)	0.789 (0.000)	1.000 (0.000)	< 0.001
	100	0.794 (0.000)	0.794 (0.000)	0.791 (0.000)	1.000 (0.000)	< 0.001
*DunnIndex*	20	0.000 (0.000)	0.000 (0.000)	2.968 (0.000)	2.968 (0.000)	< 0.001
	50	0.000 (0.000)	0.000 (0.000)	2.596 (0.000)	2.596 (0.000)	< 0.001
	80	0.000 (0.000)	0.000 (0.000)	0.000 (0.000)	2.464 (0.000)	< 0.001
	100	0.000 (0.000)	0.000 (0.000)	0.000 (0.000)	2.470 (0.000)	< 0.001
*Silhouette*	20	0.664 (0.000)	0.657 (0.003)	0.976 (0.000)	0.976 (0.000)	< 0.001
	50	0.695 (0.000)	0.695 (0.000)	0.978 (0.000)	0.978 (0.000)	< 0.001
	80	0.717 (0.003)	0.716 (0.000)	0.689 (0.001)	0.978 (0.000)	< 0.001
	100	0.733 (0.000)	0.733 (0.000)	0.717 (0.000)	0.979 (0.000)	< 0.001
Comp. Time	20	0.041 (0.006)	0.279 (0.034)	0.006 (0.001)	0.014 (0.002)	< 0.001
	50	0.076 (0.018)	0.079 (0.017)	0.021 (0.004)	0.041 (0.006)	< 0.001
	80	0.100 (0.022)	0.110 (0.013)	0.085 (0.026)	0.094 (0.023)	< 0.001
	100	0.110 (0.027)	0.125 (0.032)	0.083 (0.021)	0.130 (0.014)	< 0.001
Iterations	20	76 (0.0)	500 (0.0)	10 (0.0)	9.0 (0.0)	< 0.001
	50	57 (5.0)	56 (5.0)	17 (2.0)	13 (1.0)	< 0.001
	80	52 (3.0)	51 (3.0)	41 (6.0)	17 (1.0)	< 0.001
	100	46 (3.0)	46 (2.0)	37 (6.0)	19 (1.0)	< 0.001

**Table 10:** Comparison of different distances with varying *IR* (Example 3).

Metric	IR	${ℒ}_{1}$-distance	CWD	${ℒ}_{2}$-distance	${ℋ}^{2}$-distance	Friedman *p*-value
*ARI*	20	0.000 (0.000)	0.000 (0.000)	1.000 (0.000)	1.000 (0.000)	< 0.001
	50	0.000 (0.000)	0.000 (0.000)	1.000 (0.000)	1.000 (0.000)	< 0.001
	80	0.002 (0.002)	0.002 (0.002)	1.000 (0.000)	1.000 (0.000)	< 0.001
	100	0.000 (0.000)	0.000 (0.000)	1.000 (0.995)	1.000 (0.000)	< 0.001
*NMI*	20	0.000 (0.000)	0.000 (0.000)	1.000 (0.000)	1.000 (0.000)	< 0.001
	50	0.000 (0.000)	0.000 (0.000)	1.000 (0.000)	1.000 (0.000)	< 0.001
	80	0.024 (0.000)	0.024 (0.000)	1.000 (0.000)	1.000 (0.000)	< 0.001
	100	0.000 (0.000)	0.000 (0.000)	1.000 (0.978)	1.000 (0.000)	< 0.001
*DunnIndex*	20	0.000 (0.000)	0.000 (0.000)	6.308 (0.000)	6.308 (0.000)	< 0.001
	50	0.000 (0.000)	0.000 (0.000)	7.663 (0.000)	7.663 (0.000)	< 0.001
	80	0.000 (0.000)	0.000 (0.000)	6.684 (0.000)	6.684 (0.000)	< 0.001
	100	0.000 (0.000)	0.000 (0.000)	5.800 (5.800)	5.800 (0.000)	< 0.001
*Silhouette*	20	0.000 (0.000)	0.000 (0.000)	0.987 (0.000)	0.987 (0.000)	< 0.001
	50	0.000 (0.000)	0.000 (0.000)	0.987 (0.000)	0.987 (0.000)	< 0.001
	80	0.206 (0.206)	0.206 (0.206)	0.987 (0.000)	0.987 (0.000)	< 0.001
	100	0.000 (0.000)	0.000 (0.000)	0.987 (0.712)	0.987 (0.000)	< 0.001
Comp. Time	20	0.051 (0.005)	0.063 (0.005)	0.006 (0.001)	0.010 (0.002)	< 0.001
	50	0.381 (0.157)	0.523 (0.178)	0.020 (0.011)	0.037 (0.017)	< 0.001
	80	1.664 (0.398)	2.356 (0.024)	0.057 (0.012)	0.091 (0.011)	< 0.001
	100	2.429 (0.045)	2.855 (0.063)	0.099 (0.022)	0.134 (0.021)	< 0.001
Iterations	20	72 (2.0)	72 (3.0)	6.0 (1.0)	5.0 (1.0)	< 0.001
	50	195 (1.0)	199 (1.0)	9.0 (2.0)	7.5 (1.0)	< 0.001
	80	404 (98.0)	500 (0.0)	1200 (3.0)	9.0 (1.0)	< 0.001
	100	500 (0.0)	500 (0.0)	20 (5.0)	11 (1.0)	< 0.001

By contrast, both the ${ℒ}_{1}$-distance and the CWD metric exhibit systematic performance degradation across all imbalance levels. In Example 1, these distances produce *ARI* and *NMI* values below 0.01 once *IR* = 20 and *IR* = 50, with *Dunn* indices collapsing to 0 and *Silhouette* values remaining below 0.30. Similar behaviour is observed in Examples 2 and 3, where clustering quality metrics for ${ℒ}_{1}$ and CWD approach or equal 0 irrespective of imbalance severity, indicating an inability to recover meaningful cluster structures. The ${ℒ}_{2}$-distance shows competitive performance in Example 1 and moderate robustness in Example 2; however, its effectiveness deteriorates under the more challenging density configurations of Example 3, where increased variability is observed in both quality and convergence behaviour. In all cases, the observed differences among distance measures are statistically significant (Friedman tests, *p* < 0.001)

The superiority of the ${ℋ}^{2}$-distance is further reinforced by its computational efficiency and stability. Across all three examples, ${ℋ}^{2}$ and ${ℒ}_{2}$ consistently deliver the lowest runtimes and iteration counts. Specifically, their execution times remain within a few milliseconds in Example 1, below 0.13 s in Example 2, and between 0.01 and 0.10 s in Example 3. Correspondingly, convergence is achieved within 6–14 iterations in Example 1, 9–19 iterations in Example 2, and 5–11 iterations in Example 3, with minimal IQR values indicating stable optimisation behaviour. In sharp contrast, the ${ℒ}_{1}$- and CWD distances require substantially higher computational effort. In particular, under high imbalance, these metrics demand tens to hundreds of optimisation steps and frequently reach the maximum iteration cap of 500. Their runtimes exceed 0.5 s in moderate cases and escalate to approximately 2–3 s in the most challenging scenarios, accompanied by large dispersion across replications. The Friedman test results for runtime and iteration counts consistently report *p* < 0.001, confirming statistically significant differences in computational cost across distance measures.

In conclusion, this comparison highlights that inappropriate distance measures not only compromise clustering quality but also lead to extremely long and unstable iterations. ${ℋ}^{2}$-distance performed best among the compared methods that keeps optimal quality once *IR* is over 80, while ${ℒ}_{2}$, previously robust in Example 1, begins to waver, and ${ℒ}_{1}$-distance or CWD completely collapse. This empirical demonstration provides a practical guideline for selecting the distance based on *IR* rather than a default.

### 5.3 Effectiveness of Fuzziness

**Fig 11:**
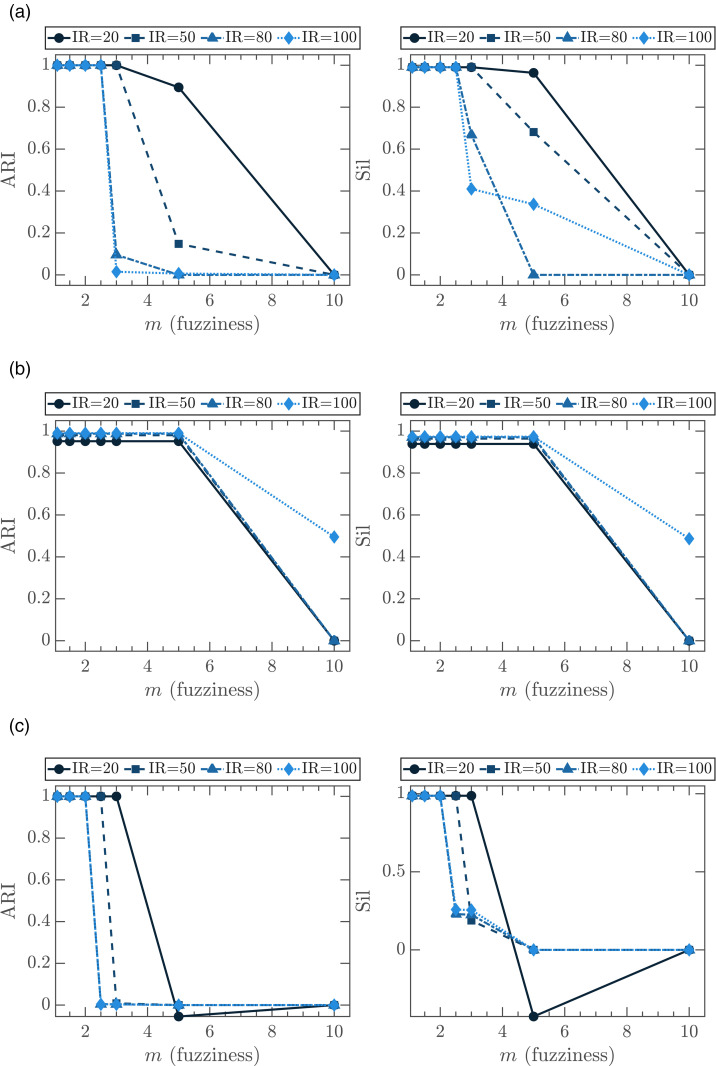
The inverstigrating of fuzziness of BKIFF on three examples.

Three pairs of curves in three Fig 11(a), 11(b), and 11(c) with the same horizontal axis m∈{1.1,1.5,2.0,2.5,5.0,10.0} and the vertical axis of quality. In Fig 11(a) the four overlapping lines are horizontal at ARI=0.98÷1.0, while in (b) the two IR=20÷50 curves remain high, the two *IR* = 80, and 100, curves plummet from *m* = 5.0, and in (c) only the *IR* = 20 curve remains high, while the *IR* > 50 curves fall close to 0.0 right from low *m*.

The experimental results reveal a pronounced dependency of clustering performance on the fuzziness parameter *m* under varying *IR*. In the proposed algorithm, high-quality clustering is maintained for small *m* values, regardless of *IR*. As *m* increases, performance degradation occurs more abruptly for higher *IR*, yet the proposed method exhibits markedly slower deterioration compared to typical FCM behaviour reported in earlier studies [1]. Specifically, for low *IR* = 20, or 50, both *ARI* and *Silhouette* remain near-optimal until *m* = 5.0, after which the scores drop sharply. For higher *IR* > 80, conventional fuzzy clustering, as documented in prior works [30,53], tends to lose cluster separability at much smaller *m* = 2.0, often leading to near-random assignments. In contrast, the proposed method delays this collapse, maintaining competitive clustering quality over a wider range of *m*.

### 5.4 Effectiveness of BKIFF

The comparative evaluation across all three examples (Tables 11–13) shows that BKIFF consistently maintains good clustering performance across the examined range of imbalance ratios. In all settings, BKIFF attains ceiling-level scores with *ARI* = *NMI* = 1.0, maintains high *Dunn* indices, and achieves *Silhouette* values close to or equal to 1.0, regardless of imbalance severity. This behaviour contrasts sharply with that of the remaining competitors, whose performance deteriorates as the imbalance increases.

**Table 11:** Comparison of state-of-the-art methods with varying *IR* (Example 1).

Metric	IR	FCM CWD	FCM-${ℒ}_{1}$	*K*MEAN	Self-Updating	BKIFF	Dinh Pham-Toan (2025) [28]	Friedman *p*-value
*ARI*	20	1.00 (0.00)	1.00 (0.00)	1.00 (0.00)	1.00 (0.00)	1.00 (0.00)	1.00 (0.00)	1.00
	50	0.01 (0.00)	0.01 (0.00)	1.00 (0.00)	1.00 (0.00)	1.00 (0.00)	1.00 (0.00)	< 0.001
	80	< 0.01 (0.00)	0.00 (0.00)	0.02 (0.00)	0.95 (0.00)	1.00 (0.00)	1.00 (0.00)	< 0.001
	100	< 0.01 (0.00)	0.00 (0.00)	0.01 (0.00)	1.00 (0.00)	1.00 (0.00)	1.00 (0.00)	< 0.001
*NMI*	20	1.00 (0.00)	1.00 (0.00)	1.00 (0.00)	1.00 (0.00)	1.00 (0.00)	1.00 (0.00)	1.00
	50	0.04 (0.00)	0.04 (0.00)	1.00 (0.00)	1.00 (0.00)	1.00 (0.00)	1.00 (0.00)	< 0.001
	80	0.02 (0.00)	0.02 (0.00)	0.03 (0.00)	0.00 (0.00)	1.00 (0.00)	1.00 (0.00)	< 0.001
	100	0.02 (0.00)	0.02 (0.00)	0.03 (0.00)	0.00 (0.00)	1.00 (0.00)	1.00 (0.00)	< 0.001
*Dunn* Index	20	5.09 (0.00)	5.09 (0.00)	5.09 (0.00)	99551 (0.00)	5.09 (0.00)	90991 (0.00)	< 0.001
	50	0.00 (0.00)	0.00 (0.00)	4.57 (0.00)	88459 (0.00)	4.57 (0.00)	83289 (0.00)	< 0.001
	80	0.00 (0.00)	0.00 (0.00)	0.00 (0.00)	1532 (0.00)	3.69 (0.00)	85170 (0.00)	< 0.001
	100	0.00 (0.00)	0.00 (0.00)	0.00 (0.00)	5269 (0.00)	3.88 (0.00)	82856 (0.00)	< 0.001
*Silhouette*	20	0.99 (0.00)	0.99 (0.00)	0.99 (0.00)	1.00 (0.00)	0.99 (0.00)	1.00 (0.00)	1.00
	50	0.22 (0.00)	0.22 (0.00)	0.99 (0.00)	1.00 (0.00)	0.99 (0.00)	1.00 (0.00)	< 0.001
	80	0.25 (0.00)	0.25 (0.00)	0.37 (0.00)	1.00 (0.00)	0.99 (0.00)	1.00 (0.00)	< 0.001
	100	0.28 (0.00)	0.28 (0.00)	0.40 (0.00)	1.00 (0.00)	0.99 (0.00)	1.00 (0.00)	< 0.001
Comp. Time	20	0.02 (0.01)	0.02 (0.01)	< 0.01 (0.00)	0.15 (0.00)	0.01 (0.00)	2.26 (0.02)	< 0.001
	50	0.01 (0.00)	0.01 (0.00)	0.01 (0.00)	1.08 (0.09)	0.01 (0.00)	12.97 (0.06)	< 0.001
	80	0.01 (0.00)	0.01 (0.00)	0.01 (0.01)	2.75 (0.22)	0.02 (0.00)	33 (0.04)	< 0.001
	100	0.02 (0.00)	0.01 (0.00)	0.02 (0.01)	4.43 (0.10)	0.03 (0.00)	64 (0.08)	< 0.001
Iterations	20	66 (40.00)	66 (40.00)	4.00 (2.00)	4.00 (0.00)	8.00 (1.00)	3.00 (0.00)	< 0.001
	50	14 (4.00)	14 (4.00)	9.00 (5.00)	5.00 (0.00)	8.00 (2.00)	3.00 (0.00)	< 0.001
	80	11 (4.00)	11 (4.00)	10 (4.00)	5.00 (0.00)	11 (2.00)	3.00 (0.00)	< 0.001
	100	11 (2.00)	11 (2.00)	13 (3.00)	5.00 (0.00)	11 (2.00)	4.00 (0.00)	< 0.001

**Table 12:** Comparison of state-of-the-art methods with varying *IR* (Example 2).

Metric	IR	FCM CWD	FCM-${ℒ}_{1}$	*K*MEAN	Self-Updating	BKIFF	Dinh Pham-Toan (2025) [28]	Friedman *p*-value
*ARI*	20	0.75 (0.00)	0.75 (0.00)	1.00 (0.00)	1.00 (0.00)	1.00 (0.00)	0.00 (0.00)	< 0.001
	50	0.75 (0.01)	0.76 (0.01)	1.00 (0.00)	1.00 (0.00)	1.00 (0.00)	0.00 (0.00)	< 0.001
	80	0.75 (0.00)	0.75 (0.00)	0.76 (0.07)	1.00 (0.00)	1.00 (0.00)	0.00 (0.00)	< 0.001
	100	0.75 (0.00)	0.75 (0.00)	0.76 (0.03)	1.00 (0.00)	1.00 (0.00)	0.00 (0.00)	< 0.001
*NMI*	20	0.77 (0.00)	0.77 (0.00)	1.00 (0.00)	1.00 (0.00)	1.00 (0.00)	0.00 (0.00)	< 0.001
	50	0.79 (0.00)	0.79 (0.00)	1.00 (0.00)	1.00 (0.00)	1.00 (0.00)	0.00 (0.00)	< 0.001
	80	0.79 (0.00)	0.79 (0.00)	0.79 (0.04)	1.00 (0.00)	1.00 (0.00)	0.00 (0.00)	< 0.001
	100	0.79 (0.00)	0.79 (0.00)	0.79 (0.01)	1.00 (0.00)	1.00 (0.00)	0.00 (0.00)	< 0.001
*Dunn* Index	20	0.00 (0.00)	0.00 (0.00)	2.97 (0.00)	21 (0.00)	2.97 (0.00)	0.00 (0.00)	< 0.001
	50	0.00 (0.00)	0.00 (0.00)	2.60 (0.00)	21 (0.00)	2.60 (0.00)	0.00 (0.00)	< 0.001
	80	0.00 (0.00)	0.00 (0.00)	0.00 (0.00)	21 (0.00)	2.46 (0.00)	0.00 (0.00)	< 0.001
	100	0.00 (0.00)	0.00 (0.00)	0.00 (0.00)	21 (0.00)	2.47 (0.00)	0.00 (0.00)	< 0.001
*Silhouette*	20	0.66 (0.00)	0.66 (0.00)	0.98 (0.00)	1.00 (0.00)	0.98 (0.00)	0.00 (0.00)	< 0.001
	50	0.68 (0.03)	0.70 (0.03)	0.98 (0.00)	1.00 (0.00)	0.98 (0.00)	0.00 (0.00)	< 0.001
	80	0.76 (0.00)	0.76 (0.00)	0.76 (0.03)	1.00 (0.00)	0.98 (0.00)	0.00 (0.00)	< 0.001
	100	0.76 (0.01)	0.76 (0.01)	0.76 (0.02)	1.00 (0.00)	0.98 (0.00)	0.00 (0.00)	< 0.001
Comp. Time	20	0.04 (0.05)	0.03 (0.03)	< 0.01 (0.00)	0.47 (0.02)	0.01 (0.00)	4.37 (0.00)	< 0.001
	50	0.04 (0.02)	0.03 (0.02)	0.02 (0.01)	2.44 (0.24)	0.04 (0.01)	10 (0.00)	< 0.001
	80	0.06 (0.03)	0.05 (0.03)	0.02 (0.01)	6.95 (0.35)	0.09 (0.01)	16 (0.00)	< 0.001
	100	0.06 (0.03)	0.05 (0.03)	0.02 (0.02)	11 (0.50)	0.14 (0.02)	20 (0.00)	< 0.001
Iterations	20	60 (70.00)	63 (61.00)	4.50 (5.00)	4.00 (0.00)	9.00 (0.00)	500 (0.00)	< 0.001
	50	23 (14.00)	25 (16.00)	9.50 (9.00)	3.00 (0.00)	13 (1.00)	500 (0.00)	< 0.001
	80	26 (14.00)	25 (15.00)	6.00 (5.00)	3.00 (0.00)	17 (2.00)	500 (0.00)	< 0.001
	100	21 (11.00)	22 (12.00)	6.00 (6.00)	3.00 (0.00)	20 (2.00)	500 (0.00)	< 0.001

**Table 13:** Comparison of state-of-the-art methods with varying IR (Example 3).

Metric	IR	FCM CWD	FCM-${ℒ}_{1}$	*K*MEAN	Self-Updating	BKIFF	Dinh Pham-Toan (2025) [28]	Friedman *p*-value
*ARI*	20	0.12 (0.19)	0.12 (0.19)	1.00 (0.00)	1.00 (0.00)	1.00 (0.00)	0.00 (0.00)	< 0.001
	50	0.01 (0.00)	0.01 (0.00)	1.00 (0.00)	1.00 (0.00)	1.00 (0.00)	0.00 (0.00)	< 0.001
	80	< 0.01 (0.00)	< 0.01 (0.00)	0.02 (0.00)	1.00 (0.00)	1.00 (0.00)	0.00 (0.00)	< 0.001
	100	< 0.01 (0.00)	< 0.01 (0.00)	0.02 (0.00)	1.00 (0.00)	1.00 (0.00)	0.00 (0.00)	< 0.001
*NMI*	20	0.14 (0.12)	0.14 (0.12)	1.00 (0.00)	1.00 (0.00)	1.00 (0.00)	0.00 (0.00)	< 0.001
	50	0.04 (0.00)	0.04 (0.00)	1.00 (0.00)	1.00 (0.00)	1.00 (0.00)	0.00 (0.00)	< 0.001
	80	0.03 (0.00)	0.03 (0.00)	0.04 (0.00)	0.00 (0.00)	1.00 (0.00)	0.00 (0.00)	< 0.001
	100	0.02 (0.00)	0.02 (0.00)	0.03 (0.00)	0.00 (0.00)	1.00 (0.00)	0.00 (0.00)	< 0.001
*Dunn* Index	20	0.00 (0.00)	0.00 (0.00)	6.31 (0.00)	6112 (0.00)	6.31 (0.00)	0.00 (0.00)	< 0.001
	50	0.00 (0.00)	0.00 (0.00)	7.66 (0.00)	5996 (0.00)	7.66 (0.00)	0.00 (0.00)	< 0.001
	80	0.00 (0.00)	0.00 (0.00)	0.00 (0.00)	141 (0.00)	6.68 (0.00)	0.00 (0.00)	< 0.001
	100	0.00 (0.00)	0.00 (0.00)	0.00 (0.00)	267 (0.00)	5.80 (0.00)	0.00 (0.00)	< 0.001
*Silhouette*	20	0.41 (0.29)	0.41 (0.28)	0.99 (0.00)	1.00 (0.00)	0.99 (0.00)	0.00 (0.00)	< 0.001
	50	0.19 (0.01)	0.19 (0.01)	0.99 (0.00)	1.00 (0.00)	0.99 (0.00)	0.00 (0.00)	< 0.001
	80	0.23 (0.00)	0.23 (0.00)	0.34 (0.00)	1.00 (0.00)	0.99 (0.00)	0.00 (0.00)	< 0.001
	100	0.25 (0.00)	0.25 (0.00)	0.36 (0.00)	1.00 (0.00)	0.99 (0.00)	0.00 (0.00)	< 0.001
Comp. Time	20	0.01 (0.01)	0.01 (0.01)	0.01 (0.00)	2.25 (0.01)	0.02 (0.00)	6.62 (0.00)	< 0.001
	50	0.03 (0.01)	0.02 (0.01)	0.04 (0.02)	13 (3.90)	0.04 (0.02)	17 (0.00)	< 0.001
	80	0.03 (0.01)	0.02 (0.01)	0.06 (0.02)	34 (0.96)	0.07 (0.00)	26 (0.00)	< 0.001
	100	0.04 (0.02)	0.03 (0.02)	0.07 (0.03)	49 (6.90)	0.11 (0.02)	32 (0.00)	< 0.001
Iterations	20	8.00 (5.00)	8.00 (5.00)	3.50 (1.00)	4.00 (0.00)	5.00 (1.00)	500 (0.00)	< 0.001
	50	9.50 (7.00)	9.50 (7.00)	12 (5.00)	5.00 (0.00)	7.00 (1.00)	500 (0.00)	< 0.001
	80	7.00 (3.00)	7.00 (3.00)	14 (4.00)	5.00 (0.00)	9.00 (1.00)	500 (0.00)	< 0.001
	100	7.00 (3.00)	7.00 (3.00)	11 (3.00)	4.00 (0.00)	10 (1.00)	500 (0.00)	< 0.001

Specifically, FCM-CWD and FCM-${ℒ}_{1}$ exhibit an immediate decline in clustering quality as imbalance intensifies. Across the three examples, their *ARI*, *NMI*, and Dunn indices decline rapidly toward zero, while their Silhouette scores stagnate at low levels, indicating poor cluster separation. The *K*-MEAN algorithm remains competitive only under mild imbalance, with *IR* = 20 and *IR* = 50. However, its performance degrades substantially for *IR* values over 80, where both accuracy and separation metrics deteriorate. The Self-Updating method preserves *ARI* = 1.0 across several configurations; however, it produces tremendous and unstable Dunn values, reflecting pathological behaviour rather than meaningful cluster structure. The method proposed by Dinh Pham-Toan (2025) [28] exhibits the weakest performance among all evaluated approaches. In Examples 2 and 3, it yields *ARI* = *NMI* = 0.000 across all imbalance levels, indicating a complete failure to recover any meaningful clustering structure. In Example 1, where several methods already achieve perfect clustering, Dinh’s method does not offer benefits when dealing with imbalanced data. Across all examples and evaluation metrics, Friedman tests return *p*-values below 0.001, confirming that the observed performance differences among methods are statistically significant.

From a computational perspective, BKIFF also demonstrates the most favourable and stable cost profile, which is comparable to the baseline. While FCM-CWD and FCM-${ℒ}_{1}$ incur relatively modest runtimes, they require a large number of iterations. The *K*MEAN algorithm remains computationally inexpensive. Although the Self-Updating method converges in as few as 3÷5 iterations, its wall-clock time grows rapidly, exceeding 10s in Example 2 and approaching 50s in Example 3, highlighting that a low iteration count does not necessarily translate into computational efficiency. In contrast, BKIFF consistently converges within approximately 5÷11 iterations, with runtimes ranging from 0.01÷0.14 s even under extreme imbalance conditions. By comparison, the method proposed by Dinh Pham-Toan (2025) [28] repeatedly reaches the maximum iteration limit of 500. It exhibits the longest runtimes across all examples, often extending to tens of seconds in Examples 2 and 3, which demonstrates pronounced scalability limitations. Friedman tests for both runtime and iteration counts uniformly yield *p* < 0.001, confirming that BKIFF’s computational advantage over all competing methods is statistically significant.

**Fig 12:**
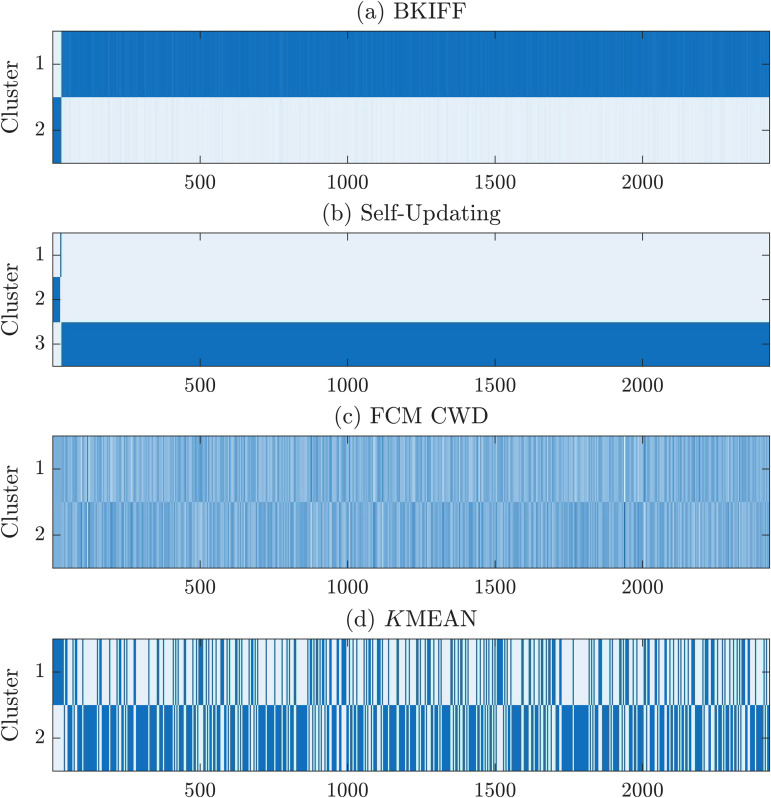
Membership matrix of methods at *IR* = 80.

By the qualitative membership results of the typical example 3, panels of Fig 12(a)–Fig 12(d) display the per/sample membership of each cluster algorithm (except the FCM-$L_{1}$) for *IR* = 80. Fig 12 shows that BKIFF maintains perfect crispness in this setting, pointing to a previously underexplored imbalanced-immunity threshold beyond which centroid-based and self-updating methods may struggle to maintain reliable partitions.

### 5.5 Application results

**Fig 13:**
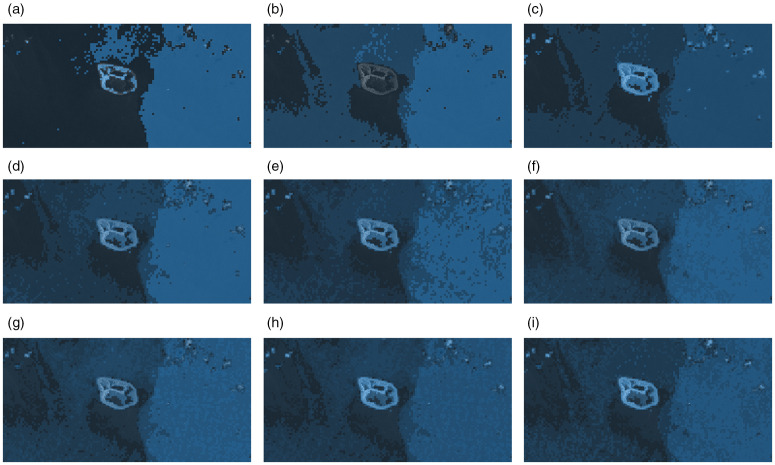
The proposed method BKIFF applied to the Landsat image.

Fig 13 shows the results of the BKIFF cluster analysis with an increasing number of clusters *c* from 2÷10 applied to segment the Landsat image of the Yam island and its surrounding sea. It shows that the level of detail of land cover separation increases with *c*-clusters.

As a result, above c=2÷10, the BKIFF segmentation progresses monotonically without producing salt-and-pepper artefacts. At *c* = 2, the algorithm separates the scene into deep water and the surrounding coastal areas. As the *c*-cluster increases toward 3, the spectral contrast between the coral-reef flats and the island becomes clearer, revealing more defined outlines. Between *c* = 4 and *c* = 6, the island becomes more distinct, showcasing areas of light sand, vegetation, and various marine regions. Additionally, a new layer is introduced to highlight differences in the seawater. Next, micro-layers c=7÷9 features, such as thin sandbars occupying less than 0.5% of the total pixels, remain clustered, demonstrating the algorithm’s capacity to preserve minority spectral signatures despite extreme class imbalances. Finally, at *c* = 10, no clusters are disrupted; each habitat remains a spatially contiguous blob, demonstrating the algorithm’s intrinsic spatial consistency.

In conclusion, an average clustering degree of *c* between 4 and 6 is most appropriate for balancing coverage, discrimination, and generalisability. In contrast, higher values of *c* are better suited for microscopic studies that require deeper investigation and fine-grained decomposition.

## 6 Discussion

The experimental findings provide several important insights when positioning BKIFF within the broader landscape of clustering methods for uncertain and imbalanced data. First, classic partition-based algorithms such as k-means or standard FCM, whose performance heavily depends on centroid initialisation and tends to deteriorate under skewed distributions, BKIFF demonstrates that an informed initialisation strategy can fundamentally alter the optimisation landscape. Another notable insight is the interaction between *IR*, fuzziness parameter *m*, and distance metrics. Traditional FCM-based methods tend to collapse at relatively low *m* under high *IR*, whereas BKIFF extends. This indicates that the proposed framework implicitly regularises the membership distribution, making it less sensitive to over-smoothing effects induced by large fuzziness values. In conclusion, from a methodological perspective, BKIFF’s consistent performance across all experimental scenarios suggests that the combination of adaptive initialisation and an appropriate probabilistic distance constitutes a more effective strategy than introducing additional model complexity.

### 6.1 Advantages of the current study

One of the significant advantages of the BKIFF algorithm is its capability to eliminate reliance on randomness, a limitation often found in previous clustering algorithms that employ adaptive initialisation. Traditional methods may require multiple trials to achieve an optimal initialisation. Randomness causes instability in partition clustering, leading to slower convergence rates and potentially incorrect solutions. Therefore, BKIFF enhances clustering accuracy from the first iterations. The biologically inspired BKO step provides high-quality initial prototypes, allowing the clustering process to converge much faster, enabling the process to be deterministic, thereby delivering superior temporal stability and reliability in high-demand, large-scale applications.

Moreover, conventional clustering methods often assign higher membership values to samples belonging to large clusters. In Comparison, minority clusters exhibit lower membership values. This imbalance in membership assignment can result in small clusters being misrepresented or absorbed by dominant clusters, leading to inaccurate results. BKIFF presents a two-phase clustering approach that addresses this issue by incorporating a more refined membership assignment mechanism, ensuring a fairer representation of all clusters.

Beyond its methodological refinements, the theory of BKIFF has been formally established through Zangwill’s convergence theory, i.g, it has proven to fulfil the conditions for convergence as in Theorem 4. Likewise, traditional clustering metrics based on ${ℒ}_{1}$- or ${ℒ}_{2}$-distance consistently struggle with imbalanced datasets. Consequently, blending an initialisation strategy, a theoretically sound fuzzy clustering model, and an advanced similarity measure makes BKIFF reliable for uncertain and imbalanced data.

Finally, applying the pdfs to the Yam Island image analysis based on colour distribution is interesting. We do not necessarily need to emphasize deeper technicals to recognize each object on the Island, as it already has extremely high colour correlation, allowing for a less resource-intensive algorithm. Therefore, this application is a novel and promising approach in image processing.

### 6.2 Limitations

The improvement BKIFF focuses on observing the invariant behaviour in the environment of increasing imbalance. The innovations in determining the legitimacy and trustworthiness of the number of clusters *c*, in discovering complexities of overlapping, high-dimensional data, and in extending to multi-view clustering [54,55] must be further developed. Furthermore, the indices related to internal quality are also studied more deeply because of the ambiguity about its specific distances.

Moreover, real-world uncertain data are challenging to collect and analyse due to their complexity and the conditions under which they are collected. Therefore, the uncertainty assessment for natural structure analysis and imbalance detection based on random image cropping (i.e., Example 2) is an exciting application. Nevertheless, caution is needed in cluster analysis of density functions. Truthfully, the density functions of colour are generally invariant when the image is rotated, scaled, and contains a small amount of noise [56]. It is noticeable that colour density functions can exemplify the similarity between features in images; however, not all of them. It raises considerable challenges because colour is not permanently a more attractive feature than the object within the image in many difficulties, such as detection or classification. In summary, the density functions have advantages over the intensity matrix, but this feature can describe image similarities only for specific problems and needs further improvement.

## 7 Conclusion

This study proposed the BKIFF algorithm, a two-phase framework designed to address the challenge of clustering pdfs under severe class imbalance. Building on this foundation, the method seamlessly integrated an enhanced initialisation process with imbalance-aware fuzzy clustering, thereby addressed the shortcomings of existing approaches while ensuring provable convergence. Moreover, extensive experiments demonstrate that BKIFF consistently achieved high clustering accuracy and computational efficiency across a wide range of imbalance ratios. In addition, its effectiveness was validated through Landsat image segmentation, where it successfully preserved minority spectral structures even under highly skewed conditions. Taken together, these results showed that BKIFF offers a robust and practical solution for clustering uncertain data. Future research may focus on the automatic determination of the number of clusters and explore extensions toward deep learning or ensemble-based clustering strategies.

After overcoming the inherent shortcomings, several potential research directions can be considered. These include the integration of deep clustering or ensemble clustering to increase the exploration of the pdf object and further extensions toward contrastive learning for images and videos [57]. In addition, more automatics will be applied to the optimal clustering phase [58] and accommodate a wider range of data types [59]. Furthermore, the proposed density function framework shows strong potential to provide more interpretable insights and theoretical support across various application domains, such as daily solar radiation analysis [60] and species distribution modelling. Finally, handling imbalanced data remains a promising direction, where advanced resampling techniques can be further explored and integrated [61,62].

### A Proofs of convergence

By this procedure, an iterative sequence $\left\{U^{\left(t\right)}\;,\Theta^{\left(t\right)}\right\}$ is generated, and the theoretical task is to resolve whether or not the sequence converges. Consider *F* and *G* as two functions $F\;:\;{\mathbb{R}}^{cd}\to U_{f}\;,F(\Theta )=F(\theta_{1}\;,\theta_{2}\;,\ldots \;,\theta_{c})=\mu \;,\text{and }G\;:\;U_{f}\to {\mathbb{R}}^{cd}\;,G(\mu )=\Theta =(\theta_{1}\;,\theta_{2}\;,\ldots \;,\theta_{c})$. Using F and G, we must modify *T*_*IFF*_ so that it generates Picard sequences in both μ and Θ simultaneously. From here, we define the operator


$$\begin{array}{c} T_{IFF}\;:\;{\mathbb{R}}^{cd}\times U_{f}\to {\mathbb{R}}^{cd}\times U_{f}\;,T_{IFF}=T_{2}\circ T_{1}, \end{array}$$ (11)

where, the mapping $T_{1}\;:\;{\mathbb{R}}^{cd}\times U_{f}\to {\mathbb{R}}^{cd}\;,T_{1}(\mu \;,\Theta )=G(\mu )$, and the mapping $T_{2}\;:\;{\mathbb{R}}^{cd}\to {\mathbb{R}}^{cd}\times U_{f}\;,T_{2}(\Theta )=(F(\Theta ),\Theta )$.

In summary, the proof is considered whether or not $\{T_{IFF}^{\left(t\right)}\left(\mu^{\left(0\right)}\;,\Theta^{\left(0\right)}\right)\}=\left((F\circ G{)}^{\left(t\right)}(\mu^{\left(0\right)}),G^{\left(t\right)}(\mu^{\left(0\right)})\right)$ convergent. The following two lemmas confirm the establishment of two formulas for the partition matrix and the prototypes given by the update for IFF and determine that the descent constraint holds for *J*_*IFF*_ in Zangwill’s theorem [63]. The fuzzifier parameter in the proposed algorithm is typically set to *m* > 1.

**Lemma 1 (Improved Fuzzy membership degree).** *Let* $\varphi \;:\;U_{f}\to \mathbb{R}$ *and the OF* $\varphi (\mu )=J_{IFF}(\mu \;,\Theta )$*, where* Θ *is fixed. Then* $\mu^{*}\in U_{f}$ *is a strict local minimum solution of* φ *if and only if* $\mu^{*}=F(\Theta )$*, and*


$$\begin{array}{c} \mu_{ij}\;=\;\frac{1}{\sum_{s=1}^{c}{\left(\frac{{ℋ}^{2}(\theta_{i},f_{j})/\omega_{i}}{{ℋ}^{2}(\theta_{s},f_{j})/\omega_{s}}\right)}^{\frac{1}{m-1}}},i\in {\mathbb{N}}_{\leq c},\;j\in {\mathbb{N}}_{\leq n}. \end{array}$$ (12)

**Proof 1** *Minimisation of* φ *over* ***U***_*f*_ *is an optimisation Karush-Kuhn-Tucker problem with (cn+n)-linear constraints. The original optimisation problem is rewritten as*


$$\begin{array}{c} \text{Minimise}\;:\;\varphi (\mu ). \end{array}$$

$$\begin{array}{c} \text{S.t. }\;:\;y_{ij}(\mu )=\mu_{ij}-1\leq 0, \end{array}$$ (13)

$$\begin{array}{c} z_{ij}(\mu )=-\mu_{ij}\leq 0, \end{array}$$ (14)

$$\begin{array}{c} \zeta_{j}(\mu )=\sum_{i=1}^{c}\mu_{ij}-1=0,\;i\in {\mathbb{N}}_{\leq c},\;j\in {\mathbb{N}}_{\leq n}. \end{array}$$

*Suppose that* $\mu^{*}$ *is a minimiser of the above objective function* φ(μ)*. Then, it must satisfy the following KKT conditions* [44,63]

- *The partition* $\mu^{*}$ *is feasible, i.e.,* $0\leq \mu_{ij}^{*}\leq 1$ *and* $\sum_{i=1}^{c}\mu_{ij}^{*}=1$;
- *There exist multipliers* $\lambda_{j}$ *for the equality constraints and* $\tau_{ij},\gamma_{ij}\geq 0$ *for the inequality constraints such that complementary slackness holds;*
- *Stationarity:*
$$\begin{array}{c} \frac{\partial \varphi }{\partial \mu_{ij}}(\mu^{*})+\tau_{ij}-\gamma_{ij}+\lambda_{j}=0. \end{array}$$ (15)
  *Since* $\mu_{ij}^{*}>0$*, we have* $\tau_{ij}=0$*. Similarly,* $\mu_{ij}^{*}<1$ *so that* $\gamma_{ij}=0$*. Therefore,* $\frac{1}{\omega_{i}}m(\mu_{ij}^{*}{)}^{m-1}{ℋ}^{2}(\theta_{i},f_{j})-\lambda_{j}=0.$ *So that,* $\mu_{ij}^{*}={\left(\frac{\lambda_{j}\omega_{i}}{m\;{ℋ}^{2}(\theta_{i},f_{j})}\right)}^{\frac{1}{m-1}}.$ *Recall the* constant $\sum_{i=1}^{c}\mu_{ij}^{*}=1$*, we have (12).*
- *To the sufficiency, we examine the* $H_{\varphi }(\mu )$*, the* (*cn* × *cn*) *Hessian matrix of* φ *evaluated at* $\mu_{ij}\in U_{f}$*. It is easy to reduce that*
  $$\begin{array}{c} \frac{\partial^{2}}{\partial \mu_{ts}\partial \mu_{pq}}\varphi =\left\{ \begin{array}{c} \xi_{ts},\text{ for any }t=p,s=q,\\ 0,\text{otherwise} \end{array}\right. \end{array}$$
  *where* $\xi_{ij}=\frac{1}{\omega_{i}}m(m-1)(\mu_{ij}{)}^{m-2}{ℋ}^{2}(\theta_{i},f_{j})$*. Since we assume m > 1 and distance is non-zero in this section, accordingly* $H_{\varphi }(\mu )$ *is a positive definite matrix with all the diagonal elements* $\xi_{ij}$ *positive. Therefore,* $\mu_{ij}$*, calculated by (12), is the solution to the relaxed optimisation problem under consideration.*

Next, we fix $\mu \in U_{f}$ and consider minimisation of *J*_*IFF*_ with respect to Θ.

**Lemma 2.** *Let* $\psi \;:{\mathbb{R}}^{cp}\to \mathbb{R},\psi (\Theta )=J_{IFH}(\mu ,\Theta )$ *is Hellinger-distance around prototype calculated when* μ *is constant (*$\mu \in {\text{U}}_{f}$*). Then,* $\Theta^{*}$ *is a strict local minimum solution of* ψ *if and only if* $\Theta^{*}=G(\mu )$ *formed by following*


$$\begin{array}{c} \theta_{i}(x)={\left(\frac{\sum_{j=1}^{n}(\mu_{ij}{)}^{m}\sqrt{f_{j}(x)}}{\sum_{j=1}^{n}(\mu_{ij}{)}^{m}}\right)}^{2}\;,1\leq i\leq c. \end{array}$$ (16)

**Proof 2** *For fixed membership matrix* $\mu \in {𝐔}_{f}$*, the objective function* $\psi (\Theta )=J_{IFH}(\mu ,\Theta )=\sum_{i=1}^{c}\sum_{j=1}^{n}(\mu_{ij}{)}^{m}{ℋ}^{2}(f_{j},\theta_{i})$ *is separable in the prototype functions* $\theta_{i}$*. Using the squared Hellinger distance, we obtain*

$\begin{array}{c} \psi (\Theta )=\sum_{i=1}^{c}\int \left[\sum_{j=1}^{n}(\mu_{ij}{)}^{m}{\left(\sqrt{f_{j}(x)}-\sqrt{\theta_{i}(x)}\right)}^{2}\right]\;dx. \end{array}$

*For each i, the integrand is a quadratic polynomial in* $\sqrt{\theta_{i}(x)}$*. Differentiating twice with respect to* $\theta_{i}(x)$ *gives*

$\frac{\partial^{2}\psi }{\partial \theta_{t}(x)\;\partial \theta_{s}(x)}=\left\{ \begin{array}{cc} \sum_{j=1}^{n}(\mu_{tj}{)}^{m}, & t=s,\\ 0, & t\neq s. \end{array}\right.$

*Since* $\mu \in {𝐔}_{f}$ *implies* $\sum_{j}\mu_{ij}>0$, *we also have* $\sum_{j}(\mu_{ij}{)}^{m}>0$ *for all i. Therefore the Hessian* ${𝐇}_{\psi }(\Theta )$ *is diagonal with strictly positive diagonal entries, hence positive definite. Thus* ψ *is strictly convex on* ${\mathbb{R}}^{cp}$.

*Strict convexity ensures that* $\Theta^{*}$ *minimizes* ψ *if and only if it satisfies* $\frac{\partial \psi }{\partial \theta_{i}(x)}=\sum_{j=1}^{n}(\mu_{ij}{)}^{m}\frac{\partial }{\partial \theta_{i}(x)}{\left(\sqrt{f_{j}(x)}-\sqrt{\theta_{i}(x)}\right)}^{2}=0.$

*Computing the derivative gives*

$\begin{array}{c} \frac{\partial }{\partial \theta_{i}(x)}{\left(\sqrt{f_{j}(x)}-\sqrt{\theta_{i}(x)}\right)}^{2}=-\;\frac{\sqrt{f_{j}(x)}-\sqrt{\theta_{i}(x)}}{\sqrt{\theta_{i}(x)}}. \end{array}$

*Hence, the optimality condition becomes* $\sum_{j=1}^{n}(\mu_{ij}{)}^{m}\left(\sqrt{f_{j}(x)}-\sqrt{\theta_{i}^{*}(x)}\right)=0$*. Next, solving for* $\sqrt{\theta_{i}^{*}(x)}$ *yields* $\begin{array}{c} \sqrt{\theta_{i}^{*}(x)}=\frac{\sum_{j=1}^{n}(\mu_{ij}{)}^{m}\sqrt{f_{j}(x)}}{\sum_{j=1}^{n}(\mu_{ij}{)}^{m}}, \end{array}$ *and therefore, we have*

$\begin{array}{c} \theta_{i}^{*}(x)={\left(\frac{\sum_{j=1}^{n}(\mu_{ij}{)}^{m}\sqrt{f_{j}(x)}}{\sum_{j=1}^{n}(\mu_{ij}{)}^{m}}\right)}^{2}=G_{i}(\mu )(x). \end{array}$

*Since the objective is strictly convex, this solution is the unique strict global minimum.*

The above two lemmas are only necessary conditions for $\left(\mu^{*}\;,\Theta^{*}\right)$ to be a global minimum of the function *J*_*IFF*_. From here, the next question is whether or not the iterate sequence $\left\{T_{IFF}^{\left(t\right)}(\mu^{\left(0\right)})\right\}$ converges to $\mu^{*}$.

The final condition required for the Zangwill theorem is the compactness of a subset of $U_{f}\times {\mathbb{R}}^{cd}$ which contains all of the possible iterative sequences generated by *T*_*IFF*_. The three theorems 1, 2, and 3 are referred to in [31,44,64]

These three theorems are part of the proof of IFF by Zangwill’s theorem that directly supports the results of Theorem 4.

**Theorem 1 (Compactness constraint).** *Let* $[conv(ℱ){]}^{c}$ *be the c-fold Cartesian product of the convex hull of* ℱ*, and* $\left(\mu^{\left(0\right)}\;,\Theta^{\left(0\right)}\right)$ *be the starting point of iteration with* *J*_*IFF*_ *with* $\mu^{\left(0\right)}\in U_{f}$ *and* $\theta^{\left(0\right)}=G(\mu^{\left(0\right)})$*. Then*


$$\begin{array}{c} T_{IFF}^{\left(0\right)}\left(\mu^{\left(0\right)}\;,\Theta^{\left(0\right)}\right)\in U_{f}\times [conv(ℱ){]}^{c}, \end{array}$$

*and* $U_{f}\times [conv(ℱ){]}^{c}$ *is compact in* $U_{f}\times {\mathbb{R}}^{cn}$.

**Proof 3** *Let* $\mu^{\left(0\right)}\in U_{f}$ *be chosen, then* $\theta^{\left(0\right)}=G(\mu^{\left(0\right)})$ *calculated by (12). So that*


$$\begin{array}{c} \theta_{i}^{\left(0\right)}=\frac{\sum_{j=1}^{n}{\left(\mu_{ij}^{\left(0\right)}\right)}^{m}f_{j}}{\sum_{j=1}^{n}{\left(\mu_{ij}^{\left(0\right)}\right)}^{m}}. \end{array}$$

*Let* $\rho_{ij}=\frac{{\left(\mu_{ij}^{\left(0\right)}\right)}^{m}}{\sum_{l=1}^{n}{\left(\mu_{il}^{\left(0\right)}\right)}^{m}}\;,1\leq j\leq n$*. In view of constraints (C1) and (C3), it must be that* $0<\rho_{ij}<1$, ∀i,j*. So* ∀i*, we rewrite* $\theta_{i}^{\left(0\right)}=\sum_{j=1}^{n}\rho_{ij}f_{j}$*, with*


$$\begin{array}{c} \sum_{j=1}^{n}\rho_{ij}=\sum_{j=1}^{n}\left(\frac{{\left(\mu_{ij}^{\left(0\right)}\right)}^{m}}{\sum_{l=1}^{n}{\left(\mu_{il}^{\left(0\right)}\right)}^{m}}\right)=\frac{\sum_{l=1}^{n}{\left(\mu_{il}^{\left(0\right)}\right)}^{m}}{\sum_{l=1}^{n}{\left(\mu_{il}^{\left(0\right)}\right)}^{m}}=1. \end{array}$$

*Thus,* $\forall i\;,\theta_{i}^{\left(0\right)}\in conv(ℱ)$*, and hence* $\theta^{\left(0\right)}\in [conv(ℱ){]}^{c}$*. Continuing recursively, we know that* $\mu^{\left(1\right)}=F(\mu^{\left(0\right)})\in U_{f}$ *by (12), and then* $\theta^{\left(1\right)}=G(\mu^{\left(1\right)})\in [conv(ℱ){]}^{c}$ *by the same argument as above. Thus, every iterative sequence of* *J*_*IFF*_ *belongs to* $U_{f}\times [conv[(ℱ){]}^{c}$ *for any* *t* ≥ 1*. Although we may choose* $\theta^{\left(0\right)}\in {\mathbb{R}}^{cd}⧵conv(ℱ){]}^{c}$ *because its initialisation,* $\mu^{\left(0\right)}=F(\theta^{\left(0\right)})\in U_{f}$*, so that* $\theta^{\left(t\right)}=G(\mu^{\left(t\right)})\in [conv(ℱ){]}^{c}\;,\forall t\geq 1$*. Furthermore, it is clear that* $U_{f}\times [conv(ℱ){]}^{c}$ *is a compact set in* ℱ *finite* [44].

**Theorem 2.** *Consider* $S_{IFF}=\{(\mu^{*},\Theta^{*})\;:\;J_{IFF}(\mu^{*},\Theta^{*})<J_{IFF}(\mu ,\Theta )\;,\forall (\mu ,\Theta )\in B^{0}((\mu^{*},\Theta^{*}),r)\}$ *be the solution set. Then,* *J*_*IFF*_ *is a descent function for* $\left\{T_{IFF}\;,S_{IFF}\right\}$*.*

**Proof 4** *First, since* $\left\{y\to \|y{\|}^{2}\right\}$*,* {y→1−y}*, and* $\left\{y\to y^{m}\right\}$ *are continuous, and* *J*_*IFF*_ *is the sum of products of such functions so* *J*_*IFF*_ *is continuous on* $U\times {\mathbb{R}}^{cd}$*. Next, suppose* $(\mu \;,\Theta )\notin S_{IFF}$*. Then it follows from (11) that*


$$\begin{array}{c} \begin{array}{ccc} J_{IFF}(T_{IFF}(\mu ,\Theta )) & =J_{IFF}\left(T_{2}\circ T_{1}(\mu \;,\Theta )\right)\\ & =J_{IFF}(F\circ G(\mu ),G(\mu )) & \text{by Definition }11\\ & <J_{IFF}(\mu \;,G(\mu )) & \text{by Lemma }1\\ & <J_{IFF}(\mu \;,\Theta ) & \text{by Lemma }2 \end{array} \end{array}$$

*Finally, Lemma 1 and Lemma 2 implies that* $(\mu ,\Theta )\in S_{IFF}$.

**Theorem 3 (Continuity constraint).** *T*_*IFF*_ *is continuous on* $U_{f}\times {\mathbb{R}}^{cn}$.

**Proof 5** *Since* $T_{IFF}=T_{2}\circ T_{1}$ *and the composition of continuous functions is again continuous, it suffices to show that T*_*1*_ *and T*_*2*_ *are each continuous. Since* $T_{1}(\mu ,\Theta )=G(\mu )$*, T*_*1*_ *is continuous if G is. To see that G is continuous in the cn variables* μ *note that G is a vector field, with the resolution by (cd) scalar field as* $G\;:\;{\mathbb{R}}^{cd}\to {\mathbb{R}}^{cp}$*. Now* $\left\{\mu \to \mu^{m}\right\}$ *is continuous,* $\left\{\mu^{m}\to \mu^{m}x_{ik}\right\}$ *is continuous. The sum of continuous functions is again continuous. Thus,* *G*_*ik*_ *is the quotient of two continuous functions. In view of constraint (C3), the denominator* $\sum_{j}\mu_{ik}^{m}$ *never vanishes, then* *G*_*ik*_ *are also continuous for all (i, k). Therefore, G and T*_*1*_ *are continuous on their entire domains.*

*Similarly, since* $T_{2}(\Theta )=(F(\Theta )\;,\Theta )$*, it suffices to show that F is a continuous function in the variable* $\left\{\theta_{i}\right\}$*. F is a vector field with the resolution by (cd) scalar fields* $F\;:\;{\mathbb{R}}^{cp}\to {\mathbb{R}}^{cd}$*. Since* $\left\{\theta_{i}\to {ℋ}^{2}(\theta_{i}\;,x_{j})\right\}$ *is continuous for all i, and* $\left\{{ℋ}^{2}(\theta_{i}\;,x_{j})\to ℋ(\theta_{i}\;,x_{j}{)}^{-2/(m-1)}\right\}$ *is continuous. The sum of continuous functions is again continuous; thus,* *F*_*ij*_ *is the quotient of two continuous functions. Given our general hypothesis that* ${ℋ}^{2}>0$*. Therefore, F and T*_*2*_ *are continuous on their entire domains. Finally.* $J_{IFF}=T_{2}\circ T_{1}$ *is continuous on* $U_{f}\times {\mathbb{R}}^{cd}$.

We now assemble the assumptions and results of the above lemmas into a formal theorem regarding the convergence of IFFs.

**Theorem 4 (Convergence theorem for** *J*_*IFF*_**).** *Consider the set* $ℱ=\{f_{1}\;,f_{2}\;,\ldots \;,f_{n}\}\subset {\mathbb{R}}^{d}$*, given the OF* $J_{IFF}(U\;,\Theta )$ *of the form (2), where* ***U*** *satisfies (C1)-(C3) and* $\Theta =\{\theta_{1}\;,\theta_{2}\;,\ldots \;,\theta_{c}\}$*. If* $T_{IFF}\;:\;{\mathbb{R}}^{cd}\times U_{f}\to {\mathbb{R}}^{cd}\times U_{f}$ *is an algorithm (Picard) iterative operator of* *J*_*IFF*_*, and for every t such that* $ℋ(\theta_{i}^{\left(t\right)}\;,f_{j})>0$ *then for any* $(\mu^{\left(0\right)}\;,\Theta^{\left(0\right)})\in U_{f}\times [conv(ℱ){]}^{c}$*, or*

1. $\left\{(J_{IFF}{)}^{\left(t\right)}(\mu^{\left(0\right)},\Theta^{\left(0\right)})\right\}$ *terminates at a local minimum* $\left(\mu_{ij}^{*},\Theta^{*}\right)$ *of* *J*_*IFF*_*; or*
2. $\left\{(J_{IFF}{)}^{\left(t\right)}(\mu^{\left(0\right)},\Theta^{\left(0\right)})\right\}$ *contains a subsequence such that* $\left\{(J_{IFF}{)}^{\left(t_{k}\right)}(\mu^{\left(0\right)},\Theta^{\left(0\right)})\}\to (\mu^{*},\Theta^{*}\right)$ *a local minimum of* *J*_*IFF*_ *as* $t_{k}\to \infty$.

**Proof 6** *Because of the OF* *J*_*IFF*_ *is continuous on* $U_{f}\times {\mathbb{R}}^{cd}$*, Theorem 2 shows that* *J*_*IFF*_ *is a Zangwill descent functional for the solution set* $\left\{T_{IFF}\;,S_{IFF}\right\}$ *where* *S*_*IFF*_ *is the set of strict local minima of* *J*_*IFF*_*. Theorem (3) asserts that iterative operator* *T*_*IFF*_ *is continuous on* $U_{f}\times [conv(ℱ){]}^{c}$ *and by Theorem (1), the iterate sequences operator* *J*_*IFF*_ *are always in a compact subset of the domain of* *J*_*IFF*_*. The result follows immediately from the Zangwill theorem.*

## B Hypothesis test results

### B.1 Sensitivity analysis of BKIFF

#### B.1.1 BKO-based initialisation.

**Fig 14:**
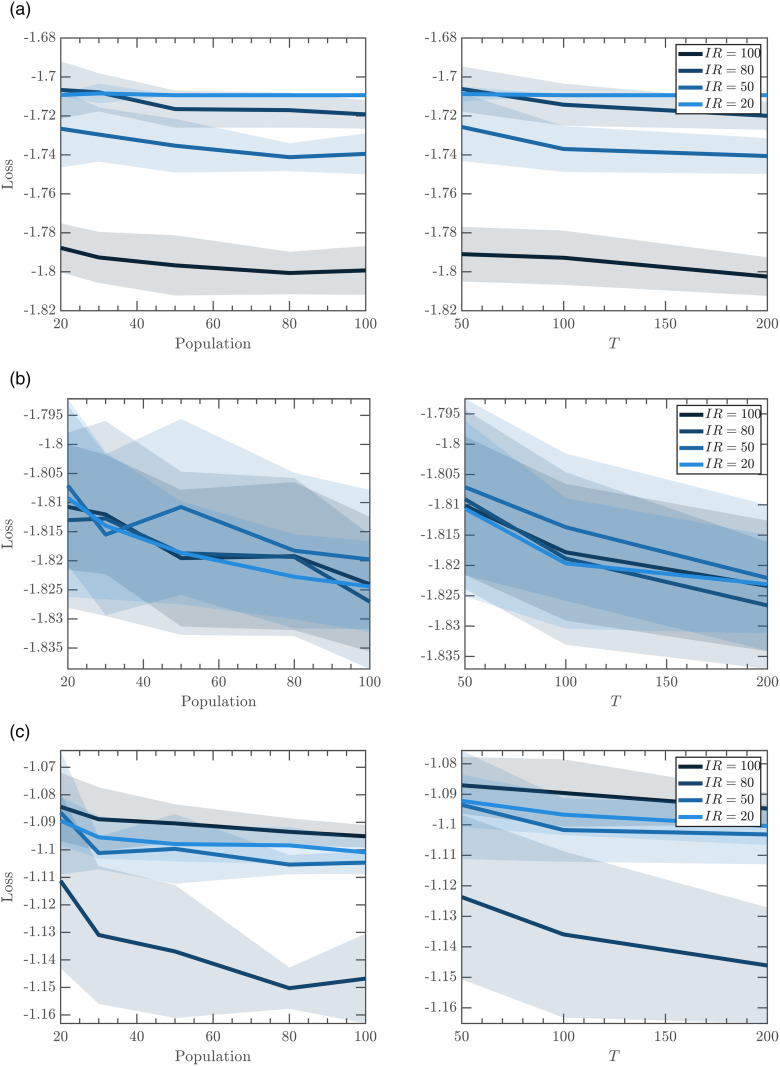
Sensitivity of population and $T_{\max}$ on BKO of each IR.

In all three Examples, the sensitivity to population size (*pop*) and iteration number ($T_{\max}$) makes almost no significant difference to the OF value. The Fig 14 corresponding to different *IR* levels exhibits a relatively stable trend, with only minor fluctuations within the error range, suggesting that the performance of BKO is robust to changes in the basic configuration parameters.

#### B.1.2 IFF clustering algorithrm.

Table 14 presents the results of the Morris and ANOVA sensitivity analyses for *ARI* across the three Examples. Each configurations repeated 10 times evaluations. Overall, the agreement between the Morris and ANOVA analyses highlights the dominant role of parameter *c*, the secondary but still meaningful influence of *IR* and *m*, and the weak and stable impact of the distance factor.

**Table 14:** Sensitivity metrics and ANOVA indices for *ARI* of three examples.

Example	Factor	Morris			ANOVA	
		μ	$\mu^{*}$	σ	$\eta^{2}$	*p*-value
Example 1	*c*	–0.12	0.41	1.02	0.06	< 0.001
	*IR*	–0.01	0.40	0.98	0.04	< 0.001
	*m*	0.05	0.28	0.69	0.05	< 0.001
	distance	< 0.01	0.14	0.44	0.01	< 0.001
Example 2	*c*	0.68	1.41	2.40	0.68	< 0.001
	*IR*	0.07	0.15	0.35	< 0.01	0.02
	*m*	–0.18	0.24	0.49	< 0.01	< 0.001
	distance	–0.26	0.44	1.22	0.05	< 0.001
Example 3	*c*	0.10	0.96	1.95	0.25	< 0.001
	*IR*	0.23	0.68	1.41	0.08	< 0.001
	*m*	–0.18	0.56	1.14	< 0.01	< 0.001
	distance	–0.14	0.36	1.01	0.02	< 0.001

Firstly, the Morris indices indicate that parameter *c* consistently exerts the strongest overall influence, showing the highest absolute effects $\mu^{*}$ and substantial variability σ, particularly in Example 2 where $\mu^{*}=1.41$ and σ=2.4. By contrast, the factors *IR* and *m* exhibit moderate effect sizes, while the distance parameter shows the smallest and most stable impacts, reflected in comparatively low $\mu^{*}$ values across all three Examples. Moreover, the ANOVA results reinforce these observations. In Example 2, *c* dominates the variance contribution with $\eta^{2}=0.68$, indicating a markedly higher explanatory power than the remaining factors. Example 3 shows a similar pattern, with *c* accounting for nearly one quarter of the variance, $\eta^{2}=0.25$, followed by *IR* with a moderate contribution, $\eta^{2}=0.08$. In Example 1, the effects of *c*, *IR*, and *m* are more balanced, although all remain statistically significant at *p* < 0.001. Across all Examples, the distance factor consistently yields the smallest $\eta^{2}$ values, confirming its limited influence on *ARI* compared with the other parameters.

Simulate the entire set of parameters, including *IR*, the sets *m* and *c*, and the distance measures. Each configuration is repeated 10 times. Tables 15–17 display the Sum of Squares (SS), Mean Square (MS), *F*-statistic, *p*-value, and influence coefficient $\eta^{2}$ for each factor in three independent examples, respectively. Because of the missing true label, Table 18 excludes *ARI*. These values are calculated using one-way ANOVA. The larger these values, the greater the factors’ influence on clustering quality at the statistical significance level *p*.

**Table 15:** Post-hoc of ANOVA for each factors (Example 1).

Criteria	Parameters	SS	MS	*F*-statistics	*p*-value	$\eta^{2}$
ARI	*IR*	4.77	3.00	64	< 0.001	0.04
	*m*	5.96	6.00	40	< 0.001	0.05
	*c*	7.35	3.00	99	< 0.001	0.06
	distance	1.06	3.00	14	< 0.001	0.01
Silhouette	*IR*	2.08	3.00	19	< 0.001	0.00
	*m*	6.94	6.00	32	< 0.001	0.02
	*c*	258	3.00	2394	< 0.001	0.58
	distance	20	3.00	186	< 0.001	0.04
Iterations	*IR*	6.52 × 10^5^	3.00	7.25	< 0.001	0.00
	*m*	1.02 × 10^7^	6.00	57	< 0.001	0.05
	*c*	4.20 × 10^7^	3.00	467	< 0.001	0.22
	distance	1.38 × 10^6^	3.00	15	< 0.001	0.01
Comp. Time	*IR*	939.00	3.00	389	< 0.001	0.14
	*m*	199	6.00	41	< 0.001	0.03
	*c*	255	3.00	106	< 0.001	0.04
	distance	1750	3.00	725	< 0.001	0.26

**Table 16:** Post-hoc of ANOVA for each factors (Example 2).

Criteria	Parameters	SS	MS	*F*-statistics	*p*-value	$\eta^{2}$
ARI	*IR*	0.55	3.00	6.64	< 0.001	0.00
	*m*	0.42	6.00	2.55	0.02	0.00
	*c*	321	3.00	3878	< 0.001	0.68
	distance	24	3.00	293	< 0.001	0.05
Silhouette	*IR*	0.01	3.00	0.47	0.70	0.00
	*m*	3.26	6.00	14	< 0.001	0.01
	*c*	329	3.00	2798	< 0.001	0.62
	distance	26	3.00	222	< 0.001	0.05
Iterations	*IR*	2656	3.00	436	< 0.001	0.17
	*m*	314	6.00	26	< 0.001	0.02
	*c*	826	3.00	136	< 0.001	0.05
	distance	2380	3.00	391	< 0.001	0.16
Comp. Time	*IR*	6.84 × 10^6^	3.00	73	< 0.001	0.03
	*m*	2.60 × 10^6^	6.00	14	< 0.001	0.01
	*c*	7.09 × 10^7^	3.00	752	< 0.001	0.31
	distance	9.02 × 10^6^	3.00	96	< 0.001	0.04

**Table 17:** Post-hoc of ANOVA for each factors (Example 3).

Criteria	Parameters	SS	MS	*F*-statistics	*p*-value	$\eta^{2}$
ARI	*IR*	33.17	3.00	172	< 0.001	0.07
	*m*	2.46	6.00	6.37	< 0.001	0.01
	*c*	112	3.00	578	< 0.001	0.25
	distance	11	3.00	56	< 0.001	0.02
Silhouette	*IR*	12.93	3.00	56	< 0.001	0.02
	*m*	30	6.00	65	< 0.001	0.06
	*c*	110	3.00	477	< 0.001	0.21
	distance	31	3.00	135	< 0.001	0.06
Iterations	*IR*	1.21 × 10^6^	3.00	21	< 0.001	0.01
	*m*	1.81 × 10^6^	6.00	16	< 0.001	0.02
	*c*	5.80 × 10^6^	3.00	102	< 0.001	0.06
	distance	1.57 × 10^6^	3.00	28	< 0.001	0.02
Comp. Time	*IR*	11670	3.00	846	< 0.001	0.19
	*m*	1155	6.00	42	< 0.001	0.02
	*c*	4764	3.00	345	< 0.001	0.08
	distance	24571	3.00	1781	< 0.001	0.39

**Table 18:** Post-hoc of ANOVA for each factors (Application).

Criteria	Parameters	SS	MS	*F*-statistics	*p*-value	$\eta^{2}$
Silhouette	*m*	5.90	6.00	135	< 0.001	0.17
	*c*	15	7.00	298	< 0.001	0.44
	distance	540	3.00	2.47	0.01	0.00
Iterations	*m*	4.15 × 10^6^	6.00	50	< 0.001	0.09
	*c*	8.01 × 10^6^	8.00	72	< 0.001	0.17
	distance	3.02 × 10^5^	3.00	7.22	< 0.001	0.01
Comp. Time	*m*	94291	6.00	199	< 0.001	0.09
	*c*	4.31 × 10^5^	8.00	680	< 0.001	0.42
	distance	3.13 × 10^5^	3.00	1320	< 0.001	0.30

### B.2 Pair configuations

**Fig 15:**
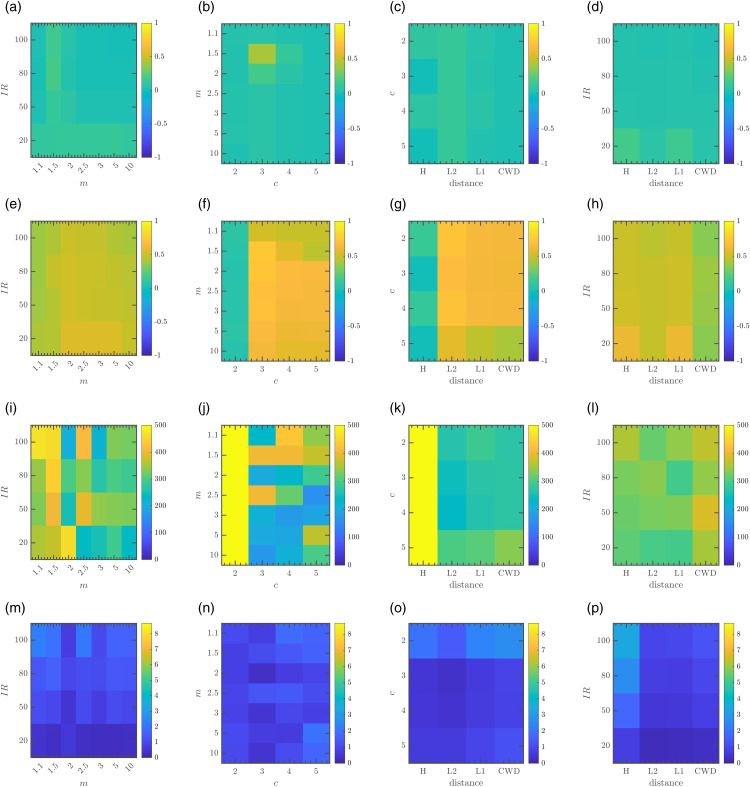
Correlation of pair factors of BKIFF (Example 1).

**Fig 16:**
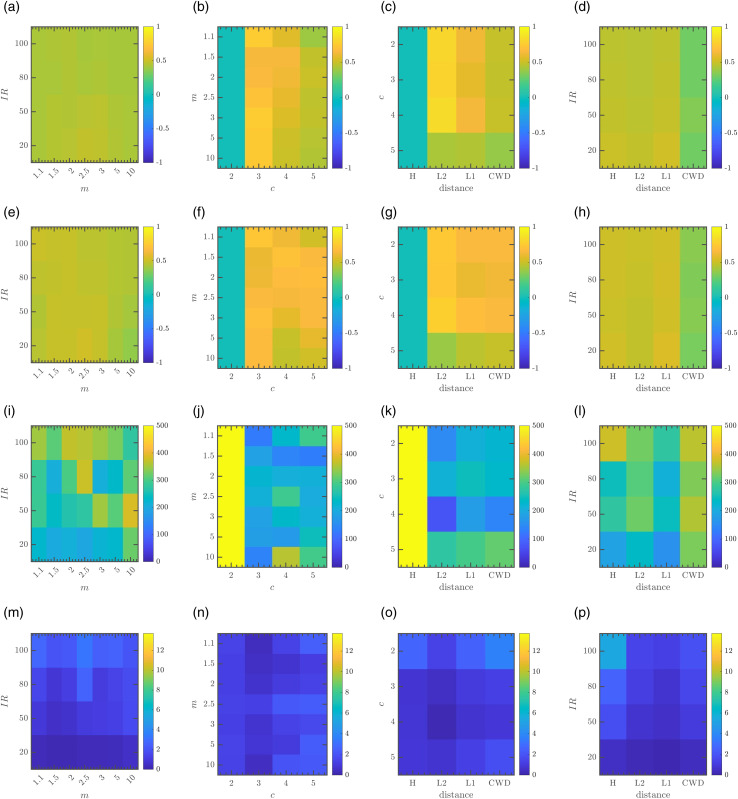
Correlation of pair factors of BKIFF (Example 2).

**Fig 17:**
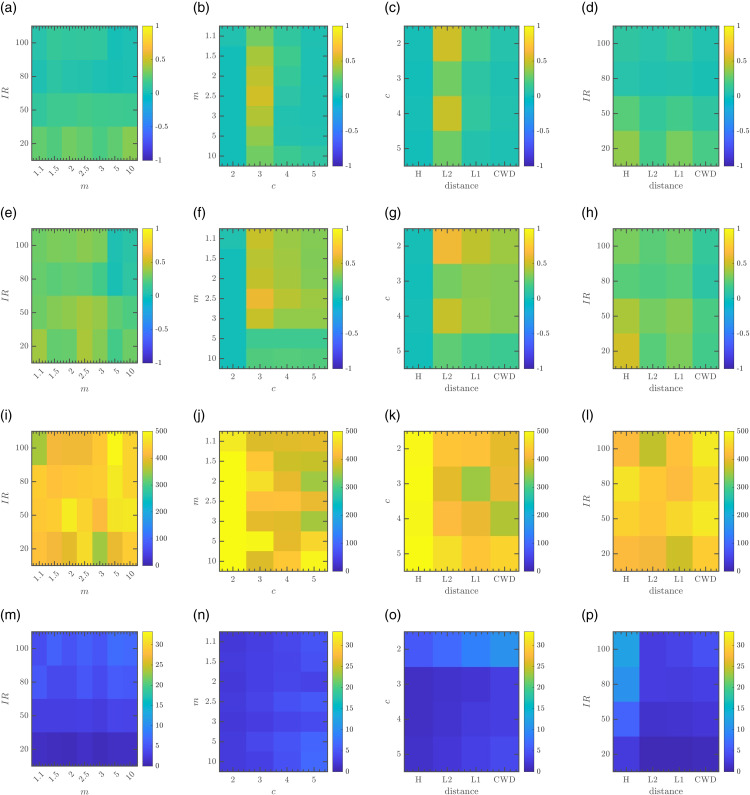
Correlation of pair factors of BKIFF (Example 3).

**Fig 18:**
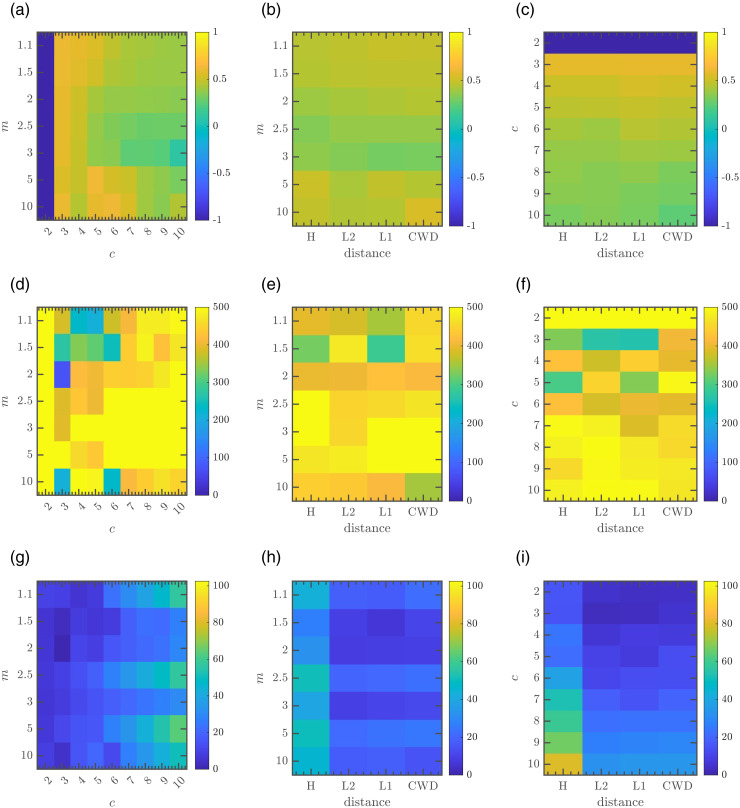
Correlation of pair factors of BKIFF (Application).

The Figs 15–17 show the correlation between each pair of BKIFF parameters, including *IR*, *c*, *m*, and distance for three Examples, respectively. Each coloured box shows the degree of correlation between the two factors across the four criteria, including *ARI*, *Silhouette*, number of iterations, and computation time. Because of the missing true label, Fig 18 excludes *ARI*. Light colours indicate strong correlation, while dark blue indicates weak or almost nonexistent effects. The results show that the *c* cluster and the choice of distance have the greatest influence, while IR typically contributes very little to the variation in the indices.
