# Supplementary material for: A black-winged kite improved fuzzy clustering handling imbalanced uncertain data
Source: PLoS One. 2026 Jun 8;21(6):e0349753. doi: 10.1371/journal.pone.0349753 (PMC13245875; doi:10.1371/journal.pone.0349753)
Supplement: Attachment — Submitted filename: Response to Reviewers.pdf [file pone.0349753.s002.pdf]

Response to Reviewers
A Black-winged Kite Improved Fuzzy Clustering handling Imbalanced Uncertain Data

Hung Tran-Nam[1,2]◑, Ha Che-Ngoc[3]◑*

1 Laboratory for Applied and Industrial Mathematics, Institute for Computational Science and Artificial Intelligence, Van Lang University, Ho Chi Minh City, Vietnam (e-mail: hung.trannam@vlu.edu.vn)
2 Faculty of Basic Sciences, Van Lang University, Ho Chi Minh City, Vietnam
3 Applied Analysis Research Group, Faculty of Mathematics and Statistics, Ton Duc Thang University, Ho Chi Minh City, Vietnam (e-mail: chengocha@tdtu.edu.vn)

◑These authors contributed equally to this work.
* corresponding author: chengocha@tdtu.edu.vn

Dear Editor, Thank you for allowing a resubmission of our manuscript, with an opportunity to address the Reviewers' A detailed, point-by-point response to the Reviewers' comments is provided below.

Specifically, we have submitted (a) a point-by-point response to the comments (see below), (b) an updated manuscript with changes highlighted in yellow, and (c) a clean version of the revised manuscript. Manuscript with yellow highlighting indicating changes, and (c) a clean updated manuscript without highlights.

We trust that the revised manuscript satisfactorily addresses the Reviewers' requirements.

Best regards,

Ha Che-Ngoc.

Additional Editor Comments

This paper describes an improved fuzzy clustering algorithm to handle imbalanced uncertain data. Despite an interesting approach, the authors must highlight the novelty of the work by comparing it with other clustering algorithms. It is recommended to work on the Reviewer's comments for quality improvement.

Author Response: Thank you very much for the Reviewer comments. We appreciate your suggestion regarding the need to better highlight the novelty of our work. In the revised version, we will provide a more precise comparison with existing clustering algorithms and emphasise the contributions of the proposed method more explicitly. We will carefully address all reviewer comments to improve the overall quality of the manuscript.

Reviewer #1

The manuscript presents a novel two-stage clustering algorithm designed to handle imbalanced and uncertain data. The method combines optimisation with an enhanced clustering framework; the following are the main concerns regarding the proposed approach.

---

1. There is no comparison with deep clustering or modern ensemble methods that also handle imbalance and uncertainty. address

Authors' Response: Thank you for the Reviewer's comments. Because our research focuses on clustering methods for imbalanced and uncertain data represented by continuous distribution data, this type of data is characterised by rich distributional information; however, it requires substantial storage and is quite complicated to build deep clustering models. On the other hand, deep clustering methods rely on representation learning. They use deep neural networks to convert high-dimensional or unstructured data into a latent space where clustering becomes more discrete. These methods integrate feature learning and clustering, making them highly effective for images, signals, and other complex data types. Therefore, due to differences in data characteristics and incompatibility in feature representation, BKIFF and deep clustering operate in different feature spaces and follow different learning paradigms; a direct comparison between them would be technically inappropriate for our problem context.

However, we found one paper, with DOI: `http://doi.org/10.1007/s10618-025-01136-0`, that employs the relevant Methodology of the proposed method for extracting image features into modern convolutional architectures via Inception-ResNet-V2. Without this paper, we have not yet found a suitable algorithm for unbalanced data with respect to the probability density function. This is a new deep learning-based method that combines global average max pooling with the network to extract features for density function estimation and beam analysis evaluation. We also consider this a potential baseline and add it to the results of a cluster analysis comparison in the disequilibrium environment we are examining. The results show that although this method is considered promising and novel in terms of extraction, the cluster analysis algorithm proposed by Dinh Pham-Toan (2025) has not been improved, rendering it unable to handle imbalanced data effectively. Furthermore, Dinh's algorithm faces several significant challenges as it repeatedly calculates the pairwise distance matrix in phase one to find the optimal number of clusters and uses that label result to perform further fuzzy clustering with the proposed Kullback-Leibler distance. Here, the phases do not include awareness of imbalance handling, leading to biased and uneven results. Moreover, image extraction

via the pooling layer carries the risk of random bias as it does not contain any potentially optimised targets.

Authors' Action: We incorporated the clustering extraction and analysis method proposed by Dinh Pham-Toan (2025) as an additional baseline. We have added the configuration of this algorithm to Table 4 **(page 17/44)**, similar to other baseline algorithms. Because this is an automatic clustering algorithm, we do not need to set up a c-cluster. This method is included in Tables 11, 12, and 13 **(pages 23–24/44)** to facilitate a direct and transparent comparison with existing algorithms and the proposed BKIFF framework. In addition, we have added two dedicated paragraphs that discuss and critically evaluate the comparative performance of all methods, including Dinh Pham-Toan (2025), at **lines 539–576, pages 22–23/44**.

---

2. The convergence proof is provided; however, the exposition is dense and may be challenging for readers without a strong background in optimisation. Including a more intuitive explanation or a summary of the convergence properties would enhance accessibility.

Authors' Response: We thank the Reviewer for this observation. We acknowledge that including the complete derivations in the main text may disrupt the conceptual flow for readers unfamiliar with nonlinear optimisation. To address this, we have relocated the complete analytic proofs to the Supporting Information and provided a more intuitive summary in the revised manuscript.

Authors' Action: We have reorganised the convergence section. Specifically:

- All detailed proofs of the lemmas and theorems have been moved to the Supporting Information **lines 730–828, pages 30–34/44**. This includes two lemmas and four theorems.
- The main text now includes a concise and accessible explanation of the convergence mechanism. Specifically, the revised paragraph describes the monotonic decrease of the objective function and clarifies the contribution of each lemma and theorem to the overall convergence guarantee **(lines 270–283, pages 10–11/44)**.
- A new schematic diagram **(Figure 4, page 11/44)** has been added to summarise the logical structure of the convergence argument visually.

---

3. Parameter sensitivity analysis is essential for validating the robustness of the results.

Authors' Response: We sincerely thank the Reviewer for this important comment. Accordingly, a comprehensive sensitivity evaluation has now been incorporated into the revised manuscript.

Authors' Action: To address this comment thoroughly, the following additions and analyses have been made:

- Performed Wilcoxon signed-rank tests, and Friedman tests for all algorithms in the three Examples, including Tables of results.
- Added a new subsubsection "Sensitivity Analysis Setup" describing the experimental design, including the tested parameter groups including configurations $IR = \{20\,,50\,,80\,,100\}$, fuzziness coefficient $m = \{1.1\,,1.5\,,2.0\,,2.5\,,3.0\,,5.0\,,10.0\}$, the number of clusters $c = \{2\,,3\,,4\,,5\}$, and the type of distance metric $\{\mathcal{H}^2\,,\mathcal{L}_1\,,\mathcal{L}_2\,,CWD\}$. Each configuration has been repeated 10 times, and evaluation metrics including ARI, NMI, *Silhouette*, *Dunn* index, computation time, and iterations **(lines 436–455, page 17/44)**.

- Added a new subsection “Sensitivity Analysis of BKIFF”, presenting the interpretation of Morris and ANOVA results **(line 622–647, pages 28–29/44)**, identifying dominant and weak factors, and discussing stability across Examples, and summarised the results in a new Table 14 **(page 29/44)**.
- Provided heatmaps and pairwise configuration comparisons for all metrics, including *ARI*, *NMI*, *Dunn*, and *Silhouette* value across parameter settings in the Supplementary Information named “Pair configuations” **(lines 839–846, page 36/44)**.
- Ensured reproducibility by releasing all Octave code and sensitivity-testing scripts via a publicly accessible repository by link `https://doi.org/10.6084/m9.figshare.30600539.v4`

---

4. Some sentences contain minor grammatical errors.
Authors' Response: We thank the Reviewer for pointing out the grammatical issues in several sentences. We carefully reviewed the entire manuscript and corrected all minor grammatical and stylistic errors to improve clarity and readability.
Authors' Action: We revised the manuscript to fix grammatical inconsistencies, polished sentence structures, and improved overall language quality.

---

5. In some places, the acronym BKIFF is written inconsistently.
Authors' Response: We thank the Reviewer for pointing out the inconsistency in the use of the acronym. All occurrences have been carefully checked throughout the manuscript.
Authors' Action: We standardised the notation by replacing BKA with BKO where appropriate and corrected the full algorithm name in Algorithm 2 to “Black-winged Kite Improved Fuzzy Clustering for Probability Density Functions”.

---

6. Some figures (e.g., Fig. 2, Fig. 10) are not referenced in the article.
Authors' Response: We thank the Reviewer for pointing out the missing references.
Authors' Action: We have carefully reviewed the manuscript and added the appropriate in-text references for Figure 2 and Figure 10 at their relevant locations. All figures are now properly referenced and integrated into the narrative to ensure consistency and readability throughout the paper.

Reviewer #2

---

1. First of all, the paper is not well organised. Introduction and experimental work conclusions are not labelled with proper sections, which makes it ambiguous to read.
Authors' Response: Thank you for bringing this to my attention. To improve the clarity and organisation of the manuscript, we have revised the structure and ensured that all major sections are clearly labelled and consistently referenced.
Authors' Action: We have reorganised the remaining parts of the paper and added section labels with correct `\nameref` references **(lines 121–126, page 4/44)**. All cross-references throughout the manuscript have been checked and updated to ensure accuracy and consistency.

---

2. The problem statement and motivation of the method are not clear.

Authors' Response: We thank the Reviewer for their comment. We strengthened the articulation of why clustering probability density functions becomes highly unstable under imbalanced conditions and why a dedicated methodological solution is needed.

Authors' Action: Sseveral changes have been made:

- Clarified the problem statement by explicitly explaining how uncertainty combined with severe imbalance destabilises existing clustering methods and leads to prototype drift and minority-cluster loss **(lines 22–26, page 4/44).**
- Expanded the motivation through a focused related-work synthesis, highlighting recent optimisation-based or self-updating clustering approaches and explaining why none of them resolve the joint challenge of uncertainty and imbalance **(lines 45–52, page 3/44).**
- Made the research gap explicit by stating that, despite previous advances, no existing method provides a technical clustering framework tailored for imbalanced uncertain data, followed by a clear motivation for the necessity of such a method **(lines 80–82, page 4/44)**.

---

3. "Given the aforementioned gaps, this study introduces a novel approach call" – is written in two times at the introduction section

Authors' Response: We appreciate the Reviewer for pointing out the repetition of the phrase.

Authors' Action: We have removed the redundant occurrence of the sentence and revised the surrounding text.

---

4. The related works section is absent in this paper.

Authors' Response: Thank you for the Reviewer's observation. A related works section is indeed included in the manuscript **(lines 45–52, page 3/44)**. However, we acknowledge that its structure and presentation may not have clearly conveyed its role as a dedicated section. Therefore, we have revised and reorganised the content to ensure that the related works are more clearly articulated and properly positioned.

Authors' Action: The content of existing related works has been refined and reformatted to function as a distinct Related Works section. The revised version highlights prior studies more systematically and clarifies the positioning of our proposed method within the existing literature.

---

5. In Methodology, subsections are confusing because the Labels are not mentioned. Also, the motivation of each equation is not well defined.

Authors' Response: Yes. We have revised the Methodology section by adding clear subsection labels and explicitly explaining the motivation of each equations.

Authors' Action:

- A paragraph was added to articulate the motivation for the formulation of the IFF objective function **(Lines 165–168, page 6/44)**. This paragraph explains the dual purpose of minimising the Hellinger distance to cluster prototypes and mitigating the dominance of large clusters through cardinality-based weight factors.
- Included an explanation of the rationale behind the weight definition **(Lines 176–177 page 7/44)**, clarifying how the weighting mechanism ensures balanced influence across clusters of different sizes.

- Added a sentence motivating the necessity of an explicit initialisation step **(Lines 196–199, page 7/44)**, highlighting the idea explanation behind it.
- Inserted a clarification sentence explaining that the convergence analysis is organised as a sequence of lemmas and theorems following Zangwill's global convergence framework **(Lines 266–268, page 10/44)**.

---

6. The flow of Fig. 3 is not clear. Moreover, the framework of the proposed method is normally expected in the Methodology section.

Authors' Response: Thank you for the Reviewer's comment. We agree that the original flow of Figure 3 (**page 10/44**) is more appropriately placed in the Methodology section. We have revised the figure to provide a more transparent and more coherent flow, added explicit references to the corresponding subsections, and clarified the mathematical components to improve readability.

Authors' Action: The entire framework in Figure 3 (**page 10/44**) has been redesigned with a more transparent structure, labelled components, and more detailed formula descriptions. The updated figure has been moved to the Methodology section as suggested.

---

7. In the experimental section, the compared methods are quite old. There should be some new existing methods to compare performance.

Authors' Response: We thank the Reviewer for the valid comment. We have incorporated an additional contemporary baseline to ensure a more comprehensive and up-to-date evaluation.

Authors' Action: We have added the feature-extraction and clustering method proposed by Dinh Pham-Toan (2025) as a new baseline in Tables 11, 12, and 13 **(pages 23–24/44)**. This inclusion enables a direct and fair performance comparison between the proposed BKIFF method and other existing algorithms, including this recent approach.

---

8. The conclusion should be rewritten.

Authors' Response: We thank the Reviewer for their comment regarding the conclusion.

Authors' Action: We have completely rewritten the conclusion to provide a clearer, more coherent summary of the methodological contributions, experimental results, and practical implications of the proposed BKIFF algorithm **(lines 707–719, page 30/44)**.

Journal Requirements

---

1. Please ensure that your manuscript meets PLOS ONE's style requirements, including those for file naming. The PLOS ONE style templates can be found at `https://journals.plos.org/plosone/s/file?id=wjVg/PLOSOne_formatting_sample_main_body.pdf` and `https://journals.plos.org/plosone/s/file?id=ba62/PLOSOne_formatting_sample_title_authors_affiliations.pdf`

Authors' Response: We thank the Editor for this reminder.

Authors' Action: We have revised the manuscript to fully comply with PLOS ONE's style and formatting requirements, including file naming conventions. The manuscript has been prepared using the official PLOS ONE templates for the main body and for the title, authors, and affiliations, as provided at the referenced links.

---

2. PLOS requires an ORCID iD for the corresponding author in Editorial Manager on papers submitted after December 6th, 2016. Please ensure that you have an ORCID iD and that it is validated in Editorial Manager.

Authors' Response: We acknowledge the Editor's request regarding the ORCID iD requirement.

Authors' Action: The corresponding author's ORCID iD has been created and successfully validated in the Editorial Manager system using the Fetch/Validate function, in accordance with PLOS ONE's submission requirements.

---

3. We note that the grant information you provided in the 'Funding Information' and 'Financial Disclosure' sections does not match.

Authors' Response: We thank the Editor for bringing this inconsistency to our attention.

Authors' Action: We have carefully reviewed and corrected the grant information to ensure consistency between the Funding Information and Financial Disclosure sections. The correct grant numbers for all awards supporting this study have now been provided in the Funding Information section of the revised manuscript.

---

4. Thank you for stating the following financial disclosure: The Master's and PhD Scholarship Programme of Vingroup Innovation Foundation (VINIF) under Grant VINIF.2024.ThS.56

Please state what role the funders took in the study.

Authors' Response: We thank the Editor for requesting clarification regarding the role of the funder.

Authors' Action: We confirm that the funder had no role in study design, data collection and analysis, decision to publish, or preparation of the manuscript. This statement has been included in the cover letter as requested, so that the online submission form may be updated accordingly.

---

5. Thank you for stating the following in the Acknowledgements Section of your manuscript: The authors sincerely thank the Associate Editors and anonymous reviewers for their constructive comments, which improved the paper's version. We are also thankful because this research was funded by the Master's and PhD Scholarship Programme of Vingroup Innovation Foundation (VINIF) under Grant VINIF.2024.ThS.56. We note that you have provided funding information that is not currently declared in your Funding Statement. However, funding information should not appear in the Acknowledgements section or other areas of your manuscript. We will only

publish funding information present in the Funding Statement section of the online submission form. Please remove any funding-related text from the manuscript and let us know how you would like to update your Funding Statement. Currently, your Funding Statement reads as follows: ***The Master's and PhD Scholarship Programme of Vingroup Innovation Foundation (VINIF) under Grant VINIF.2024.ThS.56.*** **Please include your amended statements within your cover letter; we will change the online submission form on your behalf.**

Authors' Response: We thank the journal for clarifying the appropriate placement of funding information.

Authors' Action: We have addressed all issues according to your requirements.

6. Thank you for uploading your study's underlying data set. Unfortunately, the repository you have noted does not qualify as an acceptable data repository according to PLOS's standards.

Authors' Response: We thank the journal for this guidance regarding data availability requirements.

Authors' Action: Following PLOS ONE's policy, we have deposited the minimal data set and associated code necessary to replicate the study's findings in an approved public repository. The data and code are now available on Figshare at the following DOI: `https://doi.org/10.6084/m9.figshare.30600539.v4`

---

7. Please ensure that you refer to Figure 8 in your text.

Authors' Response: We thank the Editor for this observation.

Authors' Action: An explicit reference to Figure 8 has now been added at the appropriate location in the main text to ensure consistency between the text and figures.

---

8. If the reviewer comments include a recommendation to cite specific previously published works, please review and evaluate these publications.

Authors' Response: We thank the Editor for the valid comment.

Authors' Action: Although no specific citations were explicitly required, we have carefully re-examined the related literature and incorporated several additional relevant references to strengthen the background and discussion sections of the revised manuscript.
