# Supplementary material for: A black-winged kite improved fuzzy clustering handling imbalanced uncertain data
Source: PLoS One. 2026 Jun 8;21(6):e0349753. doi: 10.1371/journal.pone.0349753 (PMC13245875; doi:10.1371/journal.pone.0349753)
Supplement: Attachment — Submitted filename: Response_to_Reviewers_auresp_2.pdf [file pone.0349753.s003.pdf]

Response to Reviewers

A Black-winged Kite Improved Fuzzy Clustering handling Imbalanced Uncertain Data

Hung Tran-Nam[1,2◑], Ha Che-Ngoc[3◑*]

1 Laboratory for Applied and Industrial Mathematics, Institute for Computational Science and Artificial Intelligence, Van Lang University, Ho Chi Minh City, Vietnam (e-mail: hung.trannam@vlu.edu.vn)
2 Faculty of Basic Sciences, Van Lang University, Ho Chi Minh City, Vietnam
3 Applied Analysis Research Group, Faculty of Mathematics and Statistics, Ton Duc Thang University, Ho Chi Minh City, Vietnam (e-mail: chengocha@tdtu.edu.vn)

◑These authors contributed equally to this work.
* corresponding author: chengocha@tdtu.edu.vn

Dear Editor, Thank you for allowing us to resubmit our manuscript and for providing the opportunity to address the Reviewers' comments. A detailed, point-by-point response is provided below.

Specifically, we have submitted: (a) a point-by-point response to the comments, (b) a manuscript with changes highlighted in yellow, and (c) a clean version of the revised manuscript without highlights.

We trust that the revised manuscript satisfactorily addresses the Reviewers' requirements.

Best regards,

Ha Che-Ngoc.

Additional Editor Comments

The revised version has been evaluated for the accuracy of results and novelty. However, there are few areas which still require improvements. Moreover, particularly in the methodology and discussion sections, key innovations could be elaborated briefly for better comprehension.

Author's Response: We would like to express our sincere gratitude to the Editor and the Reviewer for this insightful observation. Additionally, we are pleased that our subsequent revisions have successfully addressed the previous concerns raised by the Reviewer.

Authors' Action: We have extensively revised the Methodology (**Section 3, pp. 16/44**) and Discussion (**Section 6, pp. 24/44**) to explicitly elaborate on the core innovations of the BKIFF framework. Specifically:

- In the Discussion section, we expanded our analysis to examine the interplay between these initialisation strategies and imbalance ratios, providing deeper insights into the algorithm's imbalanced-immunity threshold where baseline methods typically fail. This provides a clearer understanding of the algorithm's robustness (**Section 6, lines 653-666, pp. 24/44**).
- In the Methodology section, we added a new illustration (**Figure 9, pp. 16/44**) to clearly depict the proposed patch-based transformation pipeline. The figure visualises the process of generating local patches, applying a sliding window mechanism, unfolding each patch into column vectors, and subsequently performing kernel density estimation on these transformed representations. This addition improves the interpretability of the data transformation procedure and clarifies how the probabilistic structure is constructed for clustering.

Reviewer #1

Thanks the authors. All comments are answered properly and the article can be published in the present form.

Authors' Response: We are deeply grateful to the Reviewer for their thorough evaluation and for the favorable recommendation regarding the publication of our manuscript. We sincerely appreciate the time and expertise the Reviewer has dedicated to the review process.

Reviewer #3

1. The abstract reports very strong performance, including an ARI of 1.00 and lower execution times, but it does not provide enough quantitative comparison against specific baseline methods. Naming the main competing algorithms and indicating the magnitude of improvement would make the claims more convincing.

Authors' Response: We sincerely appreciate the Reviewer's constructive observation. We concur that the initial version of the abstract lacked sufficient detail to fully support the claims of superiority. Therefore, we have revised the abstract to clearly specify the main competing algorithms (FCF, FCF-$\mathcal{L}_1$, KMEANS, and Self-Updating) and to include the magnitude of improvements in terms of ARI, NMI, and computational time across different experimental settings.

Authors' Action: We have revised the abstract to explicitly describe the methodological novelty of the proposed framework. Specifically, we have updated the abstract to explicitly list the baseline methods used for comparison and report quantitative improvements. Specifically, the revised version now highlights that BKIFF achieves near-perfect ARI (up to 1.00), improves ARI by approximately 30–35% in moderately imbalanced cases and from near-zero in highly imbalanced scenarios, increases NMI by about 25–95%, and reduces computational time by approximately 95–99% compared to the baseline methods. These additions provide clearer and more convincing evidence of the superiority of the proposed approach.

---

2. Although the paper mentions theoretical convergence via Zangwill's theorem, the abstract does not clearly explain what is improved in the fuzzy clustering framework beyond the BKO-based initialization. A more explicit statement of the methodological novelty would help distinguish the proposed contribution from existing optimized fuzzy clustering methods.

Authors' Response: We thank the reviewer for this important comment. We have consequently revised the manuscript to explicitly delineate the methodological novelties of the BKIFF framework, thereby clearly distinguishing our contribution from conventional optimised improved fuzzy clustering approaches.

Authors' Action: We clarify that BKIFF incorporates the Hellinger distance into the clustering objective to better model similarities between probability density functions, and introduces improved membership updating and prototype estimation mechanisms through the proposed Improved Fuzzy clustering for probability density Functions (IFF), which is designed to effectively handle uncertainty and data imbalance.

---

3. The introduction should clearly conclude with a distinct section highlighting the novel contributions of your work.

Authors' Response: We sincerely appreciate the Reviewer's constructive suggestion. We concur that a dedicated section outlining the main contributions improves the clarity and structural flow of the Introduction.

Authors' Action: We have revised the Introduction to include a dedicated paragraph that explicitly summarises the main contributions of this study, specifically, Contributions (**SubSection 1.4, lines 115-150, pp. 5/44**)

---

4. At the ending of the intro, it is advised to add a para that mentions briefly what each next section contains.

Authors' Response: We sincerely thank the Reviewer for this constructive comment. Accordingly, we have revised the manuscript by adding a description of its structure.

Authors' Action: A paragraph summarizing the structure of the paper has been added at the end of the Introduction, specifically, Organisation (**SubSection 1.6, lines 151-158, pp. 5/44**)

---

5. The literature review should benefit from more explorations of previous studies.

Authors' Response: We sincerely appreciate the Reviewer's constructive assessment regarding the scope of the literature review. We have revised the Related Works section to include a more thorough exploration of prior studies.

Authors' Action: We have extensively revised and restructured the Related Works section to provide a more comprehensive literature review. Specifically:

- We have provided a more detailed description of representative methods such as Uncertain k-means, Uncertain k-medoids, kernel-based approaches, and

density-based methods (**lines 42-57, pp. 3/44**). Moreover, we have cited additional foundational articles, such as `https://doi.org/10.1007/s00778-005-0159-3`, and `https://doi.org/10.1016/j.neunet.2017.06.004`, for further analytical depth.

- We have explicitly analysed the underlying mechanisms and inherent limitations of these existing methods (**lines 60-74, pp. 3-4/44**).
- We have expanded the discussion on advances in recent algorithms based on evolutionary and adaptive strategies, such as genetic algorithms, differential evolution, and self-updating frameworks (**lines 79-81, pp. 4/44**).

---

6. The discussion section needs to be expanded to more thoroughly analyse the results.

Authors' Response: We sincerely thank the Reviewer's constructive recommendation.

Authors' Action: We have expanded the Discussion section (**Section 6, lines 653-666, pp. 24/44**) to provide a more thorough analysis of the results and explicitly analyse the interplay between initialisation strategies, imbalance ratios, and membership regularisation mechanisms.

---

7. The first paragraph of the conclusion should succinctly summarize the contributions of the study in past tense.

Authors' Response: Thank you for your constructive comment.

Authors' Action: The first paragraph of the Conclusion section has been carefully revised to succinctly summarize the key contributions of the study, with all descriptions consistently expressed in past tense (**lines 717-728, pp. 27/44**).

---

8. The second paragraph of the conclusion should provide clear and actionable future recommendations.

Authors' Response: Thank you for your valuable comment. In the original manuscript, these recommendations were presented in a dedicated Future Work subsection within the Discussion.

Authors' Action: We have moved and incorporated the key future research directions into the second paragraph of Conclusion (**lines 730-739, pp. 27-28/44**). Specific recommendations include integrating deep clustering, expanding to contrastive learning for images/video, and applying the density framework to areas such as solar radiation analysis or species distribution modeling. Additionally, we cited two article `https://doi.org/10.1371/journal.pone.0317396` which involve the clustering-based SMOTE technique to handle imbalanced data and `https://doi.org/10.1371/journal.pone.0340758` on evolutionary undersampling to detect rare events in complex data environments.

---

9. Equations are not properly cited, please add original references.

Authors' Response: We sincerely thank the reviewers for their valuable comments. We have reviewed the entire manuscript and added citations to the Hellinger Distance, Dunn Index, and Silhouette Coefficient.

Authors' Action: We have made the following specific updates to the revised manuscript, including

- We added Hellinger (1909) `https://doi.org/10.1515/crll.1909.136.210` in (**Definition 3, pp. 6/44**) to acknowledge the theoretical origin of this measure,

- We added Dunn (1973) `https://doi.org/10.1080/01969727308546046` (**Section 3.6.3, pp. 13/44**) when defining the standard for evaluating the distance between clusters,

- We added Rousseeuw (1987) `https://doi.org/10.1016/0377-0427(87)90125-7` (**Section 3.6.4, pp. 13/44**) to confirm the origin of the method of measuring cluster separation.

---

10. Why are the sections not numbered? this gives a hard time for the reviewers to do their job, please number each section and each subsection of the article in the second round of review.

Authors' Response: We sincerely appreciate the Reviewer's constructive observation regarding the manuscript's structural organisation.

Authors' Action: All sections and subsections have been numbered and highlighted in the revised manuscript.
