# Supplementary material for: A black-winged kite improved fuzzy clustering handling imbalanced uncertain data
Source: PLoS One. 2026 Jun 8;21(6):e0349753. doi: 10.1371/journal.pone.0349753 (PMC13245875; doi:10.1371/journal.pone.0349753)
Supplement: Peer review history

# Peer review: Decision Letter 0

**DOI:** 10.1371/journal.pone.0349753.r001

**Authors:** Zeashan Khan

12 Nov 2025

PONE-D-25-57245

A Black-winged Kite Improved Fuzzy Clustering handling Imbalanced Uncertain Data

PLOS ONE

Dear Dr. Che Ngoc,

Thank you for submitting your manuscript to PLOS ONE. After careful consideration, we feel that it has merit but does not fully meet PLOS ONE’s publication criteria as it currently stands. Therefore, we invite you to submit a revised version of the manuscript that addresses the points raised during the review process.

Please submit your revised manuscript by Dec 27 2025 11:59PM. If you will need more time than this to complete your revisions, please reply to this message or contact the journal office at plosone@plos.org. When you're ready to submit your revision, log on to https://www.editorialmanager.com/pone/ and select the 'Submissions Needing Revision' folder to locate your manuscript file.

Please include the following items when submitting your revised manuscript:

- A rebuttal letter that responds to each point raised by the academic editor and reviewer(s). You should upload this letter as a separate file labeled 'Response to Reviewers'.
- A marked-up copy of your manuscript that highlights changes made to the original version. You should upload this as a separate file labeled 'Revised Manuscript with Track Changes'.
- An unmarked version of your revised paper without tracked changes. You should upload this as a separate file labeled 'Manuscript'.

If you would like to make changes to your financial disclosure, please include your updated statement in your cover letter. Guidelines for resubmitting your figure files are available below the reviewer comments at the end of this letter.

If applicable, we recommend that you deposit your laboratory protocols in protocols.io to enhance the reproducibility of your results. Protocols.io assigns your protocol its own identifier (DOI) so that it can be cited independently in the future. For instructions see: https://journals.plos.org/plosone/s/submission-guidelines#loc-laboratory-protocols. Additionally, PLOS ONE offers an option for publishing peer-reviewed Lab Protocol articles, which describe protocols hosted on protocols.io. Read more information on sharing protocols at https://plos.org/protocols?utm_medium=editorial-email&utm_source=authorletters&utm_campaign=protocols.

We look forward to receiving your revised manuscript.

Kind regards,

Zeashan Hameed Khan, Ph.D.

Academic Editor

PLOS ONE

Journal Requirements:

When submitting your revision, we need you to address these additional requirements.

1. Please ensure that your manuscript meets PLOS ONE's style requirements, including those for file naming. The PLOS ONE style templates can be found at https://journals.plos.org/plosone/s/file?id=wjVg/PLOSOne_formatting_sample_main_body.pdf and https://journals.plos.org/plosone/s/file?id=ba62/PLOSOne_formatting_sample_title_authors_affiliations.pdf

2. PLOS requires an ORCID iD for the corresponding author in Editorial Manager on papers submitted after December 6th, 2016. Please ensure that you have an ORCID iD and that it is validated in Editorial Manager. To do this, go to ‘Update my Information’ (in the upper left-hand corner of the main menu), and click on the Fetch/Validate link next to the ORCID field. This will take you to the ORCID site and allow you to create a new iD or authenticate a pre-existing iD in Editorial Manager.

3. We note that the grant information you provided in the ‘Funding Information’ and ‘Financial Disclosure’ sections do not match.

When you resubmit, please ensure that you provide the correct grant numbers for the awards you received for your study in the ‘Funding Information’ section.

4. Thank you for stating the following financial disclosure:

The Master’s and PhD Scholarship Programme of 780 Vingroup Innovation Foundation (VINIF) under Grant VINIF.2024.ThS.56

Please state what role the funders took in the study. If the funders had no role, please state: "The funders had no role in study design, data collection and analysis, decision to publish, or preparation of the manuscript."

If this statement is not correct you must amend it as needed.

Please include this amended Role of Funder statement in your cover letter; we will change the online submission form on your behalf.

5. Thank you for stating the following in the Acknowledgments Section of your manuscript:

The authors sincerely thank the Associate Editors and anonymous reviewers for their constructive comments, which improved the paper’s version. We are also thankful because this research was funded by the Master’s and PhD Scholarship Programme of Vingroup Innovation Foundation (VINIF) under Grant VINIF.2024.ThS.56.

We note that you have provided funding information that is not currently declared in your Funding Statement. However, funding information should not appear in the Acknowledgments section or other areas of your manuscript. We will only publish funding information present in the Funding Statement section of the online submission form.

Please remove any funding-related text from the manuscript and let us know how you would like to update your Funding Statement. Currently, your Funding Statement reads as follows:

The Master’s and PhD Scholarship Programme of 780 Vingroup Innovation Foundation (VINIF) under Grant VINIF.2024.ThS.56

Please include your amended statements within your cover letter; we will change the online submission form on your behalf.

6. Thank you for uploading your study's underlying data set. Unfortunately, the repository you have noted in your Data Availability statement does not qualify as an acceptable data repository according to PLOS's standards.

At this time, please upload the minimal data set necessary to replicate your study's findings to a stable, public repository (such as figshare or Dryad) and provide us with the relevant URLs, DOIs, or accession numbers that may be used to access these data. For a list of recommended repositories and additional information on PLOS standards for data deposition, please see https://journals.plos.org/plosone/s/recommended-repositories.

7. Please ensure that you refer to Figure 8 in your text as, if accepted, production will need this reference to link the reader to the figure.

8. If the reviewer comments include a recommendation to cite specific previously published works, please review and evaluate these publications to determine whether they are relevant and should be cited. There is no requirement to cite these works unless the editor has indicated otherwise.

Additional Editor Comments:

This paper describes an improved fuzzy clustering algorithm to handle imbalanced uncertain data. Despite an interesting approach, the authors must highlight the novelty of the work by comparing with other clustering algorithms. It is recommended to work on the reviewer's comments for quality improvement.

[Note: HTML markup is below. Please do not edit.]

Reviewers' comments:

Reviewer's Responses to Questions

**Comments to the Author**

1. Is the manuscript technically sound, and do the data support the conclusions?

The manuscript must describe a technically sound piece of scientific research with data that supports the conclusions. Experiments must have been conducted rigorously, with appropriate controls, replication, and sample sizes. The conclusions must be drawn appropriately based on the data presented.

Reviewer #1: Yes

Reviewer #2: Partly

**********

2. Has the statistical analysis been performed appropriately and rigorously?

Reviewer #1: Yes

Reviewer #2: Yes

**********

3. Have the authors made all data underlying the findings in their manuscript fully available?

The PLOS Data policy requires authors to make all data underlying the findings described in their manuscript fully available without restriction, with rare exception (please refer to the Data Availability Statement in the manuscript PDF file). The data should be provided as part of the manuscript or its supporting information, or deposited to a public repository. For example, in addition to summary statistics, the data points behind means, medians and variance measures should be available. If there are restrictions on publicly sharing data—e.g. participant privacy or use of data from a third party—those must be specified.

Reviewer #1: Yes

Reviewer #2: Yes

**********

4. Is the manuscript presented in an intelligible fashion and written in standard English?

PLOS ONE does not copyedit accepted manuscripts, so the language in submitted articles must be clear, correct, and unambiguous. Any typographical or grammatical errors should be corrected at revision, so please note any specific errors here.

Reviewer #1: Yes

Reviewer #2: Yes

**********

5. Review Comments to the Author

Please use the space provided to explain your answers to the questions above. You may also include additional comments for the author, including concerns about dual publication, research ethics, or publication ethics. (Please upload your review as an attachment if it exceeds 20,000 characters)

Reviewer #1: The manuscript presents a novel two-stage clustering algorithm designed to handle imbalanced and uncertain data. The method combines an optimization with an improved enhanced clustering framework. These The following the main concerns with regarding proposed approach.

1. There is no comparison with deep clustering or modern ensemble methods that also handle imbalance and uncertainty. address

2. The convergence proof is provided; however, the exposition is dense and may be challenging for readers without a strong background in optimization. Including a more intuitive explanation or a summary of the convergence properties would enhance accessibility.

3. Parameter sensitivity analysis is essential for validating the robustness of the results.

4. Some sentences contain minor grammatical errors.

5. In some places, the acronym BKIFF is written inconsistently.

6. Some figures (e.g., Fig. 2, Fig. 10) are not referenced in the article.

Reviewer #2: 1. First of all, the paper is not well organized. Introduction, experimental work conclusion are not Labeled with proper sections which makes ambiguous to read.

2. Problem statement and motivation of the method are not clear.

3. “Given the aforementioned gaps, this study introduces a novel approach call “ – is written in two time at introduction section

4. Related works section is absence in this paper.

5. In Methodology, subsections are confusing due to Labels are not mentioned. Also, motivation of each equation is not well defined.

6. Flow of the Fig. 3 is not clear. Moreover, the framework of the proposed method is normally expected at the Methodology section.

7. In experimental section, compared methods are quite older. There should be some new existing methods to compare performance.

8. Conclusion should be rewritten.

**********

6. PLOS authors have the option to publish the peer review history of their article (what does this mean?). If published, this will include your full peer review and any attached files.

If you choose “no”, your identity will remain anonymous but your review may still be made public.

**Do you want your identity to be public for this peer review?** For information about this choice, including consent withdrawal, please see our Privacy Policy.

Reviewer #1: **Yes:** Mohammad Hossein Moattar

Reviewer #2: No

**********

[NOTE: If reviewer comments were submitted as an attachment file, they will be attached to this email and accessible via the submission site. Please log into your account, locate the manuscript record, and check for the action link "View Attachments". If this link does not appear, there are no attachment files.]

To ensure your figures meet our technical requirements, please review our figure guidelines: https://journals.plos.org/plosone/s/figures

You may also use PLOS’s free figure tool, NAAS, to help you prepare publication quality figures: https://journals.plos.org/plosone/s/figures#loc-tools-for-figure-preparation.

NAAS will assess whether your figures meet our technical requirements by comparing each figure against our figure specifications.

# Author comment: Author response to Decision Letter 1

**DOI:** 10.1371/journal.pone.0349753.r002

2 Jan 2026

I. Additional Editor Comments

This paper describes an improved fuzzy clustering algorithm to handle imbalanced uncertain data. Despite an interesting approach, the authors must highlight the novelty of the work by comparing it with other clustering algorithms. It is recommended to work on the Reviewer's comments for quality improvement.

Author Response: Thank you very much for the Reviewer comments. We appreciate your suggestion regarding the need to better highlight the novelty of our work. In the revised version, we will provide a more precise comparison with existing clustering algorithms and emphasise the contributions of the proposed method more explicitly. We will carefully address all reviewer comments to improve the overall quality of the manuscript.

II. Reviewer 1

The manuscript presents a novel two-stage clustering algorithm designed to handle imbalanced and uncertain data. The method combines optimisation with an enhanced clustering framework; the following are the main concerns regarding the proposed approach.

1. There is no comparison with deep clustering or modern ensemble methods that also handle imbalance and uncertainty. address

Authors' Response: Thank you for the Reviewer's comments. Because our research focuses on clustering methods for imbalanced and uncertain data represented by continuous distribution data, this type of data is characterised by rich distributional information; however, it requires substantial storage and is quite complicated to build deep clustering models. On the other hand, deep clustering methods rely on representation learning. They use deep neural networks to convert high-dimensional or unstructured data into a latent space where clustering becomes more discrete. These methods integrate feature learning and clustering, making them highly effective for images, signals, and other complex data types. Therefore, due to differences in data characteristics and incompatibility in feature representation, BKIFF and deep clustering operate in different feature spaces and follow different learning paradigms; a direct comparison between them would be technically inappropriate for our problem context.

However, we found one paper, with DOI: http://doi.org/10.1007/s10618-025-01136-0, that employs the relevant Methodology of the proposed method for extracting image features into modern convolutional architectures via Inception-ResNet-V2. Without this paper, we have not yet found a suitable algorithm for unbalanced data with respect to the probability density function. This is a new deep learning-based method that combines global average max pooling with the network to extract features for density function estimation and beam analysis evaluation. We also consider this a potential baseline and add it to the results of a cluster analysis comparison in the disequilibrium environment we are examining. The results show that although this method is considered promising and novel in terms of extraction, the cluster analysis algorithm proposed by Dinh Pham-Toan (2025) has not been improved, rendering it unable to handle imbalanced data effectively. Furthermore, Dinh's algorithm faces several significant challenges as it repeatedly calculates the pairwise distance matrix in phase one to find the optimal number of clusters and uses that label result to perform further fuzzy clustering with the proposed Kullback-Leibler distance. Here, the phases do not include awareness of imbalance handling, leading to biased and uneven results. Moreover, image extraction via the pooling layer carries the risk of random bias as it does not contain any potentially optimised targets.

Authors' Action: We incorporated the clustering extraction and analysis method proposed by Dinh Pham-Toan (2025) as an additional baseline. We have added the configuration of this algorithm to Table 4 (page 17/44), similar to other baseline algorithms. Because this is an automatic clustering algorithm, we do not need to set up a c-cluster. This method is included in Tables 11, 12, and 13 (pages 23--24/44) to facilitate a direct and transparent comparison with existing algorithms and the proposed BKIFF framework. In addition, we have added two dedicated paragraphs that discuss and critically evaluate the comparative performance of all methods, including Dinh Pham-Toan (2025), at lines 539--576, pages 22--23/44.

2. The convergence proof is provided; however, the exposition is dense and may be challenging for readers without a strong background in optimisation. Including a more intuitive explanation or a summary of the convergence properties would enhance accessibility.

Authors' Response: We thank the Reviewer for this observation. We acknowledge that including the complete derivations in the main text may disrupt the conceptual flow for readers unfamiliar with nonlinear optimisation. To address this, we have relocated the complete analytic proofs to the Supporting Information and provided a more intuitive summary in the revised manuscript.

Authors' Action: We have reorganised the convergence section. Specifically:

- All detailed proofs of the lemmas and theorems have been moved to the Supporting Information lines 730--828, pages 30--34/44. This includes two lemmas and four theorems.

- The main text now includes a concise and accessible explanation of the convergence mechanism. Specifically, the revised paragraph describes the monotonic decrease of the objective function and clarifies the contribution of each lemma and theorem to the overall convergence guarantee (lines 270--283, pages 10--11/44).

- A new schematic diagram (Figure~4, page 11/44) has been added to summarise the logical structure of the convergence argument visually.

3. Parameter sensitivity analysis is essential for validating the robustness of the results.

Authors' Response: We sincerely thank the Reviewer for this important comment. Accordingly, a comprehensive sensitivity evaluation has now been incorporated into the revised manuscript.

Authors' Action: To address this comment thoroughly, the following additions and analyses have been made:

- Performed Wilcoxon signed-rank tests, and Friedman tests for all algorithms in the three Examples, including Tables of results.

- Added a new subsubsection "Sensitivity Analysis Setup" describing the experimental design, including the tested parameter groups including configurations $IR=\{20\,,50\,,80\,,100\}$, fuzziness coefficient $m=\{1.1\,,1.5\,,2.0\,,2.5\,,3.0\,,5.0\,,10.0\}$, the number of clusters $c=\{2\,,3\,,4\,,5\}$, and the type of distance metric $\{\mathcal{H}^2\,,\mathcal{L}_1\,,\mathcal{L}_2\,,CWD\}$. Each configuration has been repeated 10 times, and evaluation metrics including $ARI$, $NMI$, $Silhouette$, $Dunn$ index, computation time, and iterations (lines 436--455, page 17/44).

- Added a new subsection "Sensitivity Analysis of BKIFF", presenting the interpretation of Morris and ANOVA results (line 622--647, pages 28--29/44), identifying dominant and weak factors, and discussing stability across Examples, and summarised the results in a new Table~14 (page 29/44).

- Provided heatmaps and pairwise configuration comparisons for all metrics, including $ARI$, $NMI$, $Dunn$, and $ Silhouette$ value across parameter settings in the Supplementary Information named ``Pair configuations'' \textbf{(lines 839--846, page 36/44)}.

- Ensured reproducibility by releasing all Octave code and sensitivity-testing scripts via a publicly accessible repository by link https://doi.org/10.6084/m9.figshare.30600539.v4

4. Some sentences contain minor grammatical errors.

Authors' Response: We thank the Reviewer for pointing out the grammatical issues in several sentences. We carefully reviewed the entire manuscript and corrected all minor grammatical and stylistic errors to improve clarity and readability.

Authors' Action: We revised the manuscript to fix grammatical inconsistencies, polished sentence structures, and improved overall language quality.

5. In some places, the acronym BKIFF is written inconsistently.

Authors' Response: We thank the Reviewer for pointing out the inconsistency in the use of the acronym. All occurrences have been carefully checked throughout the manuscript.

Authors' Action: We standardised the notation by replacing BKA with BKO where appropriate and corrected the full algorithm name in Algorithm 2 to ``Black-winged Kite Improved Fuzzy Clustering for Probability Density Functions".

6. Some figures (e.g., Fig. 2, Fig. 10) are not referenced in the article.

Authors' Response: We thank the Reviewer for pointing out the missing references.

Authors' Action: We have carefully reviewed the manuscript and added the appropriate in-text references for Figure 2 and Figure 10 at their relevant locations. All figures are now properly referenced and integrated into the narrative to ensure consistency and readability throughout the paper.

III. Reviewer 2

1. First of all, the paper is not well organised. Introduction and experimental work conclusions are not labelled with proper sections, which makes it ambiguous to read.

Authors' Response: Thank you for bringing this to my attention. To improve the clarity and organisation of the manuscript, we have revised the structure and ensured that all major sections are clearly labelled and consistently referenced.

Authors' Action: We have reorganised the remaining parts of the paper and added section labels with correct \verb|\nameref| references (lines 121--126, page 4/44). All cross-references throughout the manuscript have been checked and updated to ensure accuracy and consistency.

2. The problem statement and motivation of the method are not clear.

Authors' Response: We thank the Reviewer for their comment. We strengthened the articulation of why clustering probability density functions becomes highly unstable under imbalanced conditions and why a dedicated methodological solution is needed.

Authors' Action: Sseveral changes have been made:

- Clarified the problem statement by explicitly explaining how uncertainty combined with severe imbalance destabilises existing clustering methods and leads to prototype drift and minority-cluster loss (lines 22--26, page 4/44).

- Expanded the motivation through a focused related-work synthesis, highlighting recent optimisation-based or self-updating clustering approaches and explaining why none of them resolve the joint challenge of uncertainty and imbalance (lines 45--52, page 3/44).

- Made the research gap explicit by stating that, despite previous advances, no existing method provides a technical clustering framework tailored for imbalanced uncertain data, followed by a clear motivation for the necessity of such a method (lines 80--82, page 4/44).

3. "Given the aforementioned gaps, this study introduces a novel approach call" – is written in two times at the introduction section

Authors' Response: We appreciate the Reviewer for pointing out the repetition of the phrase.

Authors' Action: We have removed the redundant occurrence of the sentence and revised the surrounding text.

4. The related works section is absent in this paper.

Authors' Response: Thank you for the Reviewer's observation. A related works section is indeed included in the manuscript (lines 45--52, page 3/44). However, we acknowledge that its structure and presentation may not have clearly conveyed its role as a dedicated section. Therefore, we have revised and reorganised the content to ensure that the related works are more clearly articulated and properly positioned.

Authors' Action: The content of existing related works has been refined and reformatted to function as a distinct Related Works section. The revised version highlights prior studies more systematically and clarifies the positioning of our proposed method within the existing literature.

5. In Methodology, subsections are confusing because the Labels are not mentioned. Also, the motivation of each equation is not well defined.

Authors' Response: Yes. We have revised the Methodology section by adding clear subsection labels and explicitly explaining the motivation of each equations.

Authors' Action:

- A paragraph was added to articulate the motivation for the formulation of the IFF objective function (Lines 165--168, page 6/44). This paragraph explains the dual purpose of minimising the Hellinger distance to cluster prototypes and mitigating the dominance of large clusters through cardinality-based weight factors.

- Included an explanation of the rationale behind the weight definition (Lines 176--177 page 7/44), clarifying how the weighting mechanism ensures balanced influence across clusters of different sizes.

- Added a sentence motivating the necessity of an explicit initialisation step (Lines 196--199, page 7/44), highlighting the idea explanation behind it.

- Inserted a clarification sentence explaining that the convergence analysis is organised as a sequence of lemmas and theorems following Zangwill's global convergence framework (Lines 266--268, page 10/44).

6. The flow of Fig. 3 is not clear. Moreover, the framework of the proposed method is normally expected in the Methodology section.

Authors' Response: Thank you for the Reviewer's comment. We agree that the original flow of Figure 3 (page 10/44) is more appropriately placed in the Methodology section. We have revised the figure to provide a more transparent and more coherent flow, added explicit references to the corresponding subsections, and clarified the mathematical components to improve readability.

Authors' Action: The entire framework in Figure 3 (page 10/44) has been redesigned with a more transparent structure, labelled components, and more detailed formula descriptions. The updated figure has been moved to the Methodology section as suggested.

7. In the experimental section, the compared methods are quite old. There should be some new existing methods to compare performance.

Authors' Response: We thank the Reviewer for the valid comment. We have incorporated an additional contemporary baseline to ensure a more comprehensive and up-to-date evaluation.

Authors' Action: We have added the feature-extraction and clustering method proposed by Dinh Pham-Toan (2025) as a new baseline in Tables 11, 12, and 13 (pages 23--24/44). This inclusion enables a direct and fair performance comparison between the proposed BKIFF method and other existing algorithms, including this recent approach.

8. The conclusion should be rewritten.

Authors' Response: We thank the Reviewer for their comment regarding the conclusion.

Authors' Action: We have completely rewritten the conclusion to provide a clearer, more coherent summary of the methodological contributions, experimental results, and practical implications of the proposed BKIFF algorithm (lines 707--719, page 30/44).

IV. Journal Requirements

1. Please ensure that your manuscript meets PLOS ONE's style requirements, including those for file naming. The PLOS ONE style templates can be found at https://journals.plos.org/plosone/s/file?id=wjVg/PLOSOne_formatting_sample_main_body.pdf and https://journals.plos.org/plosone/s/file?id=ba62/PLOSOne_formatting_sample_title_authors_affiliations.pdf

Authors' Response: We thank the Editor for this reminder.

Authors' Action: We have revised the manuscript to fully comply with PLOS ONE's style and formatting requirements, including file naming conventions. The manuscript has been prepared using the official PLOS ONE templates for the main body and for the title, authors, and affiliations, as provided at the referenced links.

2. PLOS requires an ORCID iD for the corresponding author in Editorial Manager on papers submitted after December 6th, 2016. Please ensure that you have an ORCID iD and that it is validated in Editorial Manager.

Authors' Response: We acknowledge the Editor's request regarding the ORCID iD requirement.

Authors' Action: The corresponding author's ORCID iD has been created and successfully validated in the Editorial Manager system using the Fetch/Validate function, in accordance with PLOS ONE's submission requirements.

3. We note that the grant information you provided in the 'Funding Information' and 'Financial

Attachment Submitted filename: Response to Reviewers.pdf

# Peer review: Decision Letter 1

**DOI:** 10.1371/journal.pone.0349753.r003

**Authors:** Zeashan Khan

18 Mar 2026

PONE-D-25-57245R1

A Black-winged Kite Improved Fuzzy Clustering handling Imbalanced Uncertain Data

PLOS One

Dear Dr. Che Ngoc,

Thank you for submitting your manuscript to PLOS ONE. After careful consideration, we feel that it has merit but does not fully meet PLOS ONE’s publication criteria as it currently stands. Therefore, we invite you to submit a revised version of the manuscript that addresses the points raised during the review process.

Please submit your revised manuscript by May 02 2026 11:59PM. If you will need more time than this to complete your revisions, please reply to this message or contact the journal office at plosone@plos.org. When you're ready to submit your revision, log on to https://www.editorialmanager.com/pone/ and select the 'Submissions Needing Revision' folder to locate your manuscript file.

Please include the following items when submitting your revised manuscript:

- A letter that responds to each point raised by the academic editor and reviewer(s). You should upload this letter as a separate file labeled 'Response to Reviewers'.
- A marked-up copy of your manuscript that highlights changes made to the original version. You should upload this as a separate file labeled 'Revised Manuscript with Track Changes'.
- An unmarked version of your revised paper without tracked changes. You should upload this as a separate file labeled 'Manuscript'.

-->If you would like to make changes to your financial disclosure, please include your updated statement in your cover letter. Guidelines for resubmitting your figure files are available below the reviewer comments at the end of this letter.

If applicable, we recommend that you deposit your laboratory protocols in protocols.io to enhance the reproducibility of your results. Protocols.io assigns your protocol its own identifier (DOI) so that it can be cited independently in the future. For instructions see: https://journals.plos.org/plosone/s/submission-guidelines#loc-laboratory-protocols. Additionally, PLOS ONE offers an option for publishing peer-reviewed Lab Protocol articles, which describe protocols hosted on protocols.io. Read more information on sharing protocols at https://plos.org/protocols?utm_medium=editorial-email&utm_source=authorletters&utm_campaign=protocols.

We look forward to receiving your revised manuscript.

Kind regards,

Zeashan Hameed Khan, Ph.D.

Academic Editor

PLOS One

Journal Requirements:

If the reviewer comments include a recommendation to cite specific previously published works, please review and evaluate these publications to determine whether they are relevant and should be cited. There is no requirement to cite these works unless the editor has indicated otherwise.

Please review your reference list to ensure that it is complete and correct. If you have cited papers that have been retracted, please include the rationale for doing so in the manuscript text, or remove these references and replace them with relevant current references. Any changes to the reference list should be mentioned in the rebuttal letter that accompanies your revised manuscript. If you need to cite a retracted article, indicate the article’s retracted status in the References list and also include a citation and full reference for the retraction notice.

Additional Editor Comments (if provided):

The revised version has been evaluated for the accuracy of results and novelty. However, there are few areas which still require improvements. Moreover, particularly in the methodology and discussion sections, key innovations could be elaborated briefly for better comprehension.

[Note: HTML markup is below. Please do not edit.]

Reviewers' comments:

Reviewer's Responses to Questions

**Comments to the Author**

1. If the authors have adequately addressed your comments raised in a previous round of review and you feel that this manuscript is now acceptable for publication, you may indicate that here to bypass the “Comments to the Author” section, enter your conflict of interest statement in the “Confidential to Editor” section, and submit your "Accept" recommendation.

Reviewer #1: All comments have been addressed

Reviewer #3: (No Response)

**********

2. Is the manuscript technically sound, and do the data support the conclusions?

The manuscript must describe a technically sound piece of scientific research with data that supports the conclusions. Experiments must have been conducted rigorously, with appropriate controls, replication, and sample sizes. The conclusions must be drawn appropriately based on the data presented.

Reviewer #1: Yes

Reviewer #3: Yes

**********

3. Has the statistical analysis been performed appropriately and rigorously?

Reviewer #1: Yes

Reviewer #3: Yes

**********

4. Have the authors made all data underlying the findings in their manuscript fully available?

The PLOS Data policy requires authors to make all data underlying the findings described in their manuscript fully available without restriction, with rare exception (please refer to the Data Availability Statement in the manuscript PDF file). The data should be provided as part of the manuscript or its supporting information, or deposited to a public repository. For example, in addition to summary statistics, the data points behind means, medians and variance measures should be available. If there are restrictions on publicly sharing data—e.g. participant privacy or use of data from a third party—those must be specified.

Reviewer #1: No

Reviewer #3: No

**********

5. Is the manuscript presented in an intelligible fashion and written in standard English?

PLOS ONE does not copyedit accepted manuscripts, so the language in submitted articles must be clear, correct, and unambiguous. Any typographical or grammatical errors should be corrected at revision, so please note any specific errors here.

Reviewer #1: Yes

Reviewer #3: Yes

**********

6. Review Comments to the Author

Please use the space provided to explain your answers to the questions above. You may also include additional comments for the author, including concerns about dual publication, research ethics, or publication ethics. (Please upload your review as an attachment if it exceeds 20,000 characters)

Reviewer #1: Thanks the authors. All comments are answered properly and the article can be published in the present form

Reviewer #3: 1. The abstract reports very strong performance, including an ARI of 1.00 and lower execution times, but it does not provide enough quantitative comparison against specific baseline methods. Naming the main competing algorithms and indicating the magnitude of improvement would make the claims more convincing.

2. Although the paper mentions theoretical convergence via Zangwill’s theorem, the abstract does not clearly explain what is improved in the fuzzy clustering framework beyond the BKO-based initialization. A more explicit statement of the methodological novelty would help distinguish the proposed contribution from existing optimized fuzzy clustering methods.

3. The introduction should clearly conclude with a distinct section highlighting the novel contributions of your work.

4. At the ending of the intro, it is advised to add a para that mentions briefly what each next section contains.

5. The literature review should benefit from more explorations of previous studies.

6. The discussion section needs to be expanded to more thoroughly analyze the results.

7. The first paragraph of the conclusion should succinctly summarize the contributions of the study in past tense.

8. The second paragraph of the conclusion should provide clear and actionable future recommendations.

9. Equations are not properly cited, please add original references.

10. Why are the sections not numbered? this gives a hard time for the reviewers to do their job, please number each section and each subsection of the article in the second round of review.

**********

7. PLOS authors have the option to publish the peer review history of their article (what does this mean?). If published, this will include your full peer review and any attached files.

If you choose “no”, your identity will remain anonymous but your review may still be made public.

**Do you want your identity to be public for this peer review?** For information about this choice, including consent withdrawal, please see our Privacy Policy.

Reviewer #1: No

Reviewer #3: **Yes:** Luttfi A. Al-Haddad

**********

[NOTE: If reviewer comments were submitted as an attachment file, they will be attached to this email and accessible via the submission site. Please log into your account, locate the manuscript record, and check for the action link "View Attachments". If this link does not appear, there are no attachment files.]

To ensure your figures meet our technical requirements, please review our figure guidelines: https://journals.plos.org/plosone/s/figures

You may also use PLOS’s free figure tool, NAAS, to help you prepare publication quality figures: https://journals.plos.org/plosone/s/figures#loc-tools-for-figure-preparation.

NAAS will assess whether your figures meet our technical requirements by comparing each figure against our figure specifications.

# Author comment: Author response to Decision Letter 2

**DOI:** 10.1371/journal.pone.0349753.r004

30 Mar 2026

- Additional Editor Comments

The revised version has been evaluated for the accuracy of results and novelty. However, there are few areas which still require improvements. Moreover, particularly in the methodology and discussion sections, key innovations could be elaborated briefly for better comprehension.

- Author’s Response: We would like to express our sincere gratitude to the Editor and the Reviewer for this insightful observation. Additionally, we are pleased that our subsequent revisions have successfully addressed the previous concerns raised by the Reviewer.

- Authors’ Action: We have extensively revised the Methodology (Section 3, pp. 16/44) and Discussion (Section 6, pp. 24/44) to explicitly elaborate on the core innovations of the BKIFF framework. Specifically:

• In the Discussion section, we expanded our analysis to examine the interplay between these initialisation strategies and imbalance ratios, providing deeper insights into the algorithm’s imbalanced-immunity threshold where baseline methods typically fail. This provides a clearer understanding of the algorithm’s robustness (Section 6, lines 653-666, pp. 24/44).

• In the Methodology section, we added a new illustration (Figure 9, pp. 16/44) to clearly depict the proposed patch-based transformation pipeline. The figure

visualises the process of generating local patches, applying a sliding window mechanism, unfolding each patch into column vectors, and subsequently

performing kernel density estimation on these transformed representations. This addition improves the interpretability of the data transformation procedure and clarifies how the probabilistic structure is constructed for clustering.

- Reviewer #1

Thanks the authors. All comments are answered properly and the article can be published in the present form.

- Authors’ Response: We are deeply grateful to the Reviewer for their thorough evaluation and for the favorable recommendation regarding the publication of our manuscript. We sincerely appreciate the time and expertise the Reviewer has dedicated to the review process.

- Reviewer #3

1. The abstract reports very strong performance, including an ARI of 1.00 and lower execution times, but it does not provide enough quantitative

comparison against specific baseline methods. Naming the main competing algorithms and indicating the magnitude of improvement would make the claims more convincing.

- Authors’ Response: We sincerely appreciate the Reviewer’s constructive observation. We concur that the initial version of the abstract lacked sufficient detail to fully support the claims of superiority. Therefore, we have revised the abstract to clearly specify the main competing algorithms (FCF, FCF-L1, KMEANS, and

Self-Updating) and to include the magnitude of improvements in terms of ARI, NMI, and computational time across different experimental settings.

- Authors’ Action: We have revised the abstract to explicitly describe the methodological novelty of the proposed framework. Specifically, we have updated the

abstract to explicitly list the baseline methods used for comparison and report quantitative improvements. Specifically, the revised version now highlights that BKIFF achieves near-perfect ARI (up to 1.00), improves ARI by approximately 30–35% in moderately imbalanced cases and from near-zero in highly imbalanced scenarios, increases NM I by about 25–95%, and reduces computational time by approximately 95–99% compared to the baseline methods. These additions provide clearer and more convincing evidence of the superiority of the proposed approach.

2. Although the paper mentions theoretical convergence via Zangwill’s theorem, the abstract does not clearly explain what is improved in the fuzzy

clustering framework beyond the BKO-based initialization. A more explicit statement of the methodological novelty would help distinguish the

proposed contribution from existing optimized fuzzy clustering methods.

- Authors’ Response: We thank the reviewer for this important comment. We have consequently revised the manuscript to explicitly delineate the methodological novelties of the BKIFF framework, thereby clearly distinguishing our contribution from conventional optimised improved fuzzy clustering approaches.

- Authors’ Action: We clarify that BKIFF incorporates the Hellinger distance into the clustering objective to better model similarities between probability density functions, and introduces improved membership updating and prototype estimation mechanisms through the proposed Improved Fuzzy clustering for probability density Functions (IFF), which is designed to effectively handle uncertainty and data imbalance.

3. The introduction should clearly conclude with a distinct section highlighting the novel contributions of your work.

- Authors’ Response: We sincerely appreciate the Reviewer’s constructive suggestion. We concur that a dedicated section outlining the main contributions improves the clarity and structural flow of the Introduction.

- Authors’ Action: We have revised the Introduction to include a dedicated paragraph that explicitly summarises the main contributions of this study, specifically, Contributions (SubSection 1.4, lines 115-150, pp. 5/44).

4. At the ending of the intro, it is advised to add a para that mentions briefly what each next section contains.

- Authors’ Response: We sincerely thank the Reviewer for this constructive comment. Accordingly, we have revised the manuscript by adding a description of its structure.

- Authors’ Action: A paragraph summarizing the structure of the paper has been added at the end of the Introduction, specifically, Organisation (SubSection 1.6, lines 151-158, pp. 5/44)

5. The literature review should benefit from more explorations of previous studies.

- Authors’ Response: We sincerely appreciate the Reviewer’s constructive assessment regarding the scope of the literature review. We have revised the Related Works section to include a more thorough exploration of prior studies.

- Authors’ Action: We have extensively revised and restructured the Related Works section to provide a more comprehensive literature review. Specifically:

• We have provided a more detailed description of representative methods such as Uncertain k-means, Uncertain k-medoids, kernel-based approaches, and density-based methods (lines 42-57, pp. 3/44). Moreover, we have cited additional foundational articles, such as https://doi.org/10.1007/s00778-005-0159-3, and https://doi.org/10.1016/j.neunet.2017.06.004, for further analytical depth.

• We have explicitly analysed the underlying mechanisms and inherent limitations of these existing methods (lines 60-74, pp. 3-4/44).

• We have expanded the discussion on advances in recent algorithms based on evolutionary and adaptive strategies, such as genetic algorithms, differential evolution, and self-updating frameworks (lines 79-81, pp. 4/44).

6. The discussion section needs to be expanded to more thoroughly analyse the results.

- Authors’ Response: We sincerely thank the Reviewer’s constructive recommendation.

- Authors’ Action: We have expanded the Discussion section (Section 6, lines 653-666, pp. 24/44) to provide a more thorough analysis of the results and explicitly analyse the interplay between initialisation strategies, imbalance ratios, and membership regularisation mechanisms.

7. The first paragraph of the conclusion should succinctly summarize the contributions of the study in past tense.

- Authors’ Response: Thank you for your constructive comment.

- Authors’ Action: The first paragraph of the Conclusion section has been carefully revised to succinctly summarize the key contributions of the study, with all descriptions consistently expressed in past tense (lines 717-728, pp. 27/44).

8. The second paragraph of the conclusion should provide clear and actionable future recommendations.

- Authors’ Response: Thank you for your valuable comment. In the original manuscript, these recommendations were presented in a dedicated Future Work subsection within the Discussion.

- Authors’ Action: We have moved and incorporated the key future research directions into the second paragraph of Conclusion (lines 730-739, pp. 27-28/44). Specific recommendations include integrating deep clustering, expanding to contrastive learning for images/video, and applying the density framework to areas such as solar radiation analysis or species distribution modeling. Additionally, we cited two article https://doi.org/10.1371/journal.pone.0317396 which involve the clustering-based SMOTE technique to handle imbalanced data and https://doi.org/10.1371/journal.pone.0340758 on evolutionary undersampling to detect rare events in complex data environments.

9. Equations are not properly cited, please add original references.

- Authors’ Response: We sincerely thank the reviewers for their valuable comments. We have reviewed the entire manuscript and added citations to the Hellinger Distance, Dunn Index, and Silhouette Coefficient.

- Authors’ Action: We have made the following specific updates to the revised manuscript, including

• We added Hellinger (1909) https://doi.org/10.1515/crll.1909.136.210 in (Definition 3, pp. 6/44) to acknowledge the theoretical origin of this measure.

• We added Dunn (1973) https://doi.org/10.1080/01969727308546046 (Section 3.6.3, pp. 13/44) when defining the standard for evaluating the distance between clusters.

• We added Rousseeuw (1987) https://doi.org/10.1016/0377-0427(87)90125-7 (Section 3.6.4, pp. 13/44) to confirm the origin of the method of measuring cluster separation.

10. Why are the sections not numbered? this gives a hard time for the reviewers to do their job, please number each section and each subsection of the article in the second round of review.

- Authors’ Response: We sincerely appreciate the Reviewer’s constructive observation regarding the manuscript’s structural organisation.

- Authors’ Action: All sections and subsections have been numbered and highlighted in the revised manuscript.

Attachment Submitted filename: Response_to_Reviewers_auresp_2.pdf

# Peer review: Decision Letter 2

**DOI:** 10.1371/journal.pone.0349753.r005

**Authors:** Zeashan Khan

5 May 2026

A Black-winged Kite Improved Fuzzy Clustering handling Imbalanced Uncertain Data

PONE-D-25-57245R2

Dear Dr. Che Ngoc,

We’re pleased to inform you that your manuscript has been judged scientifically suitable for publication and will be formally accepted for publication once it meets all outstanding technical requirements.

Within one week, you’ll receive an e-mail detailing the required amendments. When these have been addressed, you’ll receive a formal acceptance letter and your manuscript will be scheduled for publication.

An invoice will be generated when your article is formally accepted. Please note, if your institution has a publishing partnership with PLOS and your article meets the relevant criteria, all or part of your publication costs will be covered. Please make sure your user information is up-to-date by logging into Editorial Manager at Editorial Manager® and clicking the ‘Update My Information' link at the top of the page. For questions related to billing, please contact billing support.

If your institution or institutions have a press office, please notify them about your upcoming paper to help maximize its impact. If they’ll be preparing press materials, please inform our press team as soon as possible -- no later than 48 hours after receiving the formal acceptance. Your manuscript will remain under strict press embargo until 2 pm Eastern Time on the date of publication. For more information, please contact onepress@plos.org.

Kind regards,

Zeashan Hameed Khan, Ph.D.

Academic Editor

PLOS One

Additional Editor Comments (optional):

The revised version has been improved and therefore it can be considered for acceptance.

Reviewers' comments:

Reviewer's Responses to Questions

**Comments to the Author**

1. If the authors have adequately addressed your comments raised in a previous round of review and you feel that this manuscript is now acceptable for publication, you may indicate that here to bypass the “Comments to the Author” section, enter your conflict of interest statement in the “Confidential to Editor” section, and submit your "Accept" recommendation.

Reviewer #3: (No Response)

Reviewer #4: All comments have been addressed

**********

2. Is the manuscript technically sound, and do the data support the conclusions?

The manuscript must describe a technically sound piece of scientific research with data that supports the conclusions. Experiments must have been conducted rigorously, with appropriate controls, replication, and sample sizes. The conclusions must be drawn appropriately based on the data presented.

Reviewer #3: (No Response)

Reviewer #4: Yes

**********

3. Has the statistical analysis been performed appropriately and rigorously?

Reviewer #3: (No Response)

Reviewer #4: Yes

**********

4. Have the authors made all data underlying the findings in their manuscript fully available?

The PLOS Data policy requires authors to make all data underlying the findings described in their manuscript fully available without restriction, with rare exception (please refer to the Data Availability Statement in the manuscript PDF file). The data should be provided as part of the manuscript or its supporting information, or deposited to a public repository. For example, in addition to summary statistics, the data points behind means, medians and variance measures should be available. If there are restrictions on publicly sharing data—e.g. participant privacy or use of data from a third party—those must be specified.

Reviewer #3: (No Response)

Reviewer #4: Yes

**********

5. Is the manuscript presented in an intelligible fashion and written in standard English?

PLOS ONE does not copyedit accepted manuscripts, so the language in submitted articles must be clear, correct, and unambiguous. Any typographical or grammatical errors should be corrected at revision, so please note any specific errors here.

Reviewer #3: (No Response)

Reviewer #4: Yes

**********

6. Review Comments to the Author

Please use the space provided to explain your answers to the questions above. You may also include additional comments for the author, including concerns about dual publication, research ethics, or publication ethics. (Please upload your review as an attachment if it exceeds 20,000 characters)

Reviewer #3: (No Response)

Reviewer #4: Clustering uncertain data is a fundamental problem in data mining. Imbalance among uncertain objects significantly degrades clustering performance, as minority clusters are repeatedly overshadowed by dominant ones. Consequently, existing clustering techniques often fail due to initialisation biases and inadequate similarity modelling. This paper proposes a novel algorithm, the Black-winged Kite Improved Fuzzy clustering for probability density Functions (BKIFF), which combines an optimisation-based initialisation strategy with an enhanced fuzzy clustering framework. Specifically,

BKIFF incorporates the Hellinger distance into the clustering objective to more reliably capture similarities between pdfs, and introduces improved membership updating and prototype estimation mechanisms tailored for uncertain and imbalanced data formulated as Imporved Fuzzy clustering for probability density Functions (IFF) while theoretical convergence is established. In addition, the algorithm employs Black-winged Kite Optimisation (BKO) to enhance prototype selection, improving clustering stability and convergence. As a result, comprehensive experiments with synthetic Gaussian probability distributions, skewed probability density functions (pdfs), and real-world image datasets demonstrate that BKIFF consistently outperforms baseline methods such as FCF, FCF-L1, KMEANS, and Self-Updating. Across all three examples, BKIFF achieves near-perfect ARI, improving from near-zero values in highly imbalanced cases {20, 50, 80, 100} by approximately 30–35% in moderate settings,

while increasing NMI by about 25–95%. Additionally, it reduces computational time by approximately 95–99% compared to baseline methods. In conclusion, BKIFF demonstrates superior performance and opens up new possibilities for applications in medical diagnostics, ecological analysis, and high-dimensional uncertain data mining, particularly in imbalanced environments.

The authors have done well the revision work.

**********

7. PLOS authors have the option to publish the peer review history of their article (what does this mean?). If published, this will include your full peer review and any attached files.

If you choose “no”, your identity will remain anonymous but your review may still be made public.

**Do you want your identity to be public for this peer review?** For information about this choice, including consent withdrawal, please see our Privacy Policy.

Reviewer #3: No

Reviewer #4: No

**********

# Editor report: Acceptance letter

**DOI:** 10.1371/journal.pone.0349753.r006

**Authors:** Zeashan Khan

PONE-D-25-57245R2

PLOS One

Dear Dr. Che-Ngoc,

I'm pleased to inform you that your manuscript has been deemed suitable for publication in PLOS One. Congratulations! Your manuscript is now being handed over to our production team.

At this stage, our production department will prepare your paper for publication. This includes ensuring the following:

* All references, tables, and figures are properly cited

* All relevant supporting information is included in the manuscript submission,

* There are no issues that prevent the paper from being properly typeset

You will receive further instructions from the production team, including instructions on how to review your proof when it is ready. Please keep in mind that we are working through a large volume of accepted articles, so please give us a few days to review your paper and let you know the next and final steps.

Lastly, if your institution or institutions have a press office, please let them know about your upcoming paper now to help maximize its impact. If they'll be preparing press materials, please inform our press team within the next 48 hours. Your manuscript will remain under strict press embargo until 2 pm Eastern Time on the date of publication. For more information, please contact onepress@plos.org.

You will receive an invoice from PLOS for your publication fee after your manuscript has reached the completed accept phase. If you receive an email requesting payment before acceptance or for any other service, this may be a phishing scheme. Learn how to identify phishing emails and protect your accounts at https://explore.plos.org/phishing.

If we can help with anything else, please email us at customercare@plos.org.

Thank you for submitting your work to PLOS ONE and supporting open access.

Kind regards,

PLOS ONE Editorial Office Staff

on behalf of

Dr. Zeashan Hameed Khan

Academic Editor

PLOS One
